# Supplementary material for: Thoracic outlet syndrome (TROTS) registry: A study protocol for the primary upper extremity deep venous thrombosis section
Source: PLoS One. 2023 Jan 6;18(1):e0279708. doi: 10.1371/journal.pone.0279708 (PMC9821680; doi:10.1371/journal.pone.0279708)
Supplement: S1 File — (PDF) [file pone.0279708.s001.pdf]

**The TROTS registry collaborators**

**Remy H.H. Bemelmans** MD PhD, Department of Internal Medicine, Ziekenhuis Gelderse Vallei, Ede, the Netherlands.
**Peter E. Westerweel** MD PhD, Department of Internal Medicine, Albert Schweitzer Hospital, Dordrecht, the Netherlands.
**Maarten Lijkwan** MD PhD, Department of Surgery, Albert Schweitzer Hospital, Dordrecht, the Netherlands.
**Anne C. Esselink** MD, Department of Internal Medicine, Canisius Wilhelmina Ziekenhuis, Nijmegen, The Netherlands.
**Aron S. Bode** MD PhD, Department of Surgery, Canisius Wilhelmina Ziekenhuis, Nijmegen, The Netherlands.
**Çağdaş Ünlü** MD PhD, Department of Vascular Surgery, Noordwest-Ziekenhuisgroep, Alkmaar, Netherlands.
**Hinke Nagtegaal** Msc, Department of internal medicine, Noordwest-Ziekenhuisgroep, Alkmaar, The Netherlands.
**Arina ten Cate** MD PhD, Thrombosis Expertise Center, Maastricht University Medical Center and Cardiovascular Research Institute Maastricht (CARIM), Maastricht, The Netherlands.
**Jorinde van Laanen** MD, Department of Vascular Surgery, Maastricht University Medical Center, Maastricht, The Netherlands.
**Arian van der Veer** MD PhD, Department of pediatric hematology, Maastricht University Medical Center+, Maastricht, The Netherlands.
**Vincent van Weel** MD PhD, Department of Surgery, Meander Medical Center, Amersfoort, The Netherlands.
**Gerben C. Mol** MD, Department of Internal Medicine, Meander Medical Center, Amersfoort, The Netherlands.
**Jasper Florie** MD PhD, Department of Radiology, Meander Medical Center, Amersfoort, The Netherlands.
**Daniel R. Faber** MD PhD, Department of Internal Medicine, BovenIJ hospital, Amsterdam, the Netherlands.
**Jeroen K. de Vries** MD, Department of Internal Medicine, Antonius hospital, Sneek, The Netherlands.
**Robertus HW van de Mortel** MD, Department of Vascular Surgery, Antonius hospital, Nieuwegein, The Netherlands.
**Marijke Molegraaf** MD PhD, Department of Vascular Surgery, Isala Hospital, Zwolle, the Netherlands.
**Vincent Jongkind** MD PhD, Department of Surgery, Amsterdam UMC vrije Universiteit Amsterdam, Amsterdam, The Netherlands. Amsterdam Cardiovascular Sciences, Microcirculation, Amsterdam, The Netherlands. Department of Physiology, Amsterdam Cardiovascular Sciences, Vrije Universiteit Amsterdam, Amsterdam, The Netherlands.
**Kakkhee Yeung** MD PhD, Department of Surgery, Amsterdam UMC vrije Universiteit Amsterdam, Amsterdam, The Netherlands. Amsterdam Cardiovascular Sciences, Microcirculation, Amsterdam, The Netherlands. Department of Physiology, Amsterdam Cardiovascular Sciences, Vrije Universiteit Amsterdam, Amsterdam, The Netherlands.
**Michiel Coppens** MD PhD, Department of Vascular Medicine, Amsterdam Cardiovascular Sciences, Amsterdam University Medical Centers, University of Amsterdam, Amsterdam, The Netherlands.

**Koen E.A. van der Bogt** MD PhD, Department of vascular surgery, Haaglanden Medical Center, Den Haag, the Netherlands.
**Edith D. Beishuizen** MD PhD, Department of internal medicine, Haaglanden Medical Center, Den Haag, the Netherlands.
**C. Heleen van Ommen** MD PhD, Department of Pediatric Hematology, Sophia Children's Hospital ErasmusMC, Rotterdam, The Netherlands.
**Marieke J.H.A. Kruip** MD PhD, Erasmus MC department of hematology, Erasmus University Medical Center, Rotterdam, the Netherlands.
**Marie Josee E van Rijn** MD PhD, Department of Vascular and Endovascular Surgery, Erasmus University Medical Center, Rotterdam, The Netherlands.
**Thomas van Bemmel** MD PhD, Department of internal medicine, Gelre hospitaal, Apeldoorn, The Netherlands.
**Peter L. Klemm** MD PhD, Department of Vascular Surgery, Gelre Hospitaal, Apeldoorn, The Netherlands.
**Marcel M.C. Hovens** MD PhD, Department of internal medicine, Rijnstate hospital, Arnhem, The Netherlands.
**Paul M. van Schaik** MD PhD, Department of Surgery, Rijnstate hospital, Arnhem, The Netherlands.
**Matthijs Eefting** MD PhD, Department of internal medicine, Ikazia Hospital, Rotterdam, the Netherlands.
**Anne M.E. van Well** MD, Department of vascular surgery, Ikazia Hospital, Rotterdam, the Netherlands.
**Roos C. van Nieuwenhuizen** MD, Department of Vascular Surgery, Onze Lieve Vrouwen Gasthuis, Amsterdam, the Netherlands.
**S. van Wissen** MD PhD, Department of Internal Medicine, Onze Lieve Vrouwen Gasthuis, Amsterdam, the Netherlands.
**Martine C.M. Willems** MD PhD, Department of Surgery, Flevoziekenhuis, Almere, The Netherlands.
**Judith P. Post** MD, Department of Internal Medicine, Flevoziekenhuis, Almere, The Netherlands.
**Fleur S. Kleijwegt** MD PhD, Department of Internal Medicine, Rode Kruis Hospital, Beverwijk, The Netherlands.
**Monique H. Suijker** MD, Department of Paediatric Haematology, University Medical Center Utrecht, Utrecht University, Utrecht, the Netherlands. Van Creveldkliniek, University Medical Center Utrecht, Utrecht, the Netherlands.
