# Supplementary material for: Thoracic outlet syndrome (TROTS) registry: A study protocol for the primary upper extremity deep venous thrombosis section
Source: PLoS One. 2023 Jan 6;18(1):e0279708. doi: 10.1371/journal.pone.0279708 (PMC9821680; doi:10.1371/journal.pone.0279708)
Supplement: S2 File — (PDF) [file pone.0279708.s002.pdf]

LOGO

Patient information form for participation in a registry for medical scientific research

Title: Thoracic Outlet Syndrome registry

Introduction

Dear sir/madam,

In this letter we ask you if you would like to participate in the above registration (see title) in which data is collected for future medical scientific research. Participation is voluntary. Your permission is required in order to participate. You are receiving this letter because you have been diagnosed with Thoracic Outlet Syndrome (TOS) or arm thrombosis.

Thoracic Outlet Syndrome is a collective term for conditions in which the vein, artery and/or nerve is/are trapped in the shoulder area.

Sometimes patients with TOS develop an arm thrombosis. An arm thrombosis can also occur without the presence of TOS.

Before you decide whether you want to participate in this study, it will be explained to you what the study entails. Please read this information carefully and ask the researcher to explain further if you have any questions. You can also talk about it with your partner, friends, or family.

1. General information

This study is a collaboration of several hospitals, including ‘Name Hospital’. The research is coordinated and conducted by the University Medical Center Utrecht, the Netherlands (UMC Utrecht).

For scientific research it is of great importance that (medical) data from patients is collected and stored. We call this a registration. At the time of collection it is not yet known for which specific research the data will be used. In general, it concerns research into the cause of disease, research that can lead to better diagnosis of disease, research that can better predict disease, and research that can contribute to the development of new treatments.

2. Purpose of this registration

For future research on TOS and arm thrombosis, we want to collect medical data from patients with this condition to gain insight into the treatment outcome and its impact on the patient's quality of life. Given the rare nature of TOS and arm thrombosis, the aim of this registry is to ask patients internationally to participate in order to collect as much data as possible.

The specific treatment you receive will <u>not</u> be affected by participating in this registry.

3. What participation entails

If you choose to participate in this registration, we ask the following of you:

1. Permission to ‘name hospital’ to collect (medical) data, such as :

- Some basic data of the patient: gender, age, history, etc.

- Basic data with regard to the disease: affected side, pattern of symptoms, possible imaging, etc.

- Data regarding the treatment: medication, interventions, and operations.

- Treatment outcome, including questionnaires.

2. Permission to share your coded data (this is data that cannot be traced back to your person) with the research provider; the UMC Utrecht, the Netherlands.
As part of this registration, your data will be stored in the future and used for the purposes described in the information letter.

3. Permission to send questionnaires to measure treatment outcome and quality of life. These questionnaires will be sent electronically to your email address. The questionnaires will be send immediately after you decide to participate, then after 1, 2, and 5 years, and then every 5 years. The questionnaires will be sent using the program Castor, Castor is an online system in which the UMC Utrecht collects the data. Completing these questionnaires will take about 15 minutes each time.

These questionnaires are not part of the 'standard care'; therefore they are purely for the purpose of this registration.

4. Permission to contact you to provide additional information, if necessary for a particular future scientific study.

If you answer 'no' here, this will be noted with your data.

5.Permission to request information from the governments personal records database.

In order to stay informed about the correct personal details in the future, we would like to ask your permission to request information from the, if necessary, from the municipality where you live. Naturally, we will respect your wishes if you do not give us permission to do so. We need the personal data, for example, to be able to inform you in the event of findings as we explain later in this letter. If you do not give permission to request data from the personal records database in the future, this may mean that we are unable to contact you in the event of a finding.

6. Permission to retrieve and use your medical records.

With medical data from your treatment record now and in the future, we can better study the causes and complications of various diseases. This also applies to medical data from the treatment files of your general practitioner or from other hospitals if you have been treated there. Your medical data will always be made available by the data manager of the registry in coded form (i.e. made unrecognizable) before they are used by the researcher. In this way we ensure that the researcher cannot find out your identity.

7. Permission to request cause of death data from 'the responsible authority'. 'the responsible authority' is the official body in the 'country' that registers causes of death.

If you should die, we would like to inquire with 'the responsible authority' about the cause of your death.

8. Permission to share your medical information that we have collected, anonymously, with other participating hospitals, for further research into your condition.

The (medical) data collected from you will remain available for research without restriction, unless you withdraw your consent. In the event of your death, your consent will remain in full force and your next of kin will have no say in this matter.

4. Possible advantages and disadvantages

Participation in this registry provides no direct benefit to yourself. The results of research using the data in this registry will not be provided back to you. However, results of the study may improve your care and that of other people with similar conditions in the future.

A disadvantage of participating in this registry is that it takes time to complete the questionnaires.

5. If you do not wish to participate or wish to discontinue your participation in this registration

You decide whether to participate in this registration. Participation is voluntary. If you do not wish to participate, you will simply receive the same treatment as usual. If you do participate, you can always

change your mind and withdraw your consent at any time without providing reasons. You can do this by sending the attached withdrawal form to the head of the vascular surgery or internal medicine department.

6. Use of your data

For this registration, your personal data will be collected, used, and stored. This includes data such as your name, date of birth, and data concerning your health. The collection, use, and storage of your data is necessary to answer questions for future medical scientific research and to publish the results. ‘Name hospital’ collects your data and then shares them in an encrypted manner, i.e. not directly traceable to you, with the research institute; the UMC Utrecht. Further research with your data will only be conducted under the responsibility of researchers appointed at the UMC Utrecht. The research may also be conducted in collaboration with other (foreign) hospitals in which case a researcher appointed by UMC Utrecht always remains involved. For such research it may be necessary to provide (medical) data to these hospitals. However, this will always be done in such a way that the data cannot be traced back to you.

In reports and publications about the research the data will also not be traceable to you. In processing your data we adhere to the General Data Protection Regulation. You can read exactly what this means for you in the appendix. We ask you for your permission to use your data.

6. No compensation for participation

You will not be reimbursed for participating in this registration.

7. Do you have any questions?

If you have any questions, please contact your treating physician or the coordinating researcher: ‘name local principal investigator’.

If you have complaints about the registration, you can discuss this with the researcher or your treating physician. If you prefer not to, you can contact the complaints mediators.

‘Contact details local complaints committee’

8. Signature of informed consent

When you have had sufficient time to reflect, you will be asked to decide whether to participate in this registration. If you give your consent, we will ask you to confirm it in writing on the accompanying

informed consent form. Your written consent indicates that you have understood the information and agree to participate in the registry.

The signature sheet will be retained by the investigator. You will receive a copy of this consent form.

Thank you for your consideration.

Sincerely, on behalf of the Thoracic Outlet Syndrome registry,

‘Name local principal investigator’

Attachments:

1. Additional information about processing of your data
2. Informed consent form
3. Withdrawal form

Attachment 1: Additional information about processing of your data

Confidentiality of your data

To protect your privacy, your data will be coded. Your name and other data that can directly identify you are omitted. Only with the keyfile containing the code linked with your personal data, data can be traced back to you. The keyfile will remain safely stored at the local research institution; ‘name hospital’. Also in reports and publications about the research the data cannot be traced back to you.

Access to your data for monitoring purposes

Some individuals can access all your data at the research site. Also to the data without a code. This is necessary to be able to check whether the study was carried out properly and reliably. People who can see your data are ‘the responsible authority’. They will keep your data confidential. We ask you to give us your permission for this inspection.

Data storage period

Your data will be kept at the research site for an indefinite period of time

Withdrawal of consent

You can always withdraw your consent for the use of your personal data.

If you withdraw your consent, this means that no new (medical) data will be collected. In addition, you can choose between two options:

1) The (medical) data collected up to that point will remain available for scientific research as established in the consent form.

2) You request that no (medical) data will be used for future research with this registration.

If you explicitly request this, all (medical) data collected from you for this registration will be destroyed, except for data that has already been used in scientific research.

Sharing of data

In the context of collaborations in this registration, your data may be transferred for analysis to other countries. Outside the EU, the EU rules on the protection of your personal data do not apply. However,

your privacy will be protected at an equivalent level. Your data will only be transmitted in encrypted form.

Additional information about your rights in the processing of your personal data

If you have any questions about your rights, please contact the person responsible for processing your personal data. For this research this is:

‘Contact details local principal investigator’

If you have any questions or complaints about the processing of your personal data, we recommend that you first contact ‘contact details local principal investigator’. You can also contact ‘local contact details data protection officer/complaints commissioner etc’.

Attachement 2: Informed consent form

Thoracic Outlet Syndrome registry

- I have I have read the information letter. I was also able to ask any questions. My questions were adequately answered. I had enough time to decide whether to participate.

- I know that participation is voluntary. I also know that I can decide at any time not to participate or to stop with the registration. I do not have to give a reason for doing so.

- I consent to the collection and use of my (medical) data for scientific research in the field of Thoracic Outlet Syndrome / arm thrombosis as described in the information letter.

- I give permission to keep the collected (medical) data indefinitely for future scientific research for the purposes described in the information letter.

- I know that for purposes of verifying this registration, some people may have access to all of my information. Those people are listed in the attachement to the information letter. I give my permission for access by those people.

- I give permission for the transfer of my data in the context of this registration. Data must be transferred in encrypted form, without my name and other personal data that can directly identify me.

- I give permission for the questionnaire to be sent to my email address. The questionnaires will be sent form and to the program Castor.

<u>Please mark what is applicable</u>

- I ◻ **give my consent**
  ◻ **do not give my consent**

  to contact me during this registration for follow-up research.

- I ◻ **give my consent**
  ◻ **do not give my consent**

  to be contacted to provide additional data, if necessary for a particular study

- I ◻ **give my consent**
  ◻ **do not give my consent**

to request my personal data from the governments personal records database if necessary in the future.

- I □ **give my consent**
  □ **do not give my consent**

  to request medical information from my primary care physician and other hospitals where I have been treated, if necessary, in the future.

- I □ **give my consent**
  □ **do not give my consent**

  permission to request my cause of death from ‘the responsible authority’ if necessary in the future

- I □ **give my consent**
  □ **do not give my consent**

  to the transfer of my encrypted data to countries outside the EU where EU rules on the protection of your personal data do not apply

- I want to participate in this study.

Please provide the details on the dotted lines

Name participant: _

Email address participant: _

Signature: _

Date (dd-mm-yyyy) : __ / __ / __

I declare that I have fully informed this subject about the study.

If any information becomes known during the study that could affect the subject's consent, I will inform him/her in a timely manner.

Name researcher (or its representative): _

Signature: _

Date (dd-mm-yyyy): __ / __ / __

The subject will receive a complete information letter along with a copy of the signed consent form.

Attachment 3: Withdrawal form

Thoracic Outlet Syndrome registry

I hereby give notice that I withdraw my participation in this registration. This means that no new (medical) data may be collected from me and that no questionnaires will be administered for future research into Thoracic Outlet Syndrome or arm thrombosis.

In addition, I would like that:

□ The (medical) data collected so far will remain available for scientific research as described in the consent form.

□ That no more (medical) data of mine will be used for the purpose of research with this registration, including the data already collected.

I understand that (medical) data already used and published in a study cannot be retrieved or destroyed.

Name: ____

Date of birth: __________________________

Date:______________________ Signature:__________________

Send form to head of vascular surgery/internal medicine department:

____

I declare that I have taken note of the withdrawal of consent by the patient listed above and as described above.

Institution: ‘name hospital’

Department head name: ______________________________

Date: ____________ Signature: ______________________
