# Supplementary material for: Thoracic outlet syndrome (TROTS) registry: A study protocol for the primary upper extremity deep venous thrombosis section
Source: PLoS One. 2023 Jan 6;18(1):e0279708. doi: 10.1371/journal.pone.0279708 (PMC9821680; doi:10.1371/journal.pone.0279708)
Supplement: S3 File — (PDF) [file pone.0279708.s003.pdf]

Thoracic outlet syndrome registry
(nWMO)
dHS research

Version 1.0; date 03.05.2021

Correspondence
G.J. de Borst MD PhD, vascular surgeon

Department of Vascular surgery, University Medical Center Utrecht,

Heidelberglaan 100, 3584 CX Utrecht, the Netherlands

G.J.DeBorst-2@umcutrecht.nl

PROTOCOL TITLE:

**Short title**	**TOS registry**
**Version:**	**1.0**
**Date**	**03.05.2021**
**Coordinating investigator/project leader**	Prof. dr. G.J. de Borst, vascular surgeon<br>Heidelberglaan 100<br>3584 CX Utrecht<br>The Netherlands<br>E-mail:G.J.deBorst-2@umcutrecht.nl
**Principal investigator (in Dutch: hoofdonderzoeker/ uitvoerder)**	Prof. dr. G.J. de Borst, vascular surgeon<br>Heidelberglaan 100<br>3584 CX Utrecht<br>The Netherlands<br>E-mail:G.J.deBorst-2@umcutrecht.nl
**Other investigator(s)**	**Dr. B.J. Petri**<br>**Dr. E.S. van Hattum**<br>**Dr. R.J. Toorop**<br>**Dr. C.E.V.B. Hazenberg**<br>**Prof. Dr. J.A. van Herwaarden**<br>**Drs. R.J.C.M.F. de Kleijn**<br>**Drs. L. Schropp**
**Sponsor (in Dutch: verrichter/opdrachtgever)**	**UMC Utrecht**

PROTOCOL SIGNATURE SHEET

Name	Signature	Date
**Head of Department:**<br>Prof. dr. G.J. de Borst, vascular surgeon<br>Heidelberglaan 100<br>3584 CX Utrecht<br>The Netherlands<br>E-mail:G.J.deBorst-2@umcutrecht.nl		
**Principal Investigator:**<br>Prof. dr. G.J. de Borst, vascular surgeon<br>Heidelberglaan 100<br>3584 CX Utrecht<br>The Netherlands<br>E-mail:G.J.deBorst-2@umcutrecht.nl		

TABLE OF CONTENTS

1. INTRODUCTION AND RATIONALE 7
2. OBJECTIVES 8
3. REGISTRY DESIGN 8
4. REGISTRY POPULATION 8
   - 4.1 Population (base) 8
   - 4.2 Inclusion criteria 8
   - 4.3 Exclusion criteria 8
5. METHODS 9
   - 5.1 Parameters/endpoints 9
     - 5.1.1 Main parameter/endpoint 9
   - 5.2 Registry procedures 9
   - 5.3 Withdrawal of individual subjects 9
     - 5.3.1 Specific criteria for withdrawal (if applicable) Participation is voluntarily, the patient decides whether to participate or not. 10
   - 5.4 Replacement of individual subjects after withdrawal As it concerns a growing group of participants, no participants will be replaced. 10
   - 5.5 Follow-up of subjects withdrawn from treatment 10
6. ETHICAL CONSIDERATIONS 11
   - 6.1 Regulation statement 11
   - 6.2 Recruitment and consent 11
7. ADMINISTRATIVE ASPECTS, PRIVACY INCIDENTS AND END OF REGISTRY REPORT 12
   - 7.1 Handling and storage of data and documents 12
   - 7.2 Ethical Committee 13
   - 7.3 Privacy incidents 13
   - 7.4 End of registry report 13
   - 7.5 Publications 13
8. REFERENCES 15
9. Appendix 16
   - 9.1 List of parameters included in the registry 16

LIST OF ABBREVIATIONS AND RELEVANT DEFINITIONS

**aTOS**	**Arterial thoracic outlet syndrome**
**AVG**	**Algemene Verordening Gegevensbescherming**
**CDT**	**Catheter direct thrombolysis**
**DOAC**	**Direct oral anticoagulants**
**ERN**	**European Reference Networks**
**IC**	**Informed Consent**
**nTOS**	**Neurogenic thoracic outlet syndrome**
**PIF**	**Patient information form**
**PTA**	**Percutaneous transluminal angioplasty**
**RCT**	**Randomized controlled trials**
**TOD**	**Thoracic outlet decompression**
**TOS**	**Thoracic outlet syndrome**
**UMCU**	**University Medical Centre Utrecht**
**vTOS**	**Venous thoracic outlet syndrome**
**WGBO**	**Law on medical treatment agreement (in Dutch: Wet op Geneeskundige BehandelingsOvereenkomst)**

SUMMARY

Rationale: Thoracic outlet syndrome (TOS) is a rare disease that is caused by compression of the neurovascular bundle in the thoracic outlet region. Based on the compressed structure we can distinguish three types of TOS; from most common to least common these are neurogenic (nTOS), venous (vTOS) and arterial (aTOS). TOS can be treated conservatively through physical therapy and/or anticoagulation therapy with direct oral anticoagulants (DOAC), or invasively with thoracic outlet decompression surgery (TOD) where the first rib is routinely removed. In a- and vTOS patients additional therapy can consist of medical or mechanical thrombus removal and surgical interventions to restore vascular patency such as thrombolysis, stenting, percutaneous transluminal angioplasty or a surgical bypass. Due to the low incidence and the lack of high quality data on all three TOS forms but especially vTOS and aTOS, little is known on the optimal treatment of these patients.
Creating an ongoing registry that supports collective prospective patient data gathering and sharing is a proven method to perform research on medical disorders presenting with a very low incidence. This registry is an online registry for data collection on all patients with TOS.
Objective: To collect clinical data that can be used for future research on optimising diagnostic protocols and treatment strategies, treatment safety, symptom-free survival, quality of life, and functional disability among all three forms of TOS patients.
Registry design: This is a longitudinal observational monocenter registry.
Registry population: All adult patients diagnosed with aTOS, nTOS or vTOS who are currently being treated or have been treated in the University Medical Centre Utrecht (UMCU) are eligible for inclusion. In addition, patients with a primary upper extremity deep venous thrombosis without a clear vTOS compression will also be included.
Main parameters/endpoints: Data will be collected for future research of which the exact research question is not known at this time. When new research will be conducted, study parameters will be set. A new study application has to be submitted to the METC.
Nature and extent of the burden and risks associated with participation, benefit and group relatedness: The burden for patients to participate in this registry is minimal and solely consists of time. Any future results may be beneficial for subsequent patients. Participation or refusal to participate in the registry will neither have consequences for their treatment.

1. INTRODUCTION AND RATIONALE

The thoracic outlet syndrome (TOS) refers to a series of signs and symptoms caused by compression of the neurovascular bundle in the thoracic outlet. The thoracic outlet is the anatomical space through which the brachial plexus, subclavian artery and subclavian vein pass from the thorax to the arm. We can distinguish three TOS forms based on the compressed structures. Neurogenic thoracic outlet syndrome (nTOS) is the most common form and accounts for approximately 90-95% of all TOS patients, whereas venous TOS (vTOS) (5-10%) and arterial TOS (aTOS) (<1%) are less common.(1)

All three TOS forms can be treated conservatively and invasively. However, very little is known regarding risk factors, optimal treatment strategies, and long-term treatment results such as symptom free survival and vascular patency.(2-12) Consequently, setting up a registry can provide a way to collect high quality long term follow-up data for future research. By initiating this new registry, we eventually aim to create an international cooperation linking expert centres on TOS.

2. OBJECTIVES

Primary Objective: To create a registry that can be used for future research on optimising diagnostic protocols, treatment strategies, improving symptom-free survival and optimising patient follow-up among all three forms of TOS patients.

3. REGISTRY DESIGN

This is a monocenter longitudinal observational registry. The duration is for an indefinite amount of time.

4. REGISTRY POPULATION

4.1 Population (base)

Adult patients (aged 18 years and older), who are diagnosed with TOS and are currently being treated or have been treated at the UMCU in the Netherlands, are eligible for this registry. In addition, patients with a primary upper extremity deep venous thrombosis without a clear vTOS compression will also be eligible.

4.2 Inclusion criteria

In order to be included in this registry, a subject must meet all of the following criteria:

- A positive diagnosis for nTOS, or aTOS;
- Primary upper extremity deep vein thrombosis, including vTOS;
- Aged 18 years and older;
- Signed informed consent from patients and/or their legal representative

4.3 Exclusion criteria

A potential subject who meets any of the following criteria will be excluded from participation in this registry:

- Secondary upper extremity deep venous thrombosis;
- Individuals who are unwilling to sign complete or partial informed consent.

5. METHODS

5.1 Parameters/endpoints

5.1.1 Main parameter/endpoint

The main parameters of interest include, but are not limited to:

- Patient and treatment characteristics
- Symptom-free survival
- Vascular patency
- Treatment related complications
- Health-related Quality of Life (EQ-5D-5L) and Functional Disability questionnaire (Quick-DASH) sent at the time of inclusion and then after 1, 2, 5 years and then every 5 years.

The full list of parameters is presented in appendix 1. Depending on new insights on TOS these endpoints may be altered and updated.

5.2 Registry procedures

A patient information form (PIF) will be provided to the participant as well as verbal information by the local principal investigator at the first hospital visit. In addition, a written, dated, and signed broad consent form will be obtained by the participant and local principal investigator. After obtaining broad consent, each participant is assigned a unique study ID. Consequently, all relevant clinical information will extracted from the electronic health records (EHRs) and entered in the register's database in Castor EDC by members of the research team. The above mentioned questionnaires (see 5.1.1.) will be automatically sent via Castor by e-mail at set time points, namely at the time of inclusion, 1, 2 and 5 years after inclusion and then every 5 years. It will take the participant approximately 15 minutes to complete the self-administered questionnaires. In case of no response, patients will receive a reminder after 14 days to complete the questionnaire. The treatment, additional diagnostic imaging and laboratory test that subjects will undergo are standard care and indicated by the treating physician in accordance with the latest guidelines and will not be influenced by participation in the registry.

5.3 Withdrawal of individual subjects

Subjects can leave the registry at any time for any reason if they wish to do so without any consequences. They can submit the withdrawal form that is included in the patient information. Subjects can either chose to exclude their data from further research or to allow the data that was collected before withdrawal to be used in further research. If a subject choses to excluded their data from further research this data will be destroyed.

5.3.1 Specific criteria for withdrawal (if applicable)

Participation is voluntarily, the patient decides whether to participate or not.

5.4 Replacement of individual subjects after withdrawal

As it concerns a growing group of participants, no participants will be replaced.

5.5 Follow-up of subjects withdrawn from treatment

There will be no follow-up of subjects that are withdrawn from the registry and it has no consequences for any further treatment.

6. ETHICAL CONSIDERATIONS

6.1 Regulation statement

The registry will be designed and implemented according to ‘gedragscode gezondheidsonderzoek’ and ‘toetsingscriteria eenvormige toetsing’ that consist of the laws: ‘WGBO (Wet op de Geneeskundige BehandelingsOvereenkomst)’ and Good Data Protection Regulation (GDPR) (Dutch: Algemene Verordening Gegevensbescherming (AVG)).

6.2 Recruitment and consent

All patients with suspected TOS are discussed in a multidisciplinary team of radiologists, vascular surgeons and internal vascular medicine specialists where the treatment strategy is determined. All patients with a positive diagnosis of TOS will be informed about the registry by their treating physician at their first hospital visit, subsequently they are presented the patient information form (PIF) and informed consent (IC) form. At their next physical visit to the hospital, at least 24 hours later, the patient is presented the opportunity to ask any questions about the registry. If the patient wishes to participate in the registry, informed consent is obtained by the local principal investigator (PI) and the IC form is signed by the patient and PI. The participant is presented a copy of the IC form. The IC forms are stored at a secured location at the department of vascular surgery in the UMCU. Recruitment and consent of the patient is recorded in the electronic health record.

7. ADMINISTRATIVE ASPECTS, PRIVACY INCIDENTS AND END OF REGISTRY REPORT

7.1 Handling and storage of data and documents

Data will be handled confidentially and will be coded according to the order of entry. Confidentiality will be maintained at all times, participant information will not be disclosed to third parties. For this registry, only eligible patients admitted or with an appointment at the UMCU will be asked by their treating physician to participate. Direct identifiable—personal data from these patients will be recorded in an Excel file and stored in a secure research folder from the TOS registry on the UMCU network drive. This is for an overview of which patients are asked to participate.

After given broad consent, patients will also be registered in our electronic health record (EHR, HiX). The original signed broad consent forms will be kept in a binder in a locked closet in a locked room at the department of vascular surgery. Subsequently, the participant is assigned a unique study ID generated by Castor EDC. The key file containing the combination of the participants unique study ID and its personal information (name and date of birth) will be stored at a secured location at the UMCU network drive, only the local principal investigator has access for identification purpose. All relevant clinical information will be manually extracted from the EHR's and entered by the local principal investigator into an electronic Case Report Form (eCRF) the UMCU endorsed system Castor EDC. Castor EDC is a browser-based, metadata-driven EDC software solution and workflow methodology for building and managing online databases. The eCRF contains data items as specified in this research protocol. Modification of the eCRF will be made only if deemed necessary and in accordance with an amendment to the research protocol. Access to the eCRF is password protected and specific roles are assigned (e.g. study coordinator, investigator, etc.). In addition, the upper mentioned questionnaires (see paragraphs 5.1 and 5.2) will be sent automatically to the participants at set times via an email link using Castor. Only UMCU researchers will have access to all the collected data in Castor.

When needed for future research, the research data will be extracted and stored in the file format(s) SPSS and/or R in a secure research folder. The research analyst, data manager, researcher, physician and principal investigator(s) will get access to the research data. To be able to reproduce the research findings and to help future users to understand and reuse the data all changes made to the raw data and all steps taken in the analysis will be documented in syntaxes and by using new versions of the database. The original database will not be altered and is stored on the research network disc of my division. The research

data will be archived on the research network disc of the division for 15 years after the registry has ended. More details can be found in the datamanagement plan: https://dmponline.dcc.ac.uk/plans/69559

7.2 Ethical Committee

Any research that will be conducted with (parts of) the data from this registry will be submitted to the METC. Data will only be used for future research after a positive advice or ‘niet-WMO verklaring’ from the METC.

7.3 Privacy incidents

The person that notices a privacy incident, needs to report this immediately to the Central Coordinator of Information security (Dutch: Centrale Coördinator Informatiebeveiliging) stating the following information: contact information of reporter, description of the incident (including an indication of the impact on privacy) and actions that have been taken to control damage. A privacy incident is a security breach that can lead to loss or unlawful processing of personal data.

7.4 End of registry report

The coordinating investigator will notify the dHS Research Office (DHS onderzoeksbureau@umcutrecht.nl and DHS-datamanagement@umcutrecht.nl) of the end of the registry. The goal of this registry is to create an international database that is unique in both size and long term follow-up. Hence we cannot define an specific end of this study.

7.5 Publications

Researchers will publish results from research with data from this registry in public. Researchers are responsible for the completeness and accuracy of publications. Researchers will comply with the guidelines for ethical publication of results.
If (international) co-operators of this registry want to use registry data for publication, data transfer agreements, nWMO study agreements contracts regarding intellectual property rights will be used. Patients will be informed on the international nature of this registry in the PIF.
The contributors involved are nationally and internationally recognised for their work in this registry. In case a large number of individual authors is not allowed by the guidelines of the journal, all individual contributors will appear in PubMed.
Before submission of a manuscript, every principal investigator and co-investigator will be sent the draft manuscript. The principal (co)investigator will provide the first author with her/his comments within 28 days. Also, he/she indicated whether a co-authorship is

appreciated. If no response has been obtained after 28 days, the principal (co)investigator is assumed not be interested in the manuscript and removed from the author list. Mentioning of the principal (co) investigators in the participating center/participant list remains, unless explicitly indicated that this should not be done.

8. REFERENCES

1. Sanders RJ, Hammond SL, Rao NM. Diagnosis of thoracic outlet syndrome. J Vasc Surg. 2007 Sep;46(3):601–4.
2. Fugate MW, Rotellini-Coltvet L, Freischlag JA. Current management of thoracic outlet syndrome. Curr Treat Options Cardiovasc Med. 2009;11(2):176–83.
3. Chang DC, Rotellini-Coltvet LA, Mukherjee D, De Leon R, Freischlag JA. Surgical intervention for thoracic outlet syndrome improves patient's quality of life. J Vasc Surg. 2009 Mar;49(3):630–7.
4. Thompson RW. Comprehensive management of subclavian vein effort thrombosis. Semin Intervent Radiol. 2012 Mar;29(1):44–51.
5. NIV Richtlijn Antitrombotisch Beleid 2015 [https://internisten.nl/sites/internisten.nl/files/Richtlijn Antitrombotisch beleid_def.pdf].
6. Tait C, Baglin T, Watson H, Laffan M, Makris M, Perry D, et al. Guidelines on the investigation and management of venous thrombosis at unusual sites. Br J Haematol. 2012;159(1):28–38.
7. Kearon C, Akl EA, Comerota AJ, Prandoni P, Bounameaux H, Goldhaber SZ, et al. Antithrombotic therapy for VTE disease: Antithrombotic Therapy and Prevention of Thrombosis, 9th ed: American College of Chest Physicians Evidence-Based Clinical Practice Guidelines. Chest. 2012;141(2 Suppl):e419S-e496S.
8. Baglin T, Bauer K, Douketis J, Buller H, Srivastava A, Johnson G, et al. Duration of anticoagulant therapy after a first episode of an unprovoked pulmonary embolus or deep vein thrombosis: guidance from the SSC of the ISTH. J Thromb Haemost. 2012;10(4):698–702.
9. Kakkos SK et al. European Society for Vascular Surgery (ESVS) 2021 Clinical Practice Guidelines for the Management of Venous Thrombosis. Eur J Vasc Endovasc Surg. 2021;
10. Peek J, Vos CG, Unlu C, van de Pavoordt H, van den Akker PJ, de Vries JPM. Outcome of Surgical Treatment for Thoracic Outlet Syndrome: Systematic Review and Meta-Analysis. Ann Vasc Surg. 2017;40:303–26.
11. Marine L, Valdes F, Mertens R, Kramer A, Bergoeing M, Urbina J. Arterial thoracic outlet syndrome: a 32-year experience. Ann Vasc Surg. 2013 Nov;27(8):1007–13.
12. Cormier JM, Amrane M, Ward A, Laurian C, Gigou F. Arterial complications of the thoracic outlet syndrome: fifty-five operative cases. J Vasc Surg. 1989 Jun;9(6):778–87.

9. Appendix

9.1 Appendix 1: List of parameters included in the registry

Study number
Name referring doctor
Contact details referring doctor
Hospital
Date of birth
Sex
BMI
ASA classification
Vascular history
Cardiac history
Diabetes
Other medical history
Current medication use
Risk factors for UEDVT
Risk factors for TOS
Date start of symptoms
Symptoms specified
Affected side
Dominant side
Activity leading to start of symptoms
Job description
Sports and activities
Physical examination (at presentation and during follow-up) including:

- Wright test
- Adson test
- Roos test
- Military Brace Test

Radiology reports
TOS test results
Lab results
Type of intervention
Intervention details such as:

- Duration of thrombolysis treatment
- Dosage of administered thrombolytics
- Specifications and length of stent
- Time between thrombolysis and surgery.
- All relevant surgery reports
- Anaesthesia reports
- Duration of hospital stay
- Type of anticoagulant
- Duration of anticoagulation treatment

- Physical therapy

DELPHI upper extremity PTS-score
EQ5D-5L
Quick-DASH
Complications such as:

- Surgery related complications
- Complications of endovascular procedures
- Bleeding complications
- Recurrent thrombosis or occlusion
- Neurological complications of the affected limb.
- Pulmonary embolism
- Major Adverse Events
- Death
- Other adverse events.

Follow-up details such as

- Plannend and unplanned hospital visits
- Symptoms during follow-up
- Physical exam during follow-up
- Additional diagnostics during follow-up
- Additional treatment during follow-up
