# Supplementary material for: Thoracic outlet syndrome (TROTS) registry: A study protocol for the primary upper extremity deep venous thrombosis section
Source: PLoS One. 2023 Jan 6;18(1):e0279708. doi: 10.1371/journal.pone.0279708 (PMC9821680; doi:10.1371/journal.pone.0279708)
Supplement: S4 File — (PDF) [file pone.0279708.s004.pdf]

Thoracic Outlet Syndrome Registry - version 244.61

Printed on 07-10-2022 14:51:10 by Ludo Schropp

1. Inclusion - Informed consent

Number	Question	Answers
1.1	Has the patient signed informed consent?<br>*Exclude patient if field's value is equal to No with message: 'Patient must have signed informed consent to be included in the registry'*	◯ Yes<br>◯ No
1.2	Has the participant consented to the transfer of its encrypted data to countries outside the EU?	◯ Yes<br>◯ No<br>◯ Not applicable
1.3	Date informed consent was signed?<br>*Warning shown if field's value is larger than NOW: 'Date cannot be in the future'*	___ ___ ___ *(dd-mm-yyyy)*
1.4	Did you enter the participants e-mail adress in Castor?<br>*Warning shown if field's value is equal to No: 'Please enter the participants emailadress before continuing. Go back to records to provide the emailadress, in the records overview use the options button at the far right of the table.'*	◯ Yes<br>◯ No

2. Etiology - Etiology

Number	Question	Answers
2.1	Patient treated by which specialty?	☐ Internal medicine<br>☐ Hematology<br>☐ Vascular medicine<br>☐ Vascular surgery<br>☐ Other
2.1.1	***If 'Patient treated by which specialty?' is equal to 'Other' answer this question:***<br>Which other specialty	

2.2	Sub-type(s) of thoracic outlet syndrome / idiopatic UEDVT<br>If there is a combination of TOS-subtypes, check the relevant boxes.<br><br>If the patient has a upper extremity deep venous thrombosis without compression or any other secondary cause, please choose idiopatic UEDVT	☐ Neurogenic-TOS<br>☐ Arterial-TOS<br>☐ Venous-TOS, with thrombosis. Including Paget-Schroetter syndrome. > Compression of the vein is present.<br>☐ Venous-TOS, without trombosis (McCleary syndrome)<br>☐ Idiopatic arm DVT > No compression of vein present or not investigated.
2.3	calculation, nTOS	
2.4	calculation aTOS	
2.5	Calculation, vTOS	
2.6	Calculation, pUEDVT	
2.7	calculation, vTOS+pUEDVT	
2.8	Calculation, vTOS+pUEDVT+aTOS	
2.9	Unilateral or bilateral complaints? Please press 'i' for additional information.<br>*Warning shown if field's value is equal to Bilateral: 'Is current episode of complaints unilateral or bilateral? If patient presents with unilateral symptoms with a history of contralteral TOS/UEDVT, please choose unilateral. If patient presents with bilateral active symptoms, please choose bilateral.'*<br>In order to collect complete data, a patient with bilateral TOS/UEDVT will be assigned 2 study numbers.<br><br>Is current episode of complaints unilateral or bilateral?<br><br>If patient presents with unilateral symptoms with a history of contralateral TOS/UEDVT without any current problems to the contralateral extremity, please choose unilateral.<br><br>If patient presents with bilateral active symptoms, please choose bilateral.<br><br>If patient presents with unilateral symptoms, please choose unilateral. If this patient presents with contralateral symptoms during follow-up, a new eCRF and studynumber can be assigned. If this is the second eCRF for this patient, please choose bilateral.	○ Unilateral<br>○ Bilateral
2.9.1	***If 'Unilateral or bilateral complaints? Please press 'i' for additional information.' is equal to 'Bilateral' answer this question:***<br>Bilateral	
2.9.2	***If 'Unilateral or bilateral complaints? Please press 'i' for additional information.' is equal to 'Bilateral' answer this question:***<br>This eCRF corresponds with the	○ Left arm<br>○ Right arm

2.9.3	***If 'Unilateral or bilateral complaints? Please press 'i' for additional information.' is equal to 'Bilateral' answer this question:***<br>Is the current studynumber the first or second study number for this patient?	○ First<br>○ Second
2.9.4	***If 'Unilateral or bilateral complaints? Please press 'i' for additional information.' is equal to 'Bilateral' answer this question:***<br>Please provide the corresponding study number of the contralateral arm	[ ]
2.7.1	***If 'calculation, vTOS+pUEDVT' is equal to '1' answer this question:***<br>Recent or current indwelling device (e.g. central venous catheters (CVC), pacemaker or defibrillator leads) at site of thrombosis?<br>*Exclude patient if field's value is equal to Yes with message: '(Possible) provoked upper extremity deep venous thrombosis, patient cannot participate in this registry'*	○ Yes<br>○ No
2.7.2	***If 'calculation, vTOS+pUEDVT' is equal to '1' answer this question:***<br>Active malignany?<br>*Exclude patient if field's value is equal to Yes with message: '(Possible) provoked upper extremity deep venous thrombosis, patient cannot participate in this registry'*	○ Yes<br>○ No
	**Questionnaire check**	
	**If the next question is answered with 'yes', the questionnaires will be sent to the patient.**	
2.10	Are all the answers given until now correct, did you enter the participants emailadress in Castor and can Castor send the patient specific questionnare ?	○ Yes<br>○ No

3. Baseline - Patient characteristics

Number	Question	Answers
3.1	Year of birth	[ ] *(yyyy)*
3.2	Gender	○ Female<br>○ Male
3.3	calculation, a/vtos/puedvt + female	
3.4	Length in centimeters<br>If unknown, choose 0	[ ] Centimeters

3.5	Weight in kilograms, 1 decimal<br>If unknown, choose 0	[ ] Kilograms
3.6	Handedness	○ Left handed<br>○ Right handed<br>○ Ambidextrous<br>○ Unknown
3.7	Profession<br>If patient is currently in school/studying, please state school/study as profession.	[ ]
3.8	Practices sports?	○ Yes<br>○ No<br>○ Unknown
3.8.1	***If 'Practices sports?' is equal to 'Yes' answer this question:***<br>Which sport(s)	[ ]
3.8.2	***If 'Practices sports?' is equal to 'Yes' answer this question:***<br>Professional athlete?	○ Yes<br>○ No

4. Baseline - Medical history

Number	Question	Answers

4.1	Baseline medical history<br>Only select 'Prior history of TOS / pUEDVT' if current episode is a new episode, or patient has persisting symptoms despite previous therapies without previous intentions to perform additional treatment.<br><br>Examples: Presentation with acute thrombosis in another hospital, put on anticoagulants and referred directly for additional treatment. Do not choose 'prior history of upper extremity deep vein thrombosis'.<br><br>Presentation with acute thrombosis, treatment with oral anticoagulants and compression stockings (without intention to perform additional treatment), referred after 6 months because of severe persisting symptoms. Please choose 'prior history of upper extremity deep vein thrombosis'.<br><br>Previous treatment of vTOS with oral anticoagulation and compression stockings for 6 months. Presents 5 years later with a new episode of thrombosis. Please choose prior history of vTOS.	☐ History of DVT lower extremity<br>☐ History of pulmonary embolus<br>☐ History of hypercoagulable state<br>☐ History of cardiovascular disease<br>☐ History of trauma involving thoracic outlet<br>☐ Prior history of nTOS<br>☐ Prior history of aTOS<br>☐ Prior history of vTOS<br>☐ Prior history of upper extremity deep vein thrombosis<br>☐ Other<br>☐ No medical history
4.1.1	***If 'Baseline medical history' is equal to 'History of hypercoagulable state' answer this question:***<br>History of hypercoagulable state	☐ Factor V Leiden<br>☐ Prothrombin G20210A<br>☐ High factor VIII<br>☐ Factor VII mutation<br>☐ Factor II mutation<br>☐ Protein C deficiency<br>☐ Protein S deficiency<br>☐ Antithrombin deficiency<br>☐ Antiphospholipid antibodies<br>☐ Lupus anticoagulant<br>☐ Anticardiolipin IgG<br>☐ Anticardiolipin IgM<br>☐ Anti-beta-2 glycoproteine IgG<br>☐ Anti-beta-2 glycoproteine IgM<br>☐ Plasminogeen activator inhibitor-1<br>☐ Other
4.1.1.1	***If 'History of hypercoagulable state' is equal to 'Other' answer this question:***<br>Please describe other hypercoagulable state	

4.1.2	***If 'Baseline medical history' is equal to 'History of cardiovascular disease' answer this question:***<br>History of cardiovascular disease	☐ Coronary artery disease<br>☐ Heart faillure<br>☐ Cardiomyopathy<br>☐ Cardiac dysrhythmias<br>☐ Atrial fibrilation/flutter<br>☐ Pacemaker<br>☐ Defibrilator (ICD)<br>☐ Cerebrovascular accident<br>☐ Peripheral vascular disease<br>☐ Aortic aneurysm<br>☐ Other
4.1.2.1	***If 'History of cardiovascular disease' is equal to 'Other' answer this question:***<br>Other history of cardiovascular disease	
4.1.3	***If 'Baseline medical history' is equal to 'History of trauma involving thoracic outlet' answer this question:***<br>Describe trauma to the thoracic outlet	
4.1.4	***If 'Baseline medical history' is equal to 'Prior history of nTOS' answer this question:***<br>History of nTOS, affected side?	○ Ipsilateral<br>○ Contralateral<br>○ Bilateral
4.1.5	***If 'Baseline medical history' is equal to 'Prior history of nTOS' answer this question:***<br>Specify history of nTOS including treatment received	
4.1.6	***If 'Baseline medical history' is equal to 'Prior history of aTOS' answer this question:***<br>History of aTOS, affected side?	○ Ipsilateral<br>○ Contralateral<br>○ Bilateral
4.1.7	***If 'Baseline medical history' is equal to 'Prior history of aTOS' answer this question:***<br>Specify history of aTOS including treatment received	
4.1.8	***If 'Baseline medical history' is equal to 'Prior history of vTOS' answer this question:***<br>History of vTOS, affected side?	○ Ipsilateral<br>○ Contralateral<br>○ Bilateral

4.1.9	***If 'Baseline medical history' is equal to 'Prior history of vTOS' answer this question:***<br>Specify history of vTOS including treatment received	
4.1.10	***If 'Baseline medical history' is equal to 'Prior history of upper extremity deep vein thrombosis' answer this question:***<br>History of UEDVT, affected side? (excluding vTOS)	◯ Ipsilateral<br>◯ Contralateral<br>◯ Bilateral
4.1.11	***If 'Baseline medical history' is equal to 'Prior history of upper extremity deep vein thrombosis' answer this question:***<br>Specify history of UEDVT including treatment received (excluding vTOS)	
4.1.12	***If 'Baseline medical history' is equal to 'Other' answer this question:***<br>Please specify other medical history	
4.2	ipsilateral TOS/UEDVT	
	The following questions concern the currently affected arm	
4.2.2	***If 'ipsilateral TOS/UEDVT' is equal to '1' answer this question:***<br>Patient presented/reffered because of residual complaints or a new episode of complaints?<br>Please choose new episode of complaints if patient had a history of TOS/UEDVT with complete resolution of symptoms and now presents with a new episode of complaints.<br><br>Please choose residual complaints if the patient has a history of TOS/UEDVT, the primary treatment has finished, there was no resolution of symptoms and patient now presents with residual complaints.<br><br>Examples:<br><br>Presentation with acute thrombosis, treatment with oral anticoagulants and compression stockings (without intention to perform additional treatment), referred after 6 months because of severe persisting symptoms. Please choose: residual complaints.<br><br>Previous treatment of vTOS with oral anticoagulation and compression stockings for 6 months. Presents 5 years later with a new episode of thrombosis. Please choose: new episode of complaints.	◯ New episode of complaints<br>◯ Residual complaints<br>◯ Other

4.2.2.1	***If 'Patient presented/reffered because of residual complaints or a new episode of complaints?' is equal to 'Other' answer this question:***<br>Please describe other reason for presentation	
4.2.3	***If 'ipsilateral TOS/UEDVT' is equal to '1' answer this question:***<br>Date prior diagnosis was made<br>If precise date is unknown:<br>5-2020 -> 01-05-2020<br>2020 -> 01-01-2020	___ ___ ___ *(dd-mm-yyyy)*
4.2.4	***If 'ipsilateral TOS/UEDVT' is equal to '1' answer this question:***<br>Prior therapy TOS/UEDVT	☐ Compression therapy<br>☐ Physical therapy<br>☐ Oral antithrombotic therapy<br>☐ Local injection (botox/steroids etc)<br>☐ Thrombolysis<br>☐ Thrombosuction<br>☐ Angioplasty<br>☐ Venoplasty<br>☐ Stent placement<br>☐ Surgery<br>☐ Other<br>☐ No treatment
4.2.4.1	***If 'Prior therapy TOS/UEDVT' is equal to 'Other' answer this question:***<br>Please specify other therapy	
4.2.4.2	***If 'Prior therapy TOS/UEDVT' is equal to 'Oral antithrombotic therapy' answer this question:***<br>Which antithrombotic?	☐ Acetylsalicylic acid<br>☐ ADP inhibitor (e.g. clopidogrel)<br>☐ Vitamin K antagonist (e.g. acenocoumarol)<br>☐ Directly acting oral anticoagulants (DOACs)<br>☐ Low molecular weight heparin<br>☐ Other
4.2.4.3	***If 'Prior therapy TOS/UEDVT' is equal to 'Oral antithrombotic therapy' answer this question:***<br>Is patient still on antithrombotics?	○ Yes<br>○ No
4.2.4.3.1	***If 'Is patient still on antithrombotics?' is equal to 'No' answer this question:***<br>Duration of antithrombotic use	○ 3 months<br>○ 6 months<br>○ 1 year<br>○ other

4.2.4.3.1.1	***If 'Duration of antithrombotic use' is equal to 'other' answer this question:***<br>Specify duration of antithrombotic use	
4.2.4.4	***If 'Prior therapy TOS/UEDVT' is equal to 'Local injection (botox/steroids etc)' answer this question:***<br>Date local injection<br>If precise date is unknown:<br>5-2020 -> 01-05-2020<br>2020 -> 01-01-2020	*(dd-mm-yyyy)*
4.2.4.5	***If 'Prior therapy TOS/UEDVT' is equal to 'Local injection (botox/steroids etc)' answer this question:***<br>Which agent was injected	◯ Botulinium toxin<br>◯ Steroids<br>◯ Local anestheticum<br>◯ Other<br>◯ Unknown
4.2.4.5.1	***If 'Which agent was injected' is equal to 'Other' answer this question:***<br>Which ' other' agent	
4.2.4.6	***If 'Prior therapy TOS/UEDVT' is equal to 'Local injection (botox/steroids etc)' answer this question:***<br>Which structure was injected?	☐ Anterior scalene muscle<br>☐ Middle scalene muscle<br>☐ Subclavius muscle<br>☐ Pectoralis minor<br>☐ Other<br>☐ Unknown
4.2.4.7	***If 'Prior therapy TOS/UEDVT' is equal to 'Local injection (botox/steroids etc)' answer this question:***<br>Which technique was used?	☐ Landmarks<br>☐ EMG guidance<br>☐ Fluoroscopic guidande<br>☐ Ultrasound<br>☐ CT<br>☐ MRI<br>☐ Other<br>☐ Unknown
4.2.4.7.1	***If 'Which technique was used?' is equal to 'Other' answer this question:***<br>Which other technique	

4.2.4.8	***If 'Prior therapy TOS/UEDVT' is equal to 'Local injection (botox/steroids etc)' answer this question:***<br>Symptom relief after injection?	◯ Yes<br>◯ Partial<br>◯ No
4.2.4.8.1	***If 'Symptom relief after injection?' is not equal to 'No' answer this question:***<br>Duration of symptom relief	
4.2.4.9	***If 'Prior therapy TOS/UEDVT' is equal to 'Stent placement' answer this question:***<br>Date stent was placed<br>If precise date is unknown:<br>5-2020 -> 01-05-2020<br>2020 -> 01-01-2020	*(dd-mm-yyyy)*
4.2.4.10	***If 'Prior therapy TOS/UEDVT' is equal to 'Stent placement' answer this question:***<br>Location of stent	☐ Subclavian vein<br>☐ Axillary vein<br>☐ Brachiocephalic vein<br>☐ Subclavian artery<br>☐ Axillary artery
4.2.4.11	***If 'Prior therapy TOS/UEDVT' is equal to 'Stent placement' answer this question:***<br>Type and brand of stent used	
4.2.4.12	***If 'Prior therapy TOS/UEDVT' is equal to 'Stent placement' answer this question:***<br>Total length of stent(s)	cm
4.2.4.13	***If 'Prior therapy TOS/UEDVT' is equal to 'Surgery' answer this question:***<br>Date surgery<br>If precise date is unknown:<br>5-2020 -> 01-05-2020<br>2020 -> 01-01-2020	*(dd-mm-yyyy)*

4.2.4.14	***If 'Prior therapy TOS/UEDVT' is equal to 'Surgery' answer this question:*** Surgical approach	○ Transaxillary<br>○ Infraclavicular<br>○ Paraclavicular<br>○ Supraclavicular<br>○ Posterior<br>○ Claviculectomy<br>○ Clavicular rotation<br>○ Transthoracic<br>○ Other
4.2.4.15	***If 'Prior therapy TOS/UEDVT' is equal to 'Surgery' answer this question:*** Performed surgery	☐ Anterior first rib resection<br>☐ Posterior first rib resection<br>☐ Total first rib resection<br>☐ Cervical rib resection<br>☐ Second rib resection<br>☐ Partial anterior scalenectomy<br>☐ Total anterior scalenectomy<br>☐ Partial middle scalenectomy<br>☐ Total middle scalenectomy<br>☐ Partial subclavius resection<br>☐ Total subclavius resection<br>☐ Pectoralis minor division<br>☐ Partial resection of pectoralis minor<br>☐ Brachial plexus neurolysis<br>☐ Subclavian vein venolysis<br>☐ Venous reconstruction<br>☐ Embolectomy<br>☐ Arterial repair/replacement<br>☐ Arterial bypass<br>☐ Fasciotomy<br>☐ Amputation<br>☐ Other
4.2.4.15.1	***If 'Performed surgery' is equal to 'Venous reconstruction' answer this question:*** Surgical venous reconstruction	☐ Repair and primary closure<br>☐ Repair, vein patch<br>☐ Repair, prosthetic patch<br>☐ Interposition, venous<br>☐ Interposition, prosthetic<br>☐ Jugular turndown<br>☐ Other
4.2.4.15.1.1	***If 'Surgical venous reconstruction' is equal to 'Other' answer this question:*** Which 'other' venous reconstruction technique	

4.2.4.15.2	***If 'Performed surgery' is equal to 'Embolectomy' answer this question:***<br>Embolectomy peformed of	☐ Subclavian artery<br>☐ Axillary artery<br>☐ Brachial artery<br>☐ Radial artery<br>☐ Ulnar artery
4.2.4.15.3	***If 'Performed surgery' is equal to 'Arterial repair/replacement' answer this question:***<br>Which artery was repaired/replaced?	☐ Subclavian artery<br>☐ Axillary artery<br>☐ Brachial artery<br>☐ Radial artery<br>☐ Ulnar artery
4.2.4.15.4	***If 'Performed surgery' is equal to 'Arterial repair/replacement' answer this question:***<br>Material used	○ Primary closure<br>○ Saphenous vein<br>○ PTFE<br>○ Dacron<br>○ Other
4.2.4.15.4.1	***If 'Material used' is equal to 'Other' answer this question:***<br>Other material	
4.2.4.15.5	***If 'Performed surgery' is equal to 'Arterial bypass' answer this question:***<br>Inflow vessel used for bypass	☐ Subclavian artery, ipsilateral<br>☐ Axillary artery, ipsilateral<br>☐ Brachial artery, ipsilateral<br>☐ Subclavian artery, contralateral<br>☐ Axillary artery, contralateral<br>☐ Carotid artery, ipsilateral<br>☐ Other
4.2.4.15.6	***If 'Performed surgery' is equal to 'Arterial bypass' answer this question:***<br>Outflow vessel used for bypass	☐ Subclavian artery<br>☐ Axillary artery<br>☐ Brachial artery<br>☐ Radial artery<br>☐ Ulnar artery
4.2.4.15.7	***If 'Performed surgery' is equal to 'Arterial bypass' answer this question:***<br>Material used	○ Venous<br>○ PTFE<br>○ Dacron<br>○ Other

4.2.4.15.8	***If 'Performed surgery' is equal to 'Fasciotomy' answer this question:***<br>Fasciotomy performed of	☐ Hand<br>☐ Forearm<br>☐ Upperarm
4.2.4.15.9	***If 'Performed surgery' is equal to 'Amputation' answer this question:***<br>Amputation performed of<br>Please choose the most proximal level of amputation.	☐ Dig 1<br>☐ Dig 2<br>☐ Dig 3<br>☐ Dig 4<br>☐ Dig 5<br>☐ Hand<br>☐ Forearm<br>☐ Upperarm
4.2.4.15.10	***If 'Performed surgery' is equal to 'Other' answer this question:***<br>Please describe other surgical interventions	
4.3	History of smoking?	○ Current smoker<br>○ History of smoking<br>○ Never smoked<br>○ Unknown
4.4	***If 'calculation, a/vtos/puedvt + female' is equal to '1' answer this question:***<br>Recent/current pregnancy or childbirth?	○ Yes<br>○ No<br>○ Unknown
4.5	ASA grade	○ 1<br>○ 2<br>○ 3<br>○ 4<br>○ 5

5. Baseline - Family history

Number	Question	Answers

5.1	***If 'Calculation, vTOS+pUEDVT+aTOS' is equal to '1' answer this question:***<br>Family history of known hypercoagulable state	○ Yes<br>○ No<br>○ Unknown
5.1.1	***If 'Family history of known hypercoagulable state' is equal to 'Yes' answer this question:***<br>Which hypercoagulable state?	☐ Factor V Leiden<br>☐ Prothrombin G20210A<br>☐ High factor VIII<br>☐ Factor VII mutation<br>☐ Factor II mutation<br>☐ Protein C deficiency<br>☐ Protein S deficiency<br>☐ Antithrombin deficiency<br>☐ Antiphospholipid antibodies<br>☐ Lupus anticoagulant<br>☐ Anticardiolipin IgG<br>☐ Anticardiolipin IgM<br>☐ Anti-beta-2 glycoproteine IgG<br>☐ Anti-beta-2 glycoproteine IgM<br>☐ Plasminogeen activator inhibitor-1<br>☐ Other
5.2	***If 'calculation, vTOS+pUEDVT' is equal to '1' answer this question:***<br>Family history of venous thrombosis?	○ Yes<br>○ No<br>○ Unknown
5.2.1	***If 'Family history of venous thrombosis?' is equal to 'Yes' answer this question:***<br>Which type of thormbosis?	☐ Lower extremity DVT<br>☐ Pulmonary embolus<br>☐ Upper extermity DVT<br>☐ Other
5.2.1.1	***If 'Which type of thormbosis?' is equal to 'Other' answer this question:***<br>Specify other type of DVT	
5.3	Family history of TOS?	○ Yes<br>○ No<br>○ Unknown
5.3.1	***If 'Family history of TOS?' is equal to 'Yes' answer this question:***<br>Specify family history of TOS, including treatment performed.	

6. Presentation - Presentation

Number	Question	Answers
6.1	Was patient referred to your hospital for (additional) treatment?<br>If diagnosis was made in another hospital and patient was referred to your hospital for (additional) treatment, please choose yes.<br><br>Examples: Diagnosis of nTOS in another hospital, referred to your hospital for decompression surgery.<br><br>Diagnosis of thrombus in subclavian vein in another hospital, started with anticoagulants and referred to your hospital for analysis.<br><br>Presentation with acute ischemia due to aTOS treated with thrombolysis in another hospital, referred to your hospital for additional decompression surgery.	◯ Yes<br>◯ No
6.2	Date onset of symptoms<br>If precise date is unknown:<br><br>5-2020 -> 01-05-2020<br><br>2020 -> 01-01-2020	[ ] [ ] [ ] *(dd-mm-yyyy)*
6.1.1	***If 'Was patient referred to your hospital for (additional) treatment?' is equal to 'No' answer this question:***<br>Date first presentation in hospital<br>If precise date is unknown:<br><br>5-2020 -> 01-05-2020<br><br>2020 -> 01-01-2020	[ ] [ ] [ ] *(dd-mm-yyyy)*
6.1.2	***If 'Was patient referred to your hospital for (additional) treatment?' is equal to 'Yes' answer this question:***<br>Date first presentation in referring hospital<br>If precise date is unknown:<br><br>5-2020 -> 01-05-2020<br><br>2020 -> 01-01-2020	[ ] [ ] [ ] *(dd-mm-yyyy)*
6.1.3	***If 'Was patient referred to your hospital for (additional) treatment?' is equal to 'Yes' answer this question:***<br>Date first presentation in your hospital<br>If precise date is unknown:<br><br>5-2020 -> 01-05-2020<br><br>2020 -> 01-01-2020	[ ] [ ] [ ] *(dd-mm-yyyy)*
6.3	Affected side	◯ Left<br>◯ Right

6.4	Symptoms during initial presentation<br>This concerns the symptoms during the initial presentation, before start of treatment. So if patient is referred, please provide the symptoms during initial presentation. Please choose 'unknown' if the symptoms during initial presentation are unknown/unclear.	☐ Pain<br>☐ Swelling<br>☐ Tingling<br>☐ Numbness<br>☐ Heavyness<br>☐ Loss of function<br>☐ Loss of strength/weakness<br>☐ Discoloration-white<br>☐ Discoloration-red<br>☐ Discoloration-blue<br>☐ Coldness<br>☐ Temperature sensitivity<br>☐ Vein collaterals<br>☐ Ulceration, gangrene or tissue loss<br>☐ Microemboli<br>☐ Unknown<br>☐ Other
6.4.1	***If 'Symptoms during initial presentation' is equal to 'Other' answer this question:***<br>Specify other symptoms	
6.4.2	***If 'Symptoms during initial presentation' is equal to 'Pain' answer this question:***<br>Location pain	☐ Neck<br>☐ Chest<br>☐ Shoulder<br>☐ Whole arm<br>☐ Upper arm<br>☐ Forearm<br>☐ Hand, without all digiti<br>☐ Hand, including all digiti<br>☐ Specific dig(iti)<br>☐ Unclear
6.4.2.1	***If 'Location pain' is equal to 'Specific dig(iti)' answer this question:***<br>Pain in which specific dig(iti)?	☐ Dig 1<br>☐ Dig 2<br>☐ Dig 3<br>☐ Dig 4<br>☐ Dig 5

6.4.3	***If 'Symptoms during initial presentation' is equal to 'Tingling' answer this question:***<br>Location tingling	☐ Neck<br>☐ Chest<br>☐ Shoulder<br>☐ Upper arm<br>☐ Forearm<br>☐ Hand, dig 1-3<br>☐ Hand, dig 4-5<br>☐ Unclear
6.4.4	***If 'Symptoms during initial presentation' is equal to 'Numbness' answer this question:***<br>Location numbness	☐ Neck<br>☐ Chest<br>☐ Shoulder<br>☐ Upper arm<br>☐ Forearm<br>☐ Hand, dig 1-3<br>☐ Hand, dig 4-5<br>☐ Unclear
6.4.5	***If 'Symptoms during initial presentation' is equal to 'Ulceration, gangrene or tissue loss' answer this question:***<br>Location ulcerations	☐ Neck<br>☐ Chest<br>☐ Shoulder<br>☐ Upper arm<br>☐ Forearm<br>☐ Hand, dig 1-3<br>☐ Hand, dig 4-5<br>☐ Unclear
6.4.6	***If 'Symptoms during initial presentation' is equal to 'Microemboli' answer this question:***<br>Please describe microemboli	
6.5	Nature of complaints	☐ Continuously<br>☐ Provoked by excercise<br>☐ Provoked by work<br>☐ Posture dependant<br>☐ Nocturnal<br>☐ Unclear
6.5.1	***If 'Nature of complaints' is equal to 'Provoked by excercise' answer this question:***<br>Specify excercise	

6.5.2	***If 'Nature of complaints' is equal to 'Provoked by work' answer this question:***<br>Able to work?	◯ Yes<br>◯ No<br>◯ Partially<br>◯ Unknown
6.5.3	***If 'Nature of complaints' is equal to 'Posture dependant' answer this question:***<br>Specify posture	
6.6	Acute or gradual onset of symptoms?	◯ Acute<br>◯ Gradual<br>◯ Gradual with acute worsening<br>◯ Unclear
6.7	Calc, acute onset	
6.7.1	***If 'Calc, acute onset' is equal to '1' answer this question:***<br>Any (unusual) activities leading to start of symptoms?	
6.8	Any (unusual) activities in the days or weeks before start of symptoms potentially connected?	
6.9	***If 'calculation, nTOS' is equal to '1' answer this question:***<br>Anything that has a positive effect on complaints?	
6.10	***If 'calculation, nTOS' is equal to '1' answer this question:***<br>Symptoms are generally	◯ Getting worse<br>◯ Getting beter<br>◯ Recently unchanged<br>◯ Unknown
6.11	***If 'calculation, vTOS+pUEDVT' is equal to '1' answer this question:***<br>Recent prolonged immobilization (e.g. flights, prolonged immobilisation after surgery etc.)	◯ Yes<br>◯ No<br>◯ Unknown

7. Presentation - Medication use

Number	Question	Answers
7.1	Medication use at the time of first presentation?	◯ Yes<br>◯ No
7.1.1	***If 'Medication use at the time of first presentation?' is equal to 'Yes' answer this question:***<br>Antithrombotic use?	◯ Yes<br>◯ No
7.1.1.1	***If 'Antithrombotic use?' is equal to 'Yes' answer this question:***<br>Which antithrombotic?	☐ Acetylsalicylic acid<br>☐ ADP inhibitor (e.g. clopidogrel)<br>☐ Vitamin K antagonist (e.g. acenocoumarol)<br>☐ Directly acting oral anticoagulants (DOACs)<br>☐ Low molecular weight heparin<br>☐ Other
7.1.1.1.1	***If 'Which antithrombotic?' is equal to 'Other' answer this question:***<br>Please describe other antithrombotic	
7.2	calculation, med use + nTOS	
7.2.1	***If 'calculation, med use + nTOS' is equal to '1' answer this question:***<br>Analgetica use specific for TOS symptoms	☐ No<br>☐ Paracetamol<br>☐ NSAIDS<br>☐ Opioids
7.3	***If 'calculation, a/vtos/puedvt + female' is equal to '1' answer this question:***<br>Use of estrogen containing contraceptive at the time of first presentation?	◯ Yes<br>◯ No<br>◯ Unknown
7.1.2	***If 'Medication use at the time of first presentation?' is equal to 'Yes' answer this question:***<br>Please specify medication use (besides the medication mentioned above)	

8. Presentation - Physical exam

Number	Question	Answers
8.1	Physical exam performed?	◯ Yes<br>◯ No<br>◯ Unknown
8.1.1	***If 'Physical exam performed?' is equal to 'Yes' answer this question:***<br>What was tested/examined?	☐ Inspection affected upper extremity<br>☐ Posture<br>☐ Arm use<br>☐ nTOS testing (e.g. palpation supraclavicular/pec minor space)<br>☐ Neurological exam<br>☐ Vascular exam (pulsations, blood pressure, etc)<br>☐ Specific TOS tests (e.g. Roos test, military brace)<br>☐ Contralateral upper extremity<br>☐ Other tests/examinations or additional remarks
8.1.1.1	***If 'What was tested/examined?' is equal to 'Inspection affected upper extremity' answer this question:***<br>Inspection affected upper extremity	☐ No abnormalities<br>☐ Swelling<br>☐ Athrophy<br>☐ Visible chest wall collaterals<br>☐ Discoloration white<br>☐ Discoloration red<br>☐ Discoloration blue<br>☐ Decreased temperature<br>☐ Microembolisation<br>☐ Ulcerations, gangrene or tissue loss<br>☐ Other
8.1.1.1.1	***If 'Inspection affected upper extremity' is equal to 'Other' answer this question:***<br>inspection, other	
8.2	Calc, discoloration	

8.2.1	***If 'Calc, discoloration' is equal to '1' answer this question:***<br>Discoloration of	☐ Neck<br>☐ Chest<br>☐ Shoulder<br>☐ Whole arm<br>☐ Upper arm<br>☐ Forearm<br>☐ Hand, without all digiti<br>☐ Hand, including all digiti<br>☐ Specific dig(iti)<br>☐ Unclear
8.2.1.1	***If 'Discoloration of' is equal to 'Specific dig(iti)' answer this question:***<br>Which dig(iti)	☐ Dig 1<br>☐ Dig 2<br>☐ Dig 3<br>☐ Dig 4<br>☐ Dig 5
8.1.1.1.2	***If 'Inspection affected upper extremity' is equal to 'Athrophy' answer this question:***<br>Atrophy	☐ Shoulder<br>☐ Neck<br>☐ Chest<br>☐ Upper arm<br>☐ Forearm<br>☐ Hand, dig 1-3<br>☐ Hand, dig 4-5
8.1.1.1.3	***If 'Inspection affected upper extremity' is equal to 'Microembolisation' answer this question:***<br>Please describe microemboli	
8.1.1.1.4	***If 'Inspection affected upper extremity' is equal to 'Ulcerations, gangrene or tissue loss' answer this question:***<br>Ulcerations, gangrene or tissue loss of	☐ Dig 1<br>☐ Dig 2<br>☐ Dig 3<br>☐ Dig 4<br>☐ Dig 5<br>☐ Hand<br>☐ Forearm<br>☐ Upperarm

8.1.1.2	***If 'What was tested/examined?' is equal to 'Posture' answer this question:***<br>Posture<br>Slumped spinal posture is a term that describes a slumped flexion posture of the spine. It involves excessive posterior tilt of the pelvis, decreased (hypo) lordosis of the lumbar spine, excessive (hyper) kyphosis of the thoracic spine, hypolordosis of the lower cervical spine, hyperlordosis of the upper cervical spine, and a protracted head.	○ Normal<br>○ Slumped
8.1.1.3	***If 'What was tested/examined?' is equal to 'Arm use' answer this question:***<br>Arm use	○ Normal<br>○ Favors contralateral arm/restricts use of ipsilateral arm
8.1.1.4	***If 'What was tested/examined?' is equal to 'nTOS testing (e.g. palpation supraclavicular/pec minor space)' answer this question:***<br>nTOS testing performed? (e.g. palpation supraclavicular/pec minor space)	○ Yes<br>○ No
8.1.1.4.1	***If 'nTOS testing performed? (e.g. palpation supraclavicular/pec minor space)' is equal to 'Yes' answer this question:***<br>Neck exam	☐ Normal<br>☐ Swelling<br>☐ Tightness<br>☐ Asymetry<br>☐ Possible cervical rib<br>☐ Not performed
8.1.1.4.2	***If 'nTOS testing performed? (e.g. palpation supraclavicular/pec minor space)' is equal to 'Yes' answer this question:***<br>Supraclavicular tenderness with palpation	☐ Nontender<br>☐ Mild<br>☐ Moderate<br>☐ Severe<br>☐ Reproduces distal symptoms<br>☐ Not performed
8.1.1.4.3	***If 'nTOS testing performed? (e.g. palpation supraclavicular/pec minor space)' is equal to 'Yes' answer this question:***<br>Pectoralis minor space tenderness with palpation	☐ Nontender<br>☐ Mild<br>☐ Moderate<br>☐ Severe<br>☐ Reproduces distal symptoms<br>☐ Not performed
8.1.1.4.4	***If 'nTOS testing performed? (e.g. palpation supraclavicular/pec minor space)' is equal to 'Yes' answer this question:***<br>Axillary tenderness with palpation	☐ Nontender<br>☐ Mild<br>☐ Moderate<br>☐ Severe<br>☐ Reproduces distal symptoms<br>☐ Not performed

8.1.1.5	***If 'What was tested/examined?' is equal to 'Neurological exam' answer this question:*** Sensiblity, general	○ Normal<br>○ Decreased sensibility<br>○ Absent sensibility<br>○ Not tested
8.1.1.6	***If 'What was tested/examined?' is equal to 'Neurological exam' answer this question:*** Muscle strength, general	○ Normal<br>○ Decreased strength, mild<br>○ Decreased strength, severe<br>○ Absent contractions<br>○ Not tested
8.1.1.7	***If 'What was tested/examined?' is equal to 'Neurological exam' answer this question:*** Specific nerves tested?	☐ No<br>☐ Median nerve<br>☐ Ulnar nerve<br>☐ radial nerve
8.1.1.7.1	***If 'Specific nerves tested?' is equal to 'Median nerve' answer this question:*** Median nerve	☐ Normal sensibility and strength<br>☐ Normal sensibility<br>☐ Decresed sensibility<br>☐ Absent sensibility<br>☐ Normal strength<br>☐ Descreased strength, mild<br>☐ Descreased strength, severe<br>☐ No muscle contractions
8.1.1.7.2	***If 'Specific nerves tested?' is equal to 'Ulnar nerve' answer this question:*** Ulnar nerve	☐ Normal sensibility and strength<br>☐ Normal sensibility<br>☐ Decresed sensibility<br>☐ Absent sensibility<br>☐ Normal strength<br>☐ Descreased strength, mild<br>☐ Descreased strength, severe<br>☐ No muscle contractions
8.1.1.7.3	***If 'Specific nerves tested?' is equal to 'radial nerve' answer this question:*** Radial nerve	☐ Normal sensibility and strength<br>☐ Normal sensibility<br>☐ Decresed sensibility<br>☐ Absent sensibility<br>☐ Normal strength<br>☐ Descreased strength, mild<br>☐ Descreased strength, severe<br>☐ No muscle contractions

8.1.1.8	***If 'What was tested/examined?' is equal to 'Neurological exam' answer this question:***<br>Tinel's sign present?	◯ Yes<br>◯ No<br>◯ Not tested
8.1.1.8.1	***If 'Tinel's sign present?' is equal to 'Yes' answer this question:***<br>Tinel's sign positive at	☐ Wrist<br>☐ Cubital<br>☐ Plexus
8.1.1.9	***If 'What was tested/examined?' is equal to 'Vascular exam (pulsations, blood pressure, etc)' answer this question:***<br>Which arteries were examined?	☐ Subclavian artery<br>☐ Axillary artery<br>☐ Brachial artery<br>☐ Radial artery<br>☐ Ulnar artery<br>☐ None
8.1.1.9.1	***If 'Which arteries were examined?' is equal to 'Subclavian artery' answer this question:***<br>Subclavian artery	☐ Normal pulsations<br>☐ Weak pulsations<br>☐ Dopler signals only<br>☐ Absent pulsations<br>☐ Palpabel pulsating mass present (aneurysm?)<br>☐ Bruit/thrill present
8.1.1.9.2	***If 'Which arteries were examined?' is equal to 'Axillary artery' answer this question:***<br>Axillary artery	☐ Normal pulsations<br>☐ Weak pulsations<br>☐ Dopler signals only<br>☐ Absent pulsations<br>☐ Palpabel pulsating mass present (aneurysm?)<br>☐ Bruit/thrill present
8.1.1.9.3	***If 'Which arteries were examined?' is equal to 'Brachial artery' answer this question:***<br>Brachial artery	☐ Normal pulsations<br>☐ Weak pulsations<br>☐ Dopler signals only<br>☐ Absent pulsations<br>☐ Palpabel pulsating mass present (aneurysm?)<br>☐ Bruit/thrill present

8.1.1.9.4	***If 'Which arteries were examined?' is equal to 'Radial artery' answer this question:***<br>Radial artery	☐ Normal pulsations<br>☐ Weak pulsations<br>☐ Dopler signals only<br>☐ Absent pulsations<br>☐ Palpabel pulsating mass present (aneurysm?)<br>☐ Bruit/thrill present
8.1.1.9.5	***If 'Which arteries were examined?' is equal to 'Ulnar artery' answer this question:***<br>Ulnar artery	☐ Normal pulsations<br>☐ Weak pulsations<br>☐ Dopler signals only<br>☐ Absent pulsations<br>☐ Palpabel pulsating mass present (aneurysm?)<br>☐ Bruit/thrill present
8.1.1.10	***If 'What was tested/examined?' is equal to 'Vascular exam (pulsations, blood pressure, etc)' answer this question:***<br>Blood pressure difference present between both upper extremities	○ Yes, BP of affected arm is lower<br>○ Yes, BP of affected arm is higher<br>○ No<br>○ Not tested
8.1.1.10.1	***If 'Blood pressure difference present between both upper extremities' is equal to 'Yes, BP of affected arm is lower' answer this question:***<br>Systolic blood pressure difference	[ ] mmHG
8.1.1.11	***If 'What was tested/examined?' is equal to 'Vascular exam (pulsations, blood pressure, etc)' answer this question:***<br>Capillary refill	○ Not tested<br>○ Normal<br>○ Prolonged, <5sec<br>○ Prolonged >5sec
8.1.1.12	***If 'What was tested/examined?' is equal to 'Specific TOS tests (e.g. Roos test, military brace)' answer this question:***<br>Which TOS tests?	☐ Wright's test<br>☐ Adson's test<br>☐ Costoclavicular / Military brace / Eden's test<br>☐ Upper Limb Tension Tests (ULTT's) / Elvey test<br>☐ Roos test / Elevated arm stress test
8.1.1.12.1	***If 'Which TOS tests?' is equal to 'Wright's test' answer this question:***<br>Wright's test	○ Negative<br>○ Positive, loss of radial pulse<br>○ Positive, reproduction of patients symptoms<br>○ Positive, loss of radial pulse AND reproduction of patients symptoms

8.1.1.12.2	***If 'Which TOS tests?' is equal to 'Adson's test' answer this question:***<br>Adson's test	◯ Negative<br>◯ Positive, marked decrease of radial pulse<br>◯ Positive, loss of radial pulse
8.1.1.12.3	***If 'Which TOS tests?' is equal to 'Costoclavicular / Military brace / Eden's test' answer this question:***<br>Costoclavicular / Military Brace / Eden's Test	◯ Negative<br>◯ Positive, marked decrease of radial pulse<br>◯ Positive, loss of radial pulse<br>◯ Positive, reproduction of patients symptoms<br>◯ Positive, decrease/loss of radial pulse AND reproduction of patients symptoms
8.1.1.12.4	***If 'Which TOS tests?' is equal to 'Upper Limb Tension Tests (ULTT's) / Elvey test' answer this question:***<br>Upper Limb Tension Tests (ULTT's) / Elvey test	◯ Negative<br>◯ Positive, reproduction of patients symptoms
8.1.1.12.5	***If 'Which TOS tests?' is equal to 'Roos test / Elevated arm stress test' answer this question:***<br>Roos test / elevated arm stress test	◯ Negative<br>◯ Positive, reproduction of patients symptoms<br>◯ Positive, not able to complete test
8.3	calc roos positive	
8.3.1	***If 'calc roos positive' is equal to '1' answer this question:***<br>First symptoms after	[ ] seconds
8.1.1.12.5.1	***If 'Roos test / elevated arm stress test' is equal to 'Positive, not able to complete test' answer this question:***<br>Cannot continue after	[ ] seconds
8.1.1.13	***If 'What was tested/examined?' is equal to 'Contralateral upper extremity' answer this question:***<br>Contralateral upper extremity exam normal	◯ Yes<br>◯ No
8.1.1.13.1	***If 'Contralateral upper extremity exam normal' is equal to 'No' answer this question:***<br>Please describe abnormalities	[ ]
8.1.1.14	***If 'What was tested/examined?' is equal to 'Other tests/examinations or additional remarks' answer this question:***<br>Please describe any other tests/examinations or any remarks on the physical exam in detail	[ ]

9. Presentation - Upper extremity PTS score

Number	Question	Answers
9.1	Was the upper extremity post thrombotic syndrome score used to evaluate the patient?<br>See ..... (link) for the article	◯ Yes<br>◯ No
9.1.1	***If 'Was the upper extremity post thrombotic syndrome score used to evaluate the patient?' is equal to 'Yes' answer this question:***<br>Date of assessment	[ ] [ ] [ ] *(dd-mm-yyyy)*
	**Symptoms of post thrombotic syndrome**	
9.1.3	***If 'Was the upper extremity post thrombotic syndrome score used to evaluate the patient?' is equal to 'Yes' answer this question:***<br>Edema/swelling of the arm	◯ Absent (0 points)<br>◯ Mild (1 point)<br>◯ Moderate (2 points)<br>◯ Severe (3 points)
9.1.4	***If 'Was the upper extremity post thrombotic syndrome score used to evaluate the patient?' is equal to 'Yes' answer this question:***<br>Heavy feeling of the arm	◯ Absent (0 points)<br>◯ Mild (1 point)<br>◯ Moderate (2 points)<br>◯ Severe (3 points)
9.1.5	***If 'Was the upper extremity post thrombotic syndrome score used to evaluate the patient?' is equal to 'Yes' answer this question:***<br>Fatigue on using arm	◯ Absent (0 points)<br>◯ Mild (1 point)<br>◯ Moderate (2 points)<br>◯ Severe (3 points)
9.1.6	***If 'Was the upper extremity post thrombotic syndrome score used to evaluate the patient?' is equal to 'Yes' answer this question:***<br>Pain (chronic or during specific excercise)	◯ Absent (0 points)<br>◯ Mild (1 point)<br>◯ Moderate (2 points)<br>◯ Severe (3 points)
9.1.7	***If 'Was the upper extremity post thrombotic syndrome score used to evaluate the patient?' is equal to 'Yes' answer this question:***<br>Functional limitations arm	◯ Absent (0 points)<br>◯ Mild (1 point)<br>◯ Moderate (2 points)<br>◯ Severe (3 points)
	**Clinical signs of post thrombotic syndrome**	
9.1.9	***If 'Was the upper extremity post thrombotic syndrome score used to evaluate the patient?' is equal to 'Yes' answer this question:***<br>Swelling arm measured by circumference of upper and lower arm versus contralateral side	◯ Absent (0 points)<br>◯ Mild (1 point)<br>◯ Moderate (2 points)<br>◯ Severe (3 points)

9.1.10	***If 'Was the upper extremity post thrombotic syndrome score used to evaluate the patient?' is equal to 'Yes' answer this question:***<br>Discoloration of arm/hand/fingers (red/white/cyanotic) in rest	◯ Absent (0 points)<br>◯ Mild (1 point)<br>◯ Moderate (2 points)<br>◯ Severe (3 points)
9.1.11	***If 'Was the upper extremity post thrombotic syndrome score used to evaluate the patient?' is equal to 'Yes' answer this question:***<br>Collateralization or collateral veins around shoulder/torso/breast	◯ Absent (0 points)<br>◯ Mild (1 point)<br>◯ Moderate (2 points)<br>◯ Severe (3 points)
9.1.12	***If 'Was the upper extremity post thrombotic syndrome score used to evaluate the patient?' is equal to 'Yes' answer this question:***<br>Total signs and symptoms score (max 24)	

10. Presentation - Lab reports

Number	Question	Answers
10.1	Laboratory testing performed?	◯ Yes<br>◯ No
10.1.1	***If 'Laboratory testing performed?' is equal to 'Yes' answer this question:***<br>Which laboratory test(s)	☐ D-dimer<br>☐ Creatinine<br>☐ Hypercoagulability testing<br>☐ VerifyNow assay<br>☐ CYP2C19 genetic testing<br>☐ Other
10.1.1.1	***If 'Which laboratory test(s)' is equal to 'D-dimer' answer this question:***<br>Date d-dimer tested	[ ] [ ] [ ] *(dd-mm-yyyy)*
10.1.1.2	***If 'Which laboratory test(s)' is equal to 'D-dimer' answer this question:***<br>D-dimer level (in mg/L)	[ ] mg/L

10.1.1.3	***If 'Which laboratory test(s)' is equal to 'Creatinine' answer this question:***<br>Creatinine level, reported in µmol/l<br>To convert mg/dl to µmol/l, multiply by 88,4 . Please round up the outcome to the nearest whole number.<br><br>Example: Creatinine level 0.90 mg/dl<br><br>0.90 x 88,4 = 79,56<br><br>Creatinine level 80 mg/dl	[ ] µmol/l
10.1.1.4	***If 'Which laboratory test(s)' is equal to 'Hypercoagulability testing' answer this question:***<br>Which hypercoagulability tests performed?	☐ Prothrombin time (PT)<br>☐ Activated partial thromboplastin time (aPTT)<br>☐ Fibronogen level<br>☐ Anticardiolipin IgG<br>☐ Anticardiolipin IgM<br>☐ Beta-2 glycoprotein I IgG<br>☐ Beta-2 glycoprotein I IgM<br>☐ Lupus anticoagulans<br>☐ Functional protein C assay<br>☐ Antigenic protein C assay<br>☐ Functional protein S assay<br>☐ Antigenic protein S assay<br>☐ Functional antithrombin assay<br>☐ Antigenic antithrombin assay<br>☐ Activated protein C resistance testing<br>☐ Factor V Leiden mutation<br>☐ Prothrombin G20210A gene mutation<br>☐ Other test(s)
10.1.1.4.1	***If 'Which hypercoagulability tests performed?' is equal to 'Prothrombin time (PT)' answer this question:***<br>Prothrombin time (PT)	[ ] Seconds
10.1.1.4.2	***If 'Which hypercoagulability tests performed?' is equal to 'Activated partial thromboplastin time (aPTT)' answer this question:***<br>Activated partial thromboplastin time (aPTT)	[ ] Seconds
10.1.1.4.3	***If 'Which hypercoagulability tests performed?' is equal to 'Fibronogen level' answer this question:***<br>Fibronogen level	[ ] g/L
10.1.1.4.4	***If 'Which hypercoagulability tests performed?' is equal to 'Anticardiolipin IgG' answer this question:***<br>Anticardiolipin IgG	○ Positive<br>○ Negative
10.1.1.4.5	***If 'Which hypercoagulability tests performed?' is equal to 'Anticardiolipin IgM' answer this question:***<br>Anticardiolipin IgM	○ Positive<br>○ Negative

10.1.1.4.6	***If 'Which hypercoagulability tests performed?' is equal to 'Beta-2 glycoprotein I IgG' answer this question:***<br>Beta-2 glycoprotein I IgG	○ Positive<br>○ Negative
10.1.1.4.7	***If 'Which hypercoagulability tests performed?' is equal to 'Beta-2 glycoprotein I IgM' answer this question:***<br>Beta-2 glycoprotein I IgM	○ Positive<br>○ Negative
10.1.1.4.8	***If 'Which hypercoagulability tests performed?' is equal to 'Lupus anticoagulans' answer this question:***<br>Lupus anticoagulans	○ Positive<br>○ Negative
10.1.1.4.9	***If 'Which hypercoagulability tests performed?' is equal to 'Functional protein C assay' answer this question:***<br>Functional protein C assay	○ Normal<br>○ Decreased
10.1.1.4.10	***If 'Which hypercoagulability tests performed?' is equal to 'Antigenic protein C assay' answer this question:***<br>Antigenic protein C assay	○ Normal<br>○ Protein defect type I<br>○ Protein defect type II
10.1.1.4.11	***If 'Which hypercoagulability tests performed?' is equal to 'Functional protein S assay' answer this question:***<br>Functional protein S assay	○ Normal<br>○ Decreased
10.1.1.4.12	***If 'Which hypercoagulability tests performed?' is equal to 'Antigenic protein S assay' answer this question:***<br>Antigenic protein S assay	○ Normal<br>○ Protein defect type I<br>○ Protein defect type II<br>○ Protein defect type III
10.1.1.4.13	***If 'Which hypercoagulability tests performed?' is equal to 'Functional antithrombin assay' answer this question:***<br>Functional antithrombin assay	○ Normal<br>○ Decreased
10.1.1.4.14	***If 'Which hypercoagulability tests performed?' is equal to 'Antigenic antithrombin assay' answer this question:***<br>Antigenic antithrombin assay	○ Normal<br>○ Antithrombin deficiency type I<br>○ Antithrombin deficiency type II
10.1.1.4.15	***If 'Which hypercoagulability tests performed?' is equal to 'Activated protein C resistance testing' answer this question:***<br>Activated protein C resistance testing	○ Normal<br>○ Protein C resistance
10.1.1.4.16	***If 'Which hypercoagulability tests performed?' is equal to 'Factor V Leiden mutation' answer this question:***<br>Factor V Leiden mutation	○ Normal<br>○ Factor V Leiden mutation present
10.1.1.4.17	***If 'Which hypercoagulability tests performed?' is equal to 'Prothrombin G20210A gene mutation' answer this question:***<br>Prothrombin G20210A gene mutation	○ Normal<br>○ Prothrombin G20210A gene mutation present

10.1.1.4.18	***If 'Which hypercoagulability tests performed?' is equal to 'Other test(s)' answer this question:*** Please describe other tests in detail	
10.1.1.5	***If 'Which laboratory test(s)' is equal to 'VerifyNow assay' answer this question:*** Which VerifyNow assay?	☐ VerifyNow P2Y12 assay<br>☐ VerifyNow Aspirin assay
10.1.1.5.1	***If 'Which VerifyNow assay?' is equal to 'VerifyNow P2Y12 assay' answer this question:*** VerifyNow P2Y12 assay baseline	PRU
10.1.1.5.2	***If 'Which VerifyNow assay?' is equal to 'VerifyNow P2Y12 assay' answer this question:*** VerifyNow P2Y12 assay Post-Treatment	PRU
10.1.1.5.3	***If 'Which VerifyNow assay?' is equal to 'VerifyNow Aspirin assay' answer this question:*** VerifyNow Aspirin assay baseline	ARU
10.1.1.5.4	***If 'Which VerifyNow assay?' is equal to 'VerifyNow Aspirin assay' answer this question:*** VerifyNow Aspirin assay Post-Treatment	ARU
10.1.1.6	***If 'Which laboratory test(s)' is equal to 'CYP2C19 genetic testing' answer this question:*** Outcome CYP2C19 testing	☐ Normal<br>☐ Decreased CYP2C19 activity<br>☐ Increased CYP2C19 activity
10.1.1.7	***If 'Which laboratory test(s)' is equal to 'Other' answer this question:*** Please describe 'other' laboratory test(s) in detail	

11. Presentation - Diagnostic modalities I

Number	Question	Answers

11.1	Performed diagnostic modalities	☐ Electromyography<br>☐ Finger photoplethysmography (PPG) or dedicated TOS PPG<br>☐ Local injection<br>☐ Ultrasound<br>☐ Duplex ultrasound<br>☐ Chest radiograph or cervical spine series<br>☐ CT<br>☐ MRI<br>☐ Venography (with or without IVUS)<br>☐ Arteriography (with or without IVUS)<br>☐ Other
11.1.1	***If 'Performed diagnostic modalities' is equal to 'Electromyography' answer this question:***<br>Date EMG	___ ___ ___ *(dd-mm-yyyy)*
11.1.2	***If 'Performed diagnostic modalities' is equal to 'Electromyography' answer this question:***<br>Please describe rationale to perform EMG	
11.1.3	***If 'Performed diagnostic modalities' is equal to 'Electromyography' answer this question:***<br>C8 nerve root stimulation velocity<br>If this was not investigated, please type '0'	___ m/sec
11.1.4	***If 'Performed diagnostic modalities' is equal to 'Electromyography' answer this question:***<br>Medial antebrachial cutaneous nerve sensory neural action potential (SNAP) amplitude<br>If this was nog investigated, please type '0'	___ microvolts
11.1.5	***If 'Performed diagnostic modalities' is equal to 'Electromyography' answer this question:***<br>Medial antebrachial cutaneous nerve sensory neural action potential (SNAP) latency<br>If this was not investigated, please type '0'	___ miliseconds
11.1.6	***If 'Performed diagnostic modalities' is equal to 'Electromyography' answer this question:***<br>Any other relevant EMG findings?	
11.1.7	***If 'Performed diagnostic modalities' is equal to 'Finger photoplethysmography (PPG) or dedicated TOS PPG' answer this question:***<br>Date finger photoplethysmography (PPG)	___ ___ ___ *(dd-mm-yyyy)*

11.1.8 ***If 'Performed diagnostic modalities' is equal to 'Finger photoplethysmography (PPG) or dedicated TOS PPG' answer this question:***
What sort of finger PPG was performed

- [ ] At rest
- [ ] Dedicated TOS PPG, right side
- [ ] Dedicated TOS PPG, left side

11.1.8.1 ***If 'What sort of finger PPG was performed' is equal to 'At rest' answer this question:***
finger PPG at rest

	Signal
Dig 1	---
Dig 2	---
Dig 3	---
Dig 4	---
dig 5	---

11.1.8.2 ***If 'What sort of finger PPG was performed' is equal to 'Dedicated TOS PPG, right side' answer this question:***
Dedicated TOS PPG, right side

	Signal
In rest	---
Costoclavicular maneuvre	---
Arms 90° abducted in coronal plain	---
Arms 180° abducted /Wrights position	---
Elevated arms stress test/Roos test	---
Allen test	---
Adson test	---
Arms 90° abducted in sagital plain	---
Symptomatic position	---
Other, please describe position and signal	

11.1.8.3 ***If 'What sort of finger PPG was performed' is equal to 'Dedicated TOS PPG, left side' answer this question:***
Dedicated TOS PPG, left side

	Signal
In rest	---
Costoclavicular maneuvre	---
Arms 90° abducted in coronal plain	---
Arms 180° abducted /Wrights position	---
Elevated arms stress test/Roos test	---
Allen test	---
Adson test	---
Arms 90° abducted in sagital plain	---
Symptomatic position	---
Other, please describe position and signal	

11.1.9 ***If 'Performed diagnostic modalities' is equal to 'Local injection' answer this question:***
Date local injection

___ ___ ___ *(dd-mm-yyyy)*

11.1.10 ***If 'Performed diagnostic modalities' is equal to 'Local injection' answer this question:***
Which agent was injected

- [ ] Botulinium toxin
- [ ] Steroids
- [ ] Local anestheticum
- [ ] Other
- [ ] Unknown

11.1.10.1	***If 'Which agent was injected' is equal to 'Other' answer this question:***<br>Which other agent	
11.1.11	***If 'Performed diagnostic modalities' is equal to 'Local injection' answer this question:***<br>Dosage of agent	
11.1.12	***If 'Performed diagnostic modalities' is equal to 'Local injection' answer this question:***<br>Which structure was injected?	☐ Anterior scalene muscle<br>☐ Middle scalene muscle<br>☐ Subclavius muscle<br>☐ Pectoralis minor<br>☐ Other<br>☐ Unknown
11.1.12.1	***If 'Which structure was injected?' is equal to 'Other' answer this question:***<br>Which other structure	
11.1.13	***If 'Performed diagnostic modalities' is equal to 'Local injection' answer this question:***<br>Which technique was used?	☐ Landmarks<br>☐ EMG guidance<br>☐ Fluoroscopic guidande<br>☐ Ultrasound<br>☐ CT<br>☐ MRI<br>☐ Other<br>☐ Unknown
11.1.13.1	***If 'Which technique was used?' is equal to 'Other' answer this question:***<br>Which other technique	
11.1.14	***If 'Performed diagnostic modalities' is equal to 'Local injection' answer this question:***<br>Symptom relief after injection?	○ Yes<br>○ Partial<br>○ No
11.1.14.1	***If 'Symptom relief after injection?' is not equal to 'No' answer this question:***<br>Duration of symptom relief	

11.1.15	***If 'Performed diagnostic modalities' is equal to 'Ultrasound' answer this question:*** Date ultrasound	☐ ☐ ☐ *(dd-mm-yyyy)*
11.1.16	***If 'Performed diagnostic modalities' is equal to 'Ultrasound' answer this question:*** Please describe rationale for performing ultrasound	☐
11.1.17	***If 'Performed diagnostic modalities' is equal to 'Ultrasound' answer this question:*** Ultrasound normal or abnormal	◯ Normal<br>◯ Abnormal
11.1.17.1	***If 'Ultrasound normal or abnormal' is equal to 'Abnormal' answer this question:*** Describe ultrasound findings	☐
11.1.18	***If 'Performed diagnostic modalities' is equal to 'Duplex ultrasound' answer this question:*** Date duplex ultrasound	☐ ☐ ☐ *(dd-mm-yyyy)*
11.1.19	***If 'Performed diagnostic modalities' is equal to 'Duplex ultrasound' answer this question:*** Was duplex performed with provocative maneuvres?	◯ Yes<br>◯ No
11.1.20	***If 'Performed diagnostic modalities' is equal to 'Duplex ultrasound' answer this question:*** Duplex performed	◯ Arterial<br>◯ Venous<br>◯ Arterial & venous
11.1.20.1	***If 'Duplex performed' is not equal to 'Arterial' answer this question:*** Vein patency, in rest	☐ Patent<br>☐ Stenotic<br>☐ Occluded
11.1.20.1.1	***If 'Vein patency, in rest' is equal to 'Stenotic' answer this question:*** Location stenosis	☐ Subclavian vein<br>☐ Axillary vein<br>☐ Brachial vein<br>☐ Cephalic vein<br>☐ Basilic vein<br>☐ Brachiocephalic vein
11.1.20.1.2	***If 'Vein patency, in rest' is equal to 'Occluded' answer this question:*** Location occlusion	☐ Subclavian vein<br>☐ Axillary vein<br>☐ Brachial vein<br>☐ Cephalic vein<br>☐ Basilic vein<br>☐ Brachiocephalic vein

11.2	duplex provocation & venous	
11.2.1	***If 'duplex provocation & venous' is equal to '1' answer this question:***<br>Vein compression with provocative maneuvres	○ Conform assesment in rest<br>○ Compression, 1-50% lumen reduction<br>○ Compression, 51-99% lumen reduction<br>○ Compression, not patent<br>○ Not judgeable
11.3	calc ipsilateral compression duplex	
11.3.1	***If 'calc ipsilateral compression duplex' is equal to '1' answer this question:***<br>Location compression	○ Costoclavicular junction<br>○ Pectoralis minor space<br>○ Both costoclavicular junction and pectoralis minor space
11.1.20.2	***If 'Duplex performed' is not equal to 'Venous' answer this question:***<br>Arterial patency, in rest	☐ Patent<br>☐ Stenotic<br>☐ Occluded
11.1.20.2.1	***If 'Arterial patency, in rest' is equal to 'Stenotic' answer this question:***<br>Stenosis of	☐ Subclavian artery<br>☐ Axillary artery<br>☐ Brachial artery<br>☐ Radial artery<br>☐ Ulnar artery
11.1.20.2.2	***If 'Arterial patency, in rest' is equal to 'Occluded' answer this question:***<br>Occlusion of	☐ Subclavian artery<br>☐ Axillary artery<br>☐ Brachial artery<br>☐ Radial artery<br>☐ Ulnar artery
11.1.20.3	***If 'Duplex performed' is not equal to 'Venous' answer this question:***<br>Aneurysm present	○ Yes<br>○ No
11.1.20.3.1	***If 'Aneurysm present' is equal to 'Yes' answer this question:***<br>Location aneurysm?	☐ Subclavian artery<br>☐ Axillary artery<br>☐ Brachial artery<br>☐ Radial artery<br>☐ Ulnar artery
11.1.20.3.2	***If 'Aneurysm present' is equal to 'Yes' answer this question:***<br>Thrombus present in aneurysm?	○ Yes<br>○ No<br>○ Unknown

11.1.20.3.3	***If 'Aneurysm present' is equal to 'Yes' answer this question:***<br>Maximum diameter of aneurysm	[ ] millimeter
11.4	Duplex provocation & arterial	
11.4.1	***If 'Duplex provocation & arterial' is equal to '1' answer this question:***<br>Arterial compression with provocative maneuvres	○ Conform assesment in rest<br>○ Compression, 1-50% lumen reduction<br>○ Compression, 51-99% lumen reduction<br>○ Compression, not patent<br>○ Not judgeable
11.5	calc ipsilateral compression arterial duplex	
11.5.1	***If 'calc ipsilateral compression arterial duplex' is equal to '1' answer this question:***<br>Location compression	○ Costoclavicular junction<br>○ Pectoralis minor space<br>○ Both costoclavicular junction and pectoralis minor space
11.1.20.4	***If 'Duplex performed' is not equal to 'Venous' answer this question:***<br>Distal arterial doppler signals, in rest	○ Normal (bi- or trifasic)<br>○ Abnormal<br>○ No flow<br>○ Not tested
11.4.2	***If 'Duplex provocation & arterial' is equal to '1' answer this question:***<br>Distal arterial doppler signals with provocative testing	○ Conform assesment in rest<br>○ Decreased signals<br>○ No flow

12. Presentation - Diagnostic modalities II

Number	Question	Answers
12.1	***If 'Performed diagnostic modalities' is equal to 'CT' answer this question:***<br>Date CT<br>*Warning shown if field's value is larger than NOW: 'Date cannot be in the future'*	[ ] [ ] [ ] *(dd-mm-yyyy)*
12.2	***If 'Performed diagnostic modalities' is equal to 'CT' answer this question:***<br>Was CT performed with provocative maneuvres?	○ Yes<br>○ No

12.3	***If 'Performed diagnostic modalities' is equal to 'CT' answer this question:***<br>Arterial and/or venous CT?	◯ Arterial<br>◯ Venous<br>◯ Arterial & venous
12.3.1	***If 'Arterial and/or venous CT?' is not equal to 'Arterial' answer this question:***<br>Ipsilateral vein patency with arm adducted	☐ Patent<br>☐ Stenotic<br>☐ Occluded
12.3.1.1	***If 'Ipsilateral vein patency with arm adducted' is equal to 'Stenotic' answer this question:***<br>Location venous stenosis	☐ Subclavian vein<br>☐ Axillary vein<br>☐ Brachial vein<br>☐ Cephalic vein<br>☐ Basilic vein<br>☐ Brachiocephalic vein
12.3.1.2	***If 'Ipsilateral vein patency with arm adducted' is equal to 'Occluded' answer this question:***<br>Location venous occlusion	☐ Subclavian vein<br>☐ Axillary vein<br>☐ Brachial vein<br>☐ Cephalic vein<br>☐ Basilic vein<br>☐ Brachiocephalic vein
12.4	calc ipsilateral compression CT	
12.5	Calc contralteral compression CT	
12.2.1	***If 'Was CT performed with provocative maneuvres?' is equal to 'Yes' answer this question:***<br>Ipsilateral subclavian vein compression with arm abducted	◯ Not judgeable/not scanned<br>◯ Conform assesment in rest<br>◯ Compression, 1-50% diameter reduction<br>◯ Compression, 51-99% diameter reduction<br>◯ Compression, 100% diameter reduction
12.4.1	***If 'calc ipsilateral compression CT' is equal to '1' answer this question:***<br>Location ipsilateral venous compression	◯ Costoclavicular junction<br>◯ Pectoralis minor space<br>◯ Both costoclavicular junction and pectoralis minor space
12.2.2	***If 'Was CT performed with provocative maneuvres?' is equal to 'Yes' answer this question:***<br>Contralateral subclavian vein compression with arm abducted	◯ Not judgeable/not scanned<br>◯ Conform assesment in rest<br>◯ Compression, 1-50% diameter reduction<br>◯ Compression, 51-99% diameter reduction<br>◯ Compression, 100% diameter reduction

12.5.1	***If 'Calc contralteral compression CT' is equal to '1' answer this question:*** Location contralteral venous compression	◯ Costoclavicular junction<br>◯ Pectoralis minor space<br>◯ Both costoclavicular junction and pectoralis minor space
12.3.2	***If 'Arterial and/or venous CT?' is not equal to 'Venous' answer this question:*** Which ipsilateral arteries were assesed?	☐ Subclavian artery<br>☐ Axillary artery<br>☐ Brachial artery<br>☐ Ulnar artery<br>☐ Radial artery<br>☐ Hand arch<br>☐ Digital arteries
12.3.2.1	***If 'Which ipsilateral arteries were assesed?' is equal to 'Subclavian artery' answer this question:*** ipsilateral Subclavian artery with arm adducted	☐ Normal<br>☐ Thrombus present<br>☐ Stenotic<br>☐ Occluded<br>☐ Aneurysm present
12.3.2.1.1	***If 'ipsilateral Subclavian artery with arm adducted' is equal to 'Aneurysm present' answer this question:*** Thrombus present in aneurysm	☐ Yes<br>☐ No<br>☐ Unknown
12.3.2.1.2	***If 'ipsilateral Subclavian artery with arm adducted' is equal to 'Aneurysm present' answer this question:*** Maximum diameter of aneurysm	[ ] milimeter
12.2.3	***If 'Was CT performed with provocative maneuvres?' is equal to 'Yes' answer this question:*** Ipsilateral subclavian artery compression with arm abducted	◯ Not judgeable/not scanned<br>◯ Conform assesment in rest<br>◯ Compression, 1-50% diameter reduction<br>◯ Compression, 51-99% diameter reduction<br>◯ Compression, 100% diameter reduction
12.2.4	***If 'Was CT performed with provocative maneuvres?' is equal to 'Yes' answer this question:*** Contralateral subclavian artery compression with arm abducted	◯ Not judgeable/not scanned<br>◯ Conform assesment in rest<br>◯ Compression, 1-50% diameter reduction<br>◯ Compression, 51-99% diameter reduction<br>◯ Compression, 100% diameter reduction
12.3.2.2	***If 'Which ipsilateral arteries were assesed?' is equal to 'Axillary artery' answer this question:*** Ipsilateral axillary artery	☐ Normal<br>☐ Thrombus present<br>☐ Stenotic<br>☐ Occluded

12.3.2.3	***If 'Which ipsilateral arteries were assesed?' is equal to 'Brachial artery' answer this question:***<br>Ipsilateral brachial artery	☐ Normal<br>☐ Thrombus present<br>☐ Stenotic<br>☐ Occluded
12.3.2.4	***If 'Which ipsilateral arteries were assesed?' is equal to 'Ulnar artery' answer this question:***<br>Ipsilateral ulnar artery	☐ Normal<br>☐ Thrombus present<br>☐ Stenotic<br>☐ Occluded
12.3.2.5	***If 'Which ipsilateral arteries were assesed?' is equal to 'Radial artery' answer this question:***<br>Ipsilateral radial artery	☐ Normal<br>☐ Thrombus present<br>☐ Stenotic<br>☐ Occluded
12.3.2.6	***If 'Which ipsilateral arteries were assesed?' is equal to 'Hand arch' answer this question:***<br>Ipsilateral hand arch	☐ Normal<br>☐ Thrombus present<br>☐ Stenotic<br>☐ Occluded
12.3.2.7	***If 'Which ipsilateral arteries were assesed?' is equal to 'Digital arteries' answer this question:***<br>Ipsilateral digital arteries	☐ Normal<br>☐ Thrombus present<br>☐ Stenotic<br>☐ Occluded
12.3.2.7.1	***If 'Ipsilateral digital arteries' is not equal to 'Normal' answer this question:***<br>Which digiti are affected?	☐ Dig 1<br>☐ Dig 2<br>☐ Dig 3<br>☐ Dig 4<br>☐ Dig 5
12.6	***If 'Performed diagnostic modalities' is equal to 'CT' answer this question:***<br>Other relevant CT findings?	
12.7	***If 'Performed diagnostic modalities' is equal to 'MRI' answer this question:***<br>date MRI	___ ___ ___ *(dd-mm-yyyy)*
12.8	***If 'Performed diagnostic modalities' is equal to 'MRI' answer this question:***<br>Was MRI performed with provocative testing	○ Yes<br>○ No

12.9	***If 'Performed diagnostic modalities' is equal to 'MRI' answer this question:***<br>Arterial and/or venous? Or MRI plexus?	◯ Arterial<br>◯ Venous<br>◯ Arterial & venous<br>◯ MRI plexus
12.10	MRI arterial	
12.11	MRI venous	
12.11.1	***If 'MRI venous' is equal to '1' answer this question:***<br>Ipsilateral vein patency with arm adducted	☐ Patent<br>☐ Stenotic<br>☐ Occluded
12.11.1.1	***If 'Ipsilateral vein patency with arm adducted' is equal to 'Stenotic' answer this question:***<br>Location venous stenosis	☐ Subclavian vein<br>☐ Axillary vein<br>☐ Brachial vein<br>☐ Cephalic vein<br>☐ Basilic vein<br>☐ Brachiocephalic vein
12.11.1.2	***If 'Ipsilateral vein patency with arm adducted' is equal to 'Occluded' answer this question:***<br>Location venous occlusion	☐ Subclavian vein<br>☐ Axillary vein<br>☐ Brachial vein<br>☐ Cephalic vein<br>☐ Basilic vein<br>☐ Brachiocephalic vein
12.12	calc ipsilateral compression MRI	
12.13	calc contralteral compression MRI	
12.8.1	***If 'Was MRI performed with provocative testing' is equal to 'Yes' answer this question:***<br>Ipsilateral subclavian vein compression with arm abducted	◯ Not judgeable/not scanned<br>◯ Conform assesment in rest<br>◯ Compression, 1-50% diameter reduction<br>◯ Compression, 51-99% diameter reduction<br>◯ Compression, 100% diameter reduction
12.12.1	***If 'calc ipsilateral compression MRI' is equal to '1' answer this question:***<br>Location ipsilateral venous compression	◯ Costoclavicular junction<br>◯ Pectoralis minor space<br>◯ Both costoclavicular junction and pectoralis minor space

12.8.2	***If 'Was MRI performed with provocative testing' is equal to 'Yes' answer this question:***<br>Contralateral subclavian vein compression with arm abducted	◯ Not judgeable/not scanned<br>◯ Conform assesment in rest<br>◯ Compression, 1-50% diameter reduction<br>◯ Compression, 51-99% diameter reduction<br>◯ Compression, 100% diameter reduction
12.13.1	***If 'calc contralteral compression MRI' is equal to '1' answer this question:***<br>Location contralateral venous compression	◯ Costoclavicular junction<br>◯ Pectoralis minor space<br>◯ Both costoclavicular junction and pectoralis minor space
12.10.1	***If 'MRI arterial' is equal to '1' answer this question:***<br>Which ipsilateral arteries were assesed?	☐ Subclavian artery<br>☐ Axillary artery<br>☐ Brachial artery<br>☐ Ulnar artery<br>☐ Radial artery<br>☐ Hand arch<br>☐ Digital arteries
12.10.1.1	***If 'Which ipsilateral arteries were assesed?' is equal to 'Subclavian artery' answer this question:***<br>Ipsilateral subclavian artery with arm adducted	☐ Normal<br>☐ Thrombus present<br>☐ Stenotic<br>☐ Occluded<br>☐ Aneurysm present
12.10.1.1.1	***If 'Ipsilateral subclavian artery with arm adducted' is equal to 'Aneurysm present' answer this question:***<br>Thrombus present in aneurysm?	◯ Yes<br>◯ No<br>◯ Unknown
12.10.1.1.2	***If 'Ipsilateral subclavian artery with arm adducted' is equal to 'Aneurysm present' answer this question:***<br>Maximum diameter of aneurysm	[ ] millimeter
12.8.3	***If 'Was MRI performed with provocative testing' is equal to 'Yes' answer this question:***<br>Ipsilateral subclavian artery compression with arm abducted	◯ Not judgeable/not scanned<br>◯ Conform assesment in rest<br>◯ Compression, 1-50% diameter reduction<br>◯ Compression, 51-99% diameter reduction<br>◯ Compression, 100% diameter reduction
12.8.4	***If 'Was MRI performed with provocative testing' is equal to 'Yes' answer this question:***<br>Contralateral subclavian artery compression with arm abducted	◯ Not judgeable/not scanned<br>◯ Conform assesment in rest<br>◯ Compression, 1-50% diameter reduction<br>◯ Compression, 51-99% diameter reduction<br>◯ Compression, 100% diameter reduction

12.10.1.2	***If 'Which ipsilateral arteries were assesed?' is equal to 'Axillary artery' answer this question:***<br>Ipsilateral axillary artery	☐ Normal<br>☐ Thrombus present<br>☐ Stenotic<br>☐ Occluded
12.10.1.3	***If 'Which ipsilateral arteries were assesed?' is equal to 'Brachial artery' answer this question:***<br>Ipsilateral brachial artery	☐ Normal<br>☐ Thrombus present<br>☐ Stenotic<br>☐ Occluded
12.10.1.4	***If 'Which ipsilateral arteries were assesed?' is equal to 'Ulnar artery' answer this question:***<br>Ipsilateral ulnar artery	☐ Normal<br>☐ Thrombus present<br>☐ Stenotic<br>☐ Occluded
12.10.1.5	***If 'Which ipsilateral arteries were assesed?' is equal to 'Radial artery' answer this question:***<br>Ipsilateral radial artery	☐ Normal<br>☐ Thrombus present<br>☐ Stenotic<br>☐ Occluded
12.10.1.6	***If 'Which ipsilateral arteries were assesed?' is equal to 'Hand arch' answer this question:***<br>Ipsilateral hand arch	☐ Normal<br>☐ Thrombus present<br>☐ Stenotic<br>☐ Occluded
12.10.1.7	***If 'Which ipsilateral arteries were assesed?' is equal to 'Digital arteries' answer this question:***<br>Ipsilateral digital arteries	☐ Normal<br>☐ Thrombus present<br>☐ Stenotic<br>☐ Occluded
12.10.1.7.1	***If 'Ipsilateral digital arteries' is not equal to 'Normal' answer this question:***<br>Which digiti are affected?	☐ Dig 1<br>☐ Dig 2<br>☐ Dig 3<br>☐ Dig 4<br>☐ Dig 5
12.9.1	***If 'Arterial and/or venous? Or MRI plexus?' is equal to 'MRI plexus' answer this question:***<br>Any plexus/nerve abnormalities	

12.14	***If 'Performed diagnostic modalities' is equal to 'MRI' answer this question:*** Other relevant MR findings?	

13. Presentation - Diagnostic modalities III

Number	Question	Answers
13.1	***If 'Performed diagnostic modalities' is equal to 'Venography (with or without IVUS)' answer this question:*** Date venography	(dd-mm-yyyy)
13.2	***If 'Performed diagnostic modalities' is equal to 'Venography (with or without IVUS)' answer this question:*** Was venography performed with provocative testing	Yes<br>No
13.2.1	***If 'Was venography performed with provocative testing' is equal to 'Yes' answer this question:*** Contralateral vein assessed with provocative testing?	Yes<br>No
13.3	***If 'Performed diagnostic modalities' is equal to 'Venography (with or without IVUS)' answer this question:*** Combined with intravascular ultrasound (IVUS)?	Yes<br>No
13.4	***If 'Performed diagnostic modalities' is equal to 'Venography (with or without IVUS)' answer this question:*** Vein patency, in rest	Patent<br>Stenotic<br>Occluded
13.4.1	***If 'Vein patency, in rest' is equal to 'Stenotic' answer this question:*** Location stenosis	Subclavian vein<br>Axillary vein<br>Brachial vein<br>Cephalic vein<br>Basilic vein<br>Brachiocephalic vein

13.4.2	***If 'Vein patency, in rest' is equal to 'Occluded' answer this question:***<br>Location occlusion	☐ Subclavian vein<br>☐ Axillary vein<br>☐ Brachial vein<br>☐ Cephalic vein<br>☐ Basilic vein<br>☐ Brachiocephalic vein
13.2.2	***If 'Was venography performed with provocative testing' is equal to 'Yes' answer this question:***<br>Ipsilateral subclavian vein compression with provocative testing	○ Conform assesment in rest<br>○ Compression, 1-50% lumen reduction<br>○ Compression, 51-99% lumen reduction<br>○ Compression, not patent<br>○ Not judgeable
13.2.2.1	***If 'Ipsilateral subclavian vein compression with provocative testing' is not equal to 'Conform assesment in rest' answer this question:***<br>Location ipsilateral compression	○ Costoclavicular junction<br>○ Pectoralis minor space<br>○ Both costoclavicular junction and pectoralis minor space
13.2.1.1	***If 'Contralateral vein assessed with provocative testing?' is equal to 'Yes' answer this question:***<br>Contralateral subclavian vein compression with provocative testing	○ Conform assesment in rest<br>○ Compression, 1-50% lumen reduction<br>○ Compression, 51-99% lumen reduction<br>○ Compression, not patent<br>○ Not judgeable
13.2.1.1.1	***If 'Contralateral subclavian vein compression with provocative testing' is not equal to 'Conform assesment in rest' answer this question:***<br>Location contralateral compression	○ Costoclavicular junction<br>○ Pectoralis minor space<br>○ Both costoclavicular junction and pectoralis minor space
13.5	***If 'Performed diagnostic modalities' is equal to 'Venography (with or without IVUS)' answer this question:***<br>Other relevant venography findings?	
13.3.1	***If 'Combined with intravascular ultrasound (IVUS)?' is equal to 'Yes' answer this question:***<br>Any relevant IVUS findings?	
13.6	***If 'Performed diagnostic modalities' is equal to 'Arteriography (with or without IVUS)' answer this question:***<br>Date arteriography	___ ___ ___ *(dd-mm-yyyy)*

13.7	***If 'Performed diagnostic modalities' is equal to 'Arteriography (with or without IVUS)' answer this question:*** Which arteries were assessed?	☐ Subclavian artery<br>☐ Axillary artery<br>☐ Brachial artery<br>☐ Ulnar artery<br>☐ Radial artery<br>☐ Hand arch<br>☐ Digital arteries
13.8	***If 'Performed diagnostic modalities' is equal to 'Arteriography (with or without IVUS)' answer this question:*** Was arteriography performed with provocative maneuvres?	○ Yes<br>○ No
13.8.1	***If 'Was arteriography performed with provocative maneuvres?' is equal to 'Yes' answer this question:*** Was contralateral subclavian artery assessed with provocative testing?	○ Yes<br>○ No
13.9	***If 'Performed diagnostic modalities' is equal to 'Arteriography (with or without IVUS)' answer this question:*** Combined with intravascular ultrasound (IVUS)?	○ Yes<br>○ No
13.7.1	***If 'Which arteries were assessed?' is equal to 'Subclavian artery' answer this question:*** Subclavian artery	☐ Not judgeable/not imaged<br>☐ Normal<br>☐ Thrombus present<br>☐ Stenotic<br>☐ Occluded<br>☐ Aneurysm present
13.7.1.1	***If 'Subclavian artery' is equal to 'Aneurysm present' answer this question:*** Thrombus present in aneurysm?	○ Yes<br>○ No<br>○ Unknown
13.7.1.2	***If 'Subclavian artery' is equal to 'Aneurysm present' answer this question:*** Maximum diamter of aneurysm (if not measured, choose 0)	____ Milimeter
13.8.2	***If 'Was arteriography performed with provocative maneuvres?' is equal to 'Yes' answer this question:*** Ipsilateral subclavian artery with provocative maneuvres	○ Conform assesment in rest<br>○ Compression, 1-50% lumen reduction<br>○ Compression, 51-99% lumen reduction<br>○ Compression, not patent<br>○ Not judgeable
13.8.1.1	***If 'Was contralateral subclavian artery assessed with provocative testing?' is equal to 'Yes' answer this question:*** Contralateral subclavian artery with provocative maneuvres	○ Conform assesment in rest<br>○ Compression, 1-50% lumen reduction<br>○ Compression, 51-99% lumen reduction<br>○ Compression, not patent<br>○ Not judgeable

13.7.2	***If 'Which arteries were assessed?' is equal to 'Axillary artery' answer this question:***<br>Axillary artery	☐ Not judgeable/not imaged<br>☐ Normal<br>☐ Thrombus present<br>☐ Stenotic<br>☐ Occluded
13.7.3	***If 'Which arteries were assessed?' is equal to 'Brachial artery' answer this question:***<br>Brachial artery	☐ Not judgeable/not imaged<br>☐ Normal<br>☐ Thrombus present<br>☐ Stenotic<br>☐ Occluded
13.7.4	***If 'Which arteries were assessed?' is equal to 'Ulnar artery' answer this question:***<br>Ulnar artery	☐ Not judgeable/not imaged<br>☐ Normal<br>☐ Thrombus present<br>☐ Stenotic<br>☐ Occluded
13.7.5	***If 'Which arteries were assessed?' is equal to 'Radial artery' answer this question:***<br>Radial artery	☐ Not judgeable/not imaged<br>☐ Normal<br>☐ Thrombus present<br>☐ Stenotic<br>☐ Occluded
13.7.6	***If 'Which arteries were assessed?' is equal to 'Hand arch' answer this question:***<br>Hand arch	☐ Not judgeable/not imaged<br>☐ Normal<br>☐ Thrombus present<br>☐ Stenotic<br>☐ Occluded
13.7.7	***If 'Which arteries were assessed?' is equal to 'Digital arteries' answer this question:***<br>Digital arteries	☐ Not judgeable/not imaged<br>☐ Normal<br>☐ Thrombus present<br>☐ Stenotic<br>☐ Occluded
13.10	calc digiti affected	
13.10.1	***If 'calc digiti affected' is not equal to '1' answer this question:***<br>Which dig(iti) is/are affected?	☐ Dig 1<br>☐ Dig 2<br>☐ Dig 3<br>☐ Dig 4<br>☐ Dig 5

13.11	***If 'Performed diagnostic modalities' is equal to 'Arteriography (with or without IVUS)' answer this question:***<br>Other relevant arteriography findings?	
13.9.1	***If 'Combined with intravascular ultrasound (IVUS)?' is equal to 'Yes' answer this question:***<br>Any relevant IVUS findings?	
13.12	***If 'Performed diagnostic modalities' is equal to 'Other' answer this question:***<br>Please describe other diagnostic modality and findings	
13.13	Anatomical abnormality present, left side	☐ No<br>☐ Cervical rib<br>☐ Prominent transverse process C7<br>☐ Abnormal clavicle<br>☐ Abnormal first rib<br>☐ Other<br>☐ Unknown
13.13.1	***If 'Anatomical abnormality present, left side' is equal to 'Other' answer this question:***<br>Please describe other anatomical abnormality, left side	
13.14	Anatomical abnormality present, right side	☐ No<br>☐ Cervical rib<br>☐ Prominent transverse process C7<br>☐ Abnormal clavicle<br>☐ Abnormal first rib<br>☐ Other<br>☐ Unknown
13.14.1	***If 'Anatomical abnormality present, right side' is equal to 'Other' answer this question:***<br>Please describe other anatomical abnormality, right side	
13.15	***If 'calculation, vTOS+pUEDVT' is equal to '1' answer this question:***<br>Pulmonary embolus present?	○ Yes<br>○ No<br>○ Unknown

14. Treatment - Treatment

Number	Question	Answers
14.1	Primary treatment plan<br>Examples:<br><br>Treatment for **nTOS** with physical therapy and local injection, no surgery planned: the eCRF can be completed directly after the injection and the start of physical therapy.<br><br>Treatment of **idiopatic upper extremity deep venous thrombosis** with compression stockings and oral anticoagulation (no surgery or endovascular interventions planned): the eCRF can be completed directly after the start of both therapies.<br><br>Treatment of nTOS with decompression surgery: this eCRF can be completed atleast 30 days after surgery.<br><br>Treatment of **a/vTOS** with thrombolysis and decompression surgery followed by stent placement: this eCRF can be completed atleast 30 days after surgery and after stent placement.	
14.2	Is the primary treatment plan completely deployed?<br>*Warning shown if field's value is equal to No: 'Please don't continue this eCRF until the primary treatment plan is finished (see description above). Please provide the date you expect the primary treatment plan to be finished in the next question.'*	◯ Yes<br>◯ No
14.2.1	***If 'Is the primary treatment plan completely deployed?' is equal to 'No' answer this question:***<br>Date you expect the primary treatment plan to be completely deployed, you will receive a reminder via email to complete the eCRF.	☐ ☐ ☐ *(dd-mm-yyyy)*
14.2.2	***If 'Is the primary treatment plan completely deployed?' is equal to 'No' answer this question:***<br>Please leave this text box open	☐
	**The next questions includes treatment started or performed in the referring hospital. For example thrombolysis in referring hospital followed by surgery in your hospital**	
14.3	***If 'Pulmonary embolus present?' is equal to 'Yes' answer this question:***<br>Any treatment for pulmonary embolus?<br>Please describe as detailed as possible any additional treatment for pulmonary embolus, including hospital/IC admission, thrombolysis etc.	☐

14.2.3	***If 'Is the primary treatment plan completely deployed?' is equal to 'Yes' answer this question:***<br>What treatment(s) was/were started or performed?	☐ Physical therapy<br>☐ Compression stockings<br>☐ Mensendieck therapy<br>☐ Oral antithrombotic therapy<br>☐ Other medication besides antithrombotics<br>☐ Local injection with botox/anesthetic<br>☐ Endovascular intervention(s)<br>☐ Surgical intervention(s)<br>☐ Other<br>☐ No treatment
	**Medication started**	
14.2.3.2	***If 'What treatment(s) was/were started or performed?' is equal to 'Oral antithrombotic therapy' answer this question:***<br>Which antithrombotic(s)? See 'i' for additional information. Please provide all different types of antithrombotics described, even if an antithrombotic is already stopped. You are able to provide the stop dates if applicable.<br><br>Examples: Heparin and Vitamin K antagonist described, heparin stopped when INR is adequate. Please choose heparin AND Vitamin K antagonist and provide the stop date of heparin. Heparin after thrombolysis until surgery, after surgery DOAC. Please choose Heparin AND DOAC and provide the stop date of heparin.	☐ Acetylsalicylic acid<br>☐ ADP inhibitor (e.g. clopidogrel)<br>☐ Vitamin K antagonist (e.g. acenocoumarol)<br>☐ Directly acting oral anticoagulants (DOACs)<br>☐ Low molecular weight heparin<br>☐ Other
14.2.3.2.1	***If 'Which antithrombotic(s)? See 'i' for additional information.' is equal to 'Acetylsalicylic acid' answer this question:***<br>Which acetylsalicylic acid, including dosage	○ Acetylsalicylic acid 30mg (neuro)<br>○ Acetylsalicylic acid 80mg (cardio)<br>○ Acetylsalicylic acid 100mg (aspirin protect)<br>○ Carbasalate calcium 38mg (ascal 38)<br>○ Carbasalate calcium 100mg (ascal cardio)<br>○ Other
14.2.3.2.1.1	***If 'Which acetylsalicylic acid, including dosage' is equal to 'Other' answer this question:***<br>Which other acetylsalicyl acid, including dosage	
14.2.3.2.2	***If 'Which antithrombotic(s)? See 'i' for additional information.' is equal to 'Acetylsalicylic acid' answer this question:***<br>Date acetylsalicylic acid started<br>If precise date is unknown:<br><br>5-2020 -> 01-05-2020<br><br>2020 -> 01-01-2020	___ ___ ___ *(dd-mm-yyyy)*

14.2.3.2.3	***If 'Which antithrombotic(s)? See 'i' for additional information.' is equal to 'Acetylsalicylic acid' answer this question:***<br>Is acetylsalicylic acid already stopped?	◯ Yes<br>◯ No
14.2.3.2.3.1	***If 'Is acetylsalicylic acid already stopped?' is equal to 'Yes' answer this question:***<br>Date acetylsalicylic acid stopped	*(dd-mm-yyyy)*
14.2.3.2.4	***If 'Which antithrombotic(s)? See 'i' for additional information.' is equal to 'ADP inhibitor (e.g. clopidogrel)' answer this question:***<br>Which ADP inhibitor, including dosage	◯ Clopidogrel 75mg<br>◯ Ticagrelor 60mg<br>◯ Ticagrelor 90mg<br>◯ Prasugrel 5mg<br>◯ Prasugrel 10mg<br>◯ Other
14.2.3.2.4.1	***If 'Which ADP inhibitor, including dosage' is equal to 'Other' answer this question:***<br>Which other ADP inhibitor, including dosage	
14.2.3.2.5	***If 'Which antithrombotic(s)? See 'i' for additional information.' is equal to 'ADP inhibitor (e.g. clopidogrel)' answer this question:***<br>Date ADP inhibitor started<br>If precise date is unknown:<br>5-2020 -> 01-05-2020<br>2020 -> 01-01-2020	*(dd-mm-yyyy)*
14.2.3.2.6	***If 'Which antithrombotic(s)? See 'i' for additional information.' is equal to 'ADP inhibitor (e.g. clopidogrel)' answer this question:***<br>Is ADP inhibitor already stopped?	◯ Yes<br>◯ No
14.2.3.2.6.1	***If 'Is ADP inhibitor already stopped?' is equal to 'Yes' answer this question:***<br>Date ADP inhibitor stopped<br>If precise date is unknown:<br>5-2020 -> 01-05-2020<br>2020 -> 01-01-2020	*(dd-mm-yyyy)*
14.2.3.2.7	***If 'Which antithrombotic(s)? See 'i' for additional information.' is equal to 'Vitamin K antagonist (e.g. acenocoumarol)' answer this question:***<br>Which vitamin K antagonist	◯ Acenocoumarol<br>◯ Phenprocoumon<br>◯ Warfarin<br>◯ Other

14.2.3.2.7.1	***If 'Which vitamin K antagonist' is equal to 'Other' answer this question:***<br>Which other vitamin K antagonsit, including dosage	
14.2.3.2.8	***If 'Which antithrombotic(s)? See 'i' for additional information.' is equal to 'Vitamin K antagonist (e.g. acenocoumarol)' answer this question:***<br>Date vitamin K antagonist started<br>If precise date is unknown:<br>5-2020 -> 01-05-2020<br>2020 -> 01-01-2020	*(dd-mm-yyyy)*
14.2.3.2.9	***If 'Which antithrombotic(s)? See 'i' for additional information.' is equal to 'Vitamin K antagonist (e.g. acenocoumarol)' answer this question:***<br>Is vitamin K antagonist already stopped?	◯ Yes<br>◯ No
14.2.3.2.9.1	***If 'Is vitamin K antagonist already stopped?' is equal to 'Yes' answer this question:***<br>Date vitamin K antagonist stopped	*(dd-mm-yyyy)*
14.2.3.2.10	***If 'Which antithrombotic(s)? See 'i' for additional information.' is equal to 'Directly acting oral anticoagulants (DOACs)' answer this question:***<br>Which DOAC, including dosage	◯ Rivaroxaban, 15mg twice daily for 21 days followed by 20mg daily<br>◯ Rivaroxaban 20mg daily<br>◯ Rivaroxaban 10mg daily<br>◯ Rivaroxaban 2.5mg twice daily<br>◯ Apixaban 10mg twice daily for 7 days followed by 5mg twice daily<br>◯ Apixaban 5mg twice daily<br>◯ Apixaban 2.5mg twice daily<br>◯ Edoxaban 60mg daily<br>◯ Dabigatran 150mg twice daily<br>◯ Other
14.2.3.2.10.1	***If 'Which DOAC, including dosage' is equal to 'Other' answer this question:***<br>Which other DOAC, including dosage	
14.2.3.2.11	***If 'Which antithrombotic(s)? See 'i' for additional information.' is equal to 'Directly acting oral anticoagulants (DOACs)' answer this question:***<br>Date directly acting oral anticoagulant (DOAC) started<br>If precise date is unknown:<br>5-2020 -> 01-05-2020<br>2020 -> 01-01-2020	*(dd-mm-yyyy)*

14.2.3.2.12	***If 'Which antithrombotic(s)? See 'i' for additional information.' is equal to 'Directly acting oral anticoagulants (DOACs)' answer this question:***<br>Is DOAC already stopped?	○ Yes<br>○ No
14.2.3.2.12.1	***If 'Is DOAC already stopped?' is equal to 'Yes' answer this question:***<br>Date DOAC stopped<br>If precise date is unknown:<br>5-2020 -> 01-05-2020<br>2020 -> 01-01-2020	*(dd-mm-yyyy)*
14.2.3.2.13	***If 'Which antithrombotic(s)? See 'i' for additional information.' is equal to 'Low molecular weight heparin' answer this question:***<br>Which low molecular weight heparin, including dosage	○ Dalteparin (Fragmin) 200 IU/kg once daily<br>○ Nadroparin (Fraxiparine) 86 UI/kg twice daily<br>○ Enoxaparin (Clexane) 150UI/kg once daily<br>○ Tinzaparin (Innohep) 175 UI/kg once daily<br>○ Other
14.2.3.2.13.1	***If 'Which low molecular weight heparin, including dosage' is equal to 'Other' answer this question:***<br>Which other low molecular weight heparin, including dosage	
14.2.3.2.14	***If 'Which antithrombotic(s)? See 'i' for additional information.' is equal to 'Low molecular weight heparin' answer this question:***<br>Date low molecular weight heparin started<br>If precise date is unknown:<br>5-2020 -> 01-05-2020<br>2020 -> 01-01-2020	*(dd-mm-yyyy)*
14.2.3.2.15	***If 'Which antithrombotic(s)? See 'i' for additional information.' is equal to 'Low molecular weight heparin' answer this question:***<br>Is low molecular weigth heparin already stopped?	○ Yes<br>○ No
14.2.3.2.15.1	***If 'Is low molecular weigth heparin already stopped?' is equal to 'Yes' answer this question:***<br>Date low molecular weight heparin stopped<br>If precise date is unknown:<br>5-2020 -> 01-05-2020<br>2020 -> 01-01-2020	*(dd-mm-yyyy)*
14.2.3.2.16	***If 'Which antithrombotic(s)? See 'i' for additional information.' is equal to 'Other' answer this question:***<br>Which other antithrombotic, including dosage	

14.2.3.2.17	***If 'Which antithrombotic(s)? See 'i' for additional information.' is equal to 'Other' answer this question:***<br>Date 'other antithrombotic' started<br>If precise date is unknown:<br>5-2020 -> 01-05-2020<br>2020 -> 01-01-2020	[ ] [ ] [ ] *(dd-mm-yyyy)*
14.2.3.2.18	***If 'Which antithrombotic(s)? See 'i' for additional information.' is equal to 'Other' answer this question:***<br>Is 'other antithrombotic' already stopped?	○ Yes<br>○ No
14.2.3.2.18.1	***If 'Is 'other antithrombotic' already stopped?' is equal to 'Yes' answer this question:***<br>Date 'other antithrombotic' stopped<br>If precise date is unknown:<br>5-2020 -> 01-05-2020<br>2020 -> 01-01-2020	[ ] [ ] [ ] *(dd-mm-yyyy)*
14.2.3.3	***If 'What treatment(s) was/were started or performed?' is equal to 'Oral antithrombotic therapy' answer this question:***<br>Estimated (or planned) duration of antithrombotic therapy	○ Three months<br>○ Six months<br>○ One year<br>○ Lifetime<br>○ Other
14.2.3.3.1	***If 'Estimated (or planned) duration of antithrombotic therapy' is equal to 'Other' answer this question:***<br>Duration, other	[ ]
14.2.3.4	***If 'What treatment(s) was/were started or performed?' is equal to 'Other medication besides antithrombotics' answer this question:***<br>Medication started (besides antithrombotics)	[ ]
	**Local injection**	
14.2.3.6	***If 'What treatment(s) was/were started or performed?' is equal to 'Local injection with botox/anesthetic' answer this question:***<br>Date local injection	[ ] [ ] [ ] *(dd-mm-yyyy)*
14.2.3.7	***If 'What treatment(s) was/were started or performed?' is equal to 'Local injection with botox/anesthetic' answer this question:***<br>Which agent was injected	☐ Botulinium toxin<br>☐ Steroids<br>☐ Local anestheticum<br>☐ Other<br>☐ Unknown

14.2.3.7.1	***If 'Which agent was injected' is equal to 'Other' answer this question:*** Which other agent	
14.2.3.8	***If 'What treatment(s) was/were started or performed?' is equal to 'Local injection with botox/anesthetic' answer this question:*** Dosage of agent	
14.2.3.9	***If 'What treatment(s) was/were started or performed?' is equal to 'Local injection with botox/anesthetic' answer this question:*** Which structure was injected?	☐ Anterior scalene muscle<br>☐ Middle scalene muscle<br>☐ Subclavius muscle<br>☐ Pectoralis minor<br>☐ Other<br>☐ Unknown
14.2.3.9.1	***If 'Which structure was injected?' is equal to 'Other' answer this question:*** Which other structure	
14.2.3.10	***If 'What treatment(s) was/were started or performed?' is equal to 'Local injection with botox/anesthetic' answer this question:*** Which technique was used?	☐ Landmarks<br>☐ EMG guidance<br>☐ Fluoroscopic guidande<br>☐ Ultrasound<br>☐ CT<br>☐ MRI<br>☐ Other<br>☐ Unknown
14.2.3.10.1	***If 'Which technique was used?' is equal to 'Other' answer this question:*** Which other technique	
	**Endovascular intervention(s)**	
	**If endovascular procedure was performed simultaneously with surgery, this can be describe in the endovascular and surgical intervention form.**	
14.2.3.13	***If 'What treatment(s) was/were started or performed?' is equal to 'Endovascular intervention(s)' answer this question:*** Venous or arterial endovascular intervention?	☐ Venous<br>☐ Arterial

14.2.3.14	***If 'What treatment(s) was/were started or performed?' is equal to 'Endovascular intervention(s)' answer this question:*** Explanation	
14.2.3.13.1	***If 'Venous or arterial endovascular intervention?' is equal to 'Venous' answer this question:*** Venous endovascular intervention form	
14.2.3.13.2	***If 'Venous or arterial endovascular intervention?' is equal to 'Arterial' answer this question:*** Arterial endovascular intervention form	
	**Surgical intervention(s)**	
14.2.3.16	***If 'What treatment(s) was/were started or performed?' is equal to 'Surgical intervention(s)' answer this question:*** Explanation	
14.2.3.17	***If 'What treatment(s) was/were started or performed?' is equal to 'Surgical intervention(s)' answer this question:*** Surgical intervention form	
	**Other therapy or therapies**	
14.2.3.19	***If 'What treatment(s) was/were started or performed?' is equal to 'Other' answer this question:*** Please describe 'other' therapies in detail	

15. Follow-up contact or event - Follow-up contact or event

Number	Question	Answers
	**Please use the 'follow-up contact/event form' to register any events in the follow-up period of this patient. For each event or hospital visit a new form can be openend.**	
15.1	Follow-up contact/event form	

Reports of Thoracic Outlet Syndrome Registry - version 244.61

Printed on 07-10-2022 14:52:39 by Ludo Schropp

Repeating Data 'Venous endovascular intervention'

Form Venous endovascular intervention

Number	Question	Answers
1	Date of endovascular intervention(s)	☐ ☐ ☐ *(dd-mm-yyyy)*
2	Was venous endovascular intervention performed simultaneously with surgical intervention?	◯ Yes<br>◯ No
3	Performed endovascular procedure(s)	☐ Local catheter guided pharmacological thrombolysis<br>☐ Pharmacomechanical thrombolysis<br>☐ Mechanical/aspiration thrombectomy<br>☐ Percutaneus transluminal venoplasty<br>☐ Stent placement<br>☐ Superior vena cava filter<br>☐ Other
4	Access site(s) during procedure	☐ Femoral vein<br>☐ Brachial vein, ipsilateral<br>☐ Cephalic vein, ipsilateral<br>☐ Basilic vein, ipsilateral<br>☐ Other
4.1	***If 'Access site(s) during procedure' is equal to 'Other' answer this question:***<br>Which other access site(s)?	☐
5	Was endovascular intervention performed intravascular ultrasound (IVUS) assisted?	◯ Yes<br>◯ No
	__Local catheter guided pharmacological thrombolysis__	

3.2	***If 'Performed endovascular procedure(s)' is equal to 'Local catheter guided pharmacological thrombolysis' answer this question:***<br>Vessel(s) treated	☐ Subclavian vein<br>☐ Axillary vein<br>☐ Brachial vein<br>☐ Cephalic vein<br>☐ Basilic vein<br>☐ Brachiocephalic vein
3.3	***If 'Performed endovascular procedure(s)' is equal to 'Local catheter guided pharmacological thrombolysis' answer this question:***<br>Technique used	○ EKOS<br>○ Pulse spray catheter<br>○ Other
3.3.1	***If 'Technique used' is equal to 'Other' answer this question:***<br>Technique, other	
3.4	***If 'Performed endovascular procedure(s)' is equal to 'Local catheter guided pharmacological thrombolysis' answer this question:***<br>Agent used	○ Alteplase<br>○ Reteplase<br>○ Urokinase<br>○ Streptokinase<br>○ Other
3.4.1	***If 'Agent used' is equal to 'Other' answer this question:***<br>Agent, other	
3.5	***If 'Performed endovascular procedure(s)' is equal to 'Local catheter guided pharmacological thrombolysis' answer this question:***<br>Dosage per hour	
3.6	***If 'Performed endovascular procedure(s)' is equal to 'Local catheter guided pharmacological thrombolysis' answer this question:***<br>Treatment duration in hours	______ Hours
	__Pharmacomechanical thrombolysis__	
3.8	***If 'Performed endovascular procedure(s)' is equal to 'Pharmacomechanical thrombolysis' answer this question:***<br>Vessel(s) treated	☐ Subclavian vein<br>☐ Axillary vein<br>☐ Brachial vein<br>☐ Cephalic vein<br>☐ Basilic vein<br>☐ Brachiocephalic vein

3.9	***If 'Performed endovascular procedure(s)' is equal to 'Pharmacomechanical thrombolysis' answer this question:***<br>Technique used	☐ Trellis<br>☐ Angiojet<br>☐ Powerpulse<br>☐ Other
3.9.1	***If 'Technique used' is equal to 'Other' answer this question:***<br>Technique, other	
3.10	***If 'Performed endovascular procedure(s)' is equal to 'Pharmacomechanical thrombolysis' answer this question:***<br>Agent used	☐ Alteplase<br>☐ Reteplase<br>☐ Urokinase<br>☐ Streptokinase<br>☐ Other
3.10.1	***If 'Agent used' is equal to 'Other' answer this question:***<br>Agent, other	
3.11	***If 'Performed endovascular procedure(s)' is equal to 'Pharmacomechanical thrombolysis' answer this question:***<br>Dosage used	
	__Mechanical/aspiration thrombectomy__	
3.13	***If 'Performed endovascular procedure(s)' is equal to 'Mechanical/aspiration thrombectomy' answer this question:***<br>Vessel(s) treated	☐ Subclavian vein<br>☐ Axillary vein<br>☐ Brachial vein<br>☐ Cephalic vein<br>☐ Basilic vein<br>☐ Brachiocephalic vein
3.14	***If 'Performed endovascular procedure(s)' is equal to 'Mechanical/aspiration thrombectomy' answer this question:***<br>Technique used	☐ Rotarex<br>☐ Thrombex PMT<br>☐ Cleaner<br>☐ Angiojet<br>☐ Manual suction<br>☐ Other

3.14.1	***If 'Technique used' is equal to 'Other' answer this question:***<br>Technique, other	
	__Percutaneous transluminal venoplasty__	
3.16	***If 'Performed endovascular procedure(s)' is equal to 'Percutaneus transluminal venoplasty' answer this question:***<br>Vessel(s) treated	☐ Subclavian vein<br>☐ Axillary vein<br>☐ Brachial vein<br>☐ Cephalic vein<br>☐ Basilic vein<br>☐ Brachiocephalic vein
3.17	***If 'Performed endovascular procedure(s)' is equal to 'Percutaneus transluminal venoplasty' answer this question:***<br>Type of balloon(s) used	☐ Noncompliant (high-pressure) balloon<br>☐ Semicompliant (midpressure) balloon<br>☐ Compliant (elastomeric) balloon<br>☐ Drug eluting balloon<br>☐ Cutting balloon<br>☐ Other<br>☐ Unknown
3.17.1	***If 'Type of balloon(s) used' is equal to 'Other' answer this question:***<br>Which other balloon type	
3.18	***If 'Performed endovascular procedure(s)' is equal to 'Percutaneus transluminal venoplasty' answer this question:***<br>Maximum diameter balloon used<br>If unknown, choose '0'	mm
	__Stent placement__	
3.20	***If 'Performed endovascular procedure(s)' is equal to 'Stent placement' answer this question:***<br>Vessel(s) treated	☐ Subclavian vein<br>☐ Axillary vein<br>☐ Brachial vein<br>☐ Cephalic vein<br>☐ Basilic vein<br>☐ Brachiocephalic vein
3.21	***If 'Performed endovascular procedure(s)' is equal to 'Stent placement' answer this question:***<br>Number of stents inserted<br>If unknown, choose '0'	Stents

3.22	***If 'Performed endovascular procedure(s)' is equal to 'Stent placement' answer this question:***<br>Maximum diameter stent used	[ ] mm
3.23	***If 'Performed endovascular procedure(s)' is equal to 'Stent placement' answer this question:***<br>Approximately total length of stented vein<br>If unknown, choose '0'	[ ] mm
3.24	***If 'Performed endovascular procedure(s)' is equal to 'Stent placement' answer this question:***<br>Type of stent(s) used	☐ Self expandable bare metal<br>☐ Balloon expandable bare metal<br>☐ Self expandable covered stent<br>☐ Balloon expandable covered stent<br>☐ Drug eluting<br>☐ Biodegradable<br>☐ Other<br>☐ Unknown
3.24.1	***If 'Type of stent(s) used' is equal to 'Other' answer this question:***<br>Which other type of stent	[ ]
3.25	***If 'Performed endovascular procedure(s)' is equal to 'Stent placement' answer this question:***<br>Please describe every imlanted stent in detal, including brand, type, material, diameter, length, and location (vessel)<br>Example:<br>Boston Scientific Venous Wallstent, self-expandable bare metal egiloy, 10mm diameter 68mm length, axillary vein.<br>BD Covera, self-expandable nitinol ePTFE covered, 8mm diameter, 40mm length, subclavian vein.	[ ]
	__'Other' endovascular procedure__	
3.27	***If 'Performed endovascular procedure(s)' is equal to 'Other' answer this question:***<br>Please describe other endovascular procedure in detail	[ ]
	__Intravascular ultrasound__	
5.2	***If 'Was endovascular intervention performed intravascular ultrasound (IVUS) assisted?' is equal to 'Yes' answer this question:***<br>Any relevant IVUS findings?	[ ]
	__Result after endovascular intervention__	

6	Were provocative maneuvers performed?	◯ Yes<br>◯ No
7	Result after endovascular intervention	◯ Fully successful with normal residual vein<br>◯ Successfull (all thrombus gone) with residual intrinsic abnormality<br>◯ Partial successful, some flow reestablished, still thrombus/stenosis present<br>◯ Partially successful, but vein remains occluded<br>◯ No improvement/unsuccessful<br>◯ Worsened
7.1	***If 'Result after endovascular intervention' is equal to 'Successfull (all thrombus gone) with residual intrinsic abnormality' answer this question:***<br>Severity of intrinsic abnormality	◯ Mild<br>◯ Moderate<br>◯ Severe
7.2	***If 'Result after endovascular intervention' is equal to 'Worsened' answer this question:***<br>Please elaborate why situation worsened	
6.1	***If 'Were provocative maneuvers performed?' is equal to 'Yes' answer this question:***<br>Compression of subclavian vein with provocative maneuvres?	◯ Yes<br>◯ No
6.1.1	***If 'Compression of subclavian vein with provocative maneuvres?' is equal to 'Yes' answer this question:***<br>Compression, abduction	◯ Compression, 1-50% lumen reduction<br>◯ Compression, 51-99% lumen reduction<br>◯ 100% lumen reduction with provocative maneuvres
8	Any other relevant remarks regarding intervention?	
	__ATTENTION: please don't press 'Close report', press 'next' instead to go to the next page and complete the venous endovascular intervention form before returning to the eCRF__	

Form Complications

Number	Question	Answers

1	Did a complication occur?	◯ Yes<br>◯ No
1.1	***If 'Did a complication occur?' is equal to 'Yes' answer this question:***<br>Type of complication(s)	☐ Bleeding complication, thrombolysis related<br>☐ Bleeding complication, not related to thrombolysis<br>☐ Periprocedural complications<br>☐ Cardiovascular complication<br>☐ Contrast related nephropathy<br>☐ Other
1.1.1	***If 'Type of complication(s)' is equal to 'Bleeding complication, thrombolysis related' answer this question:***<br>Bleeding complication, related to thrombolysis	☐ Mucocutaneous<br>☐ Gastrointestinal<br>☐ Genitourinary<br>☐ Broncho-pulmonary<br>☐ Musculo-skeletal and soft tissue<br>☐ Body cavity (pleural, peritoneal, etc)<br>☐ Central nervous system<br>☐ Invasive site bleeding (including access site)<br>☐ Other
1.1.1.1	***If 'Bleeding complication, related to thrombolysis' is equal to 'Other' answer this question:***<br>What other thrombolysis related bleeding complication?	
1.1.2	***If 'Type of complication(s)' is equal to 'Bleeding complication, thrombolysis related' answer this question:***<br>Please describe thrombolysis related bleeding complication in detail	
1.1.3	***If 'Type of complication(s)' is equal to 'Bleeding complication, thrombolysis related' answer this question:***<br>Consequence of thrombolysis related bleeding complication	☐ No consequence<br>☐ Moderate hemodynamic instability<br>☐ Severe hemodynamic instability<br>☐ End organ faillure<br>☐ Fatal bleeding, patient deceased<br>☐ Other
1.1.3.1	***If 'Consequence of thrombolysis related bleeding complication' is equal to 'Other' answer this question:***<br>Please describe other consequence in detail	

1.1.4	***If 'Type of complication(s)' is equal to 'Bleeding complication, thrombolysis related' answer this question:***<br>Treatment of thrombolysis related bleeding complication	☐ No treatment<br>☐ Red blood cell transfusion (over any routine transfusion needs)<br>☐ Intensive care unit admission<br>☐ Other
1.1.4.1	***If 'Treatment of thrombolysis related bleeding complication' is equal to 'Intensive care unit admission' answer this question:***<br>Number of days admitted to the intensive care unit	Day(s)
1.1.4.2	***If 'Treatment of thrombolysis related bleeding complication' is equal to 'Other' answer this question:***<br>Please describe other treatment(s) in detail	
1.1.5	***If 'Type of complication(s)' is equal to 'Bleeding complication, not related to thrombolysis' answer this question:***<br>Please describe non-thrombolysis related bleeding complication in detail	
1.1.6	***If 'Type of complication(s)' is equal to 'Bleeding complication, not related to thrombolysis' answer this question:***<br>Consequence of non-thrombolysis related bleeding complication	☐ No consequence<br>☐ Moderate hemodynamic instability<br>☐ Severe hemodynamic instability<br>☐ End organ faillure<br>☐ Fatal bleeding, patient deceased<br>☐ Other
1.1.6.1	***If 'Consequence of non-thrombolysis related bleeding complication' is equal to 'Other' answer this question:***<br>Please describe other consequence in detail	
1.1.7	***If 'Type of complication(s)' is equal to 'Bleeding complication, not related to thrombolysis' answer this question:***<br>Treatment of non-thrombolysis related bleeding complication	☐ No treatment<br>☐ Red blood cell transfusion (over any routine transfusion needs)<br>☐ Intensive care unit admission<br>☐ Other
1.1.7.1	***If 'Treatment of non-thrombolysis related bleeding complication' is equal to 'Intensive care unit admission' answer this question:***<br>Number of days admitted to the intensive care unit	Day(s)

1.1.7.2	***If 'Treatment of non-thrombolysis related bleeding complication' is equal to 'Other' answer this question:*** Please describe other treatment(s) in detail	
1.1.8	***If 'Type of complication(s)' is equal to 'Periprocedural complications' answer this question:*** Please describe periprocedural complication in detail, this includes etiology, consequences and treatment (including ICU admission)	
1.1.9	***If 'Type of complication(s)' is equal to 'Cardiovascular complication' answer this question:*** Please describe cardiovascular complication in detail, this includes etiology, consequences and treatment (including ICU admission)	
1.1.10	***If 'Type of complication(s)' is equal to 'Contrast related nephropathy' answer this question:*** Please describe contrast related nephropathy in detail, including treatment, dialysis and ICU admission if applicable	
1.1.11	***If 'Type of complication(s)' is equal to 'Other' answer this question:*** Please describe 'other' complication(s) in detail, including etiology, consequences and treatment (including ICU admission)	
	__When finished with the 'venous endovascular form', please click 'close report' to return to the eCRF. This form will remain visible and accessible in the left side of the screen.__	

Repeating Data 'Arterial endovascular intervention'

Form Arterial endovascular intervention(s)

Number	Question	Answers
1	Date of intervention	(dd-mm-yyyy)
2	Was arterial endovascular procedure performed simultaneously with surgical intervention?	○ Yes<br>○ No

3	Performed endovascular procedure(s)	☐ Local catheter guided pharmacological thrombolysis<br>☐ Pharmacomechanical thrombolysis<br>☐ Mechanical/aspiration thrombectomy<br>☐ Percutaneus transluminal angioplasty<br>☐ Stent placement<br>☐ Other
4	Access site(s) during procedure	☐ Femoral artery<br>☐ Brachial artery, ipsilateral<br>☐ Radial artery, ipsilateral<br>☐ Other
5	Was endovascular intervention performed intravascular ultrasound (IVUS) assisted?	○ Yes<br>○ No
	__Local catheter guided pharmacological thrombolysis__	
3.2	***If 'Performed endovascular procedure(s)' is equal to 'Local catheter guided pharmacological thrombolysis' answer this question:***<br>Vessel(s) treated	☐ Subclavian artery<br>☐ Axillary artery<br>☐ Brachial artery<br>☐ Radial artery<br>☐ Ulnar artery
3.3	***If 'Performed endovascular procedure(s)' is equal to 'Local catheter guided pharmacological thrombolysis' answer this question:***<br>Technique used	○ EKOS<br>○ Pulse spray catheter<br>○ Other
3.3.1	***If 'Technique used' is equal to 'Other' answer this question:***<br>Technique, other	
3.4	***If 'Performed endovascular procedure(s)' is equal to 'Local catheter guided pharmacological thrombolysis' answer this question:***<br>Agent used	○ Alteplase<br>○ Reteplase<br>○ Urokinase<br>○ Streptokinase<br>○ Other
3.4.1	***If 'Agent used' is equal to 'Other' answer this question:***<br>Agent, other	

3.5	***If 'Performed endovascular procedure(s)' is equal to 'Local catheter guided pharmacological thrombolysis' answer this question:*** Dosage per hour	
3.6	***If 'Performed endovascular procedure(s)' is equal to 'Local catheter guided pharmacological thrombolysis' answer this question:*** Treatment duration in hours	Hours
	__Pharmacomechanical thrombolysis__	
3.8	***If 'Performed endovascular procedure(s)' is equal to 'Pharmacomechanical thrombolysis' answer this question:*** Vessel(s) treated	☐ Subclavian artery<br>☐ Axillary artery<br>☐ Brachial artery<br>☐ Radial artery<br>☐ Ulnar artery
3.9	***If 'Performed endovascular procedure(s)' is equal to 'Pharmacomechanical thrombolysis' answer this question:*** Technique used	☐ Trellis<br>☐ Angiojet<br>☐ Powerpulse<br>☐ Other
3.9.1	***If 'Technique used' is equal to 'Other' answer this question:*** Technique, other	
3.10	***If 'Performed endovascular procedure(s)' is equal to 'Pharmacomechanical thrombolysis' answer this question:*** Agent used	○ Alteplase<br>○ Reteplase<br>○ Urokinase<br>○ Streptokinase<br>○ Other
3.10.1	***If 'Agent used' is equal to 'Other' answer this question:*** Agent, other	
3.11	***If 'Performed endovascular procedure(s)' is equal to 'Pharmacomechanical thrombolysis' answer this question:*** Dosage used	
	__Mechanical/aspiration thrombectomy__	

3.13	***If 'Performed endovascular procedure(s)' is equal to 'Mechanical/aspiration thrombectomy' answer this question:***<br>Vessel(s) treated	☐ Subclavian artery<br>☐ Axillary artery<br>☐ Brachial artery<br>☐ Radial artery<br>☐ Ulnar artery
3.14	***If 'Performed endovascular procedure(s)' is equal to 'Mechanical/aspiration thrombectomy' answer this question:***<br>Technique used	☐ Rotarex<br>☐ Thrombex PMT<br>☐ Cleaner<br>☐ Angiojet<br>☐ Manual suction<br>☐ Other
3.14.1	***If 'Technique used' is equal to 'Other' answer this question:***<br>Technique, other	
	__Percutaneous transluminal angioplasty__	
3.16	***If 'Performed endovascular procedure(s)' is equal to 'Percutaneus transluminal angioplasty' answer this question:***<br>Vessel(s) treated	☐ Subclavian artery<br>☐ Axillary artery<br>☐ Brachial artery<br>☐ Radial artery<br>☐ Ulnar artery
3.17	***If 'Performed endovascular procedure(s)' is equal to 'Percutaneus transluminal angioplasty' answer this question:***<br>Type of balloon used	☐ Noncompliant (high-pressure) balloon<br>☐ Semicompliant (midpressure) balloon<br>☐ Compliant (elastomeric) balloon<br>☐ Drug eluting balloon<br>☐ Cutting balloon<br>☐ Other<br>☐ Unknown
3.17.1	***If 'Type of balloon used' is equal to 'Other' answer this question:***<br>Which other type of balloon?	
3.18	***If 'Performed endovascular procedure(s)' is equal to 'Percutaneus transluminal angioplasty' answer this question:***<br>maximum diameter balloon used<br>If unknown, choose '0'	____ mm
	__Stent placement__	

3.20	***If 'Performed endovascular procedure(s)' is equal to 'Stent placement' answer this question:***<br>Vessel(s) stented	☐ Subclavian artery<br>☐ Axillary artery<br>☐ Brachial artery<br>☐ Radial artery<br>☐ Ulnar artery
3.21	***If 'Performed endovascular procedure(s)' is equal to 'Stent placement' answer this question:***<br>Stent placement because of	☐ Stenosis<br>☐ Aneurysm
3.22	***If 'Performed endovascular procedure(s)' is equal to 'Stent placement' answer this question:***<br>Number of stents inserted<br>If unknown, choose '0'	[ ] Stent(s)
3.23	***If 'Performed endovascular procedure(s)' is equal to 'Stent placement' answer this question:***<br>Maximum diameter stent used<br>If unknown, choose '0'	[ ] mm
3.24	***If 'Performed endovascular procedure(s)' is equal to 'Stent placement' answer this question:***<br>Approximately total length of stented artery/arteries<br>If unknown, choose '0'	[ ] mm
3.25	***If 'Performed endovascular procedure(s)' is equal to 'Stent placement' answer this question:***<br>Type of stent(s) used	☐ Self expandable bare metal<br>☐ Balloon expandable bare metal<br>☐ Self expandable covered stent<br>☐ Balloon expandable covered stent<br>☐ Drug eluting<br>☐ Biodegradable<br>☐ Other<br>☐ Unknown
3.25.1	***If 'Type of stent(s) used' is equal to 'Other' answer this question:***<br>Which other type of stent?	[ ]
3.26	***If 'Performed endovascular procedure(s)' is equal to 'Stent placement' answer this question:***<br>Please describe every imlanted stent in detal, including brand, type, material, diameter, length, and location (vessel)<br>Example:<br>Boston Scientific Innova, self-expandable nitinol bare metal, 6mm diameter, 80mm length, axillary artery<br>BD Covera, self-expandable nitinol ePTFE covered, 8mm diameter, 40mm length, subclavian artery.	[ ]

__'Other' endovascular intervention__

3.28	***If 'Performed endovascular procedure(s)' is equal to 'Other' answer this question:***<br>Please describe 'other' endovascular intervention in detail	
	__Intravascular ultrasound__	
5.2	***If 'Was endovascular intervention performed intravascular ultrasound (IVUS) assisted?' is equal to 'Yes' answer this question:***<br>Any relevant IVUS findings?	
6	Closure technique	☐ Manual compression<br>☐ Pressure bandage<br>☐ Assisted compression device<br>☐ Vascular closure device, Angio-Seal<br>☐ Vascular closure device, Mynx<br>☐ Vascular closure device, Perclose Proglide<br>☐ Vascular closure device, StarClose<br>☐ Other
6.1	***If 'Closure technique' is equal to 'Other' answer this question:***<br>What other closure technique	
	__Result after endovascular intervention__	
7	Were provocative maneuvers performed?	○ Yes<br>○ No
8	Result local at thoracic outlet after endovascular intervention<br>Local= at the thoracic outlet	○ Fully successfull with normal open residual arterial bed<br>○ Partially successful with residual stenosis<br>○ Partially successful , residual abnormality<br>○ Partially successful, but artery remains occluded<br>○ Unsuccessful<br>○ Worsened<br>○ Not applicable, no local disease or local disease not treated

8.1	***If 'Result local at thoracic outlet after endovascular intervention' is equal to 'Partially successful , residual abnormality' answer this question:***<br>Severity of intrinsic abnormality	◯ Mild<br>◯ Moderate<br>◯ Severe
8.2	***If 'Result local at thoracic outlet after endovascular intervention' is equal to 'Partially successful , residual abnormality' answer this question:***<br>Please describe local residual abnormality	
8.3	***If 'Result local at thoracic outlet after endovascular intervention' is equal to '' answer this question:***<br>Please elaborate why local situation worsened	
7.1	***If 'Were provocative maneuvers performed?' is equal to 'Yes' answer this question:***<br>Compression of subclavian artery with provocative maneuvres?	◯ Yes<br>◯ No
7.1.1	***If 'Compression of subclavian artery with provocative maneuvres?' is equal to 'Yes' answer this question:***<br>Degree of compression with provocative maneuvres	◯ Compression, 1-50% lumen reduction<br>◯ Compression, 51-99% lumen reduction<br>◯ 100% lumen reduction with provocative maneuvres
9	Result distal of thoracic outlet after endovascular intervention<br>Distal = distal of thoracic outlet	◯ Fully successfull with normal residual arterial bed<br>◯ Partially successfull with residual abnormality (please describe)<br>◯ Partially successful with residual stenosis at rest<br>◯ Partially successful, artery remains occluded<br>◯ Unsuccessful<br>◯ Worsened<br>◯ Not applicable, no distal disease or distal disease not treated
9.1	***If 'Result distal of thoracic outlet after endovascular intervention' is equal to 'Partially successfull with residual abnormality (please describe)' answer this question:***<br>Please describe distal residual abnormality	
9.2	***If 'Result distal of thoracic outlet after endovascular intervention' is equal to 'Partially successful with residual stenosis at rest' answer this question:***<br>Severity of residual stenosis	◯ Mild<br>◯ Moderate<br>◯ Severe

9.3	***If 'Result distal of thoracic outlet after endovascular intervention' is equal to 'Worsened' answer this question:*** Please elaborate why distal situation worsened	
10	Any other relevant remarks regarding intervention?	
	__ATTENTION: please don't press 'Close report', press 'next' instead to go to the next page and complete the arterial endovascular intervention form before returning to the eCRF__	

Form Complications

Number	Question	Answers
1	Did a complication occur?	○ Yes<br>○ No
1.1	***If 'Did a complication occur?' is equal to 'Yes' answer this question:*** Type of complication(s)	☐ Bleeding complication, thrombolysis related<br>☐ Bleeding complication, not related to thrombolysis<br>☐ Access site pseudoaneurysm<br>☐ Angioplasty site dissection<br>☐ Angioplasty site distal embolisation<br>☐ Wire perforation<br>☐ Cardiovascular complication<br>☐ Contrast related nephropathy<br>☐ Other
1.1.1	***If 'Type of complication(s)' is equal to 'Bleeding complication, thrombolysis related' answer this question:*** Bleeding complication, related to thrombolysis	☐ Mucocutaneous<br>☐ Gastrointestinal<br>☐ Genitourinary<br>☐ Broncho-pulmonary<br>☐ Musculo-skeletal and soft tissue<br>☐ Body cavity (pleural, peritoneal, etc)<br>☐ Central nervous system<br>☐ Invasive site bleeding (including access site)<br>☐ Other

1.1.1.1	***If 'Bleeding complication, related to thrombolysis' is equal to 'Other' answer this question:*** What other thrombolysis related bleeding complication?	
1.1.2	***If 'Type of complication(s)' is equal to 'Bleeding complication, thrombolysis related' answer this question:*** Please describe thrombolysis related bleeding complication in detail	
1.1.3	***If 'Type of complication(s)' is equal to 'Bleeding complication, thrombolysis related' answer this question:*** Consequence of thrombolysis related bleeding complication	☐ No consequence<br>☐ Moderate hemodynamic instability<br>☐ Severe hemodynamic instability<br>☐ End organ faillure<br>☐ Fatal bleeding, patient deceased<br>☐ Other
1.1.3.1	***If 'Consequence of thrombolysis related bleeding complication' is equal to 'Other' answer this question:*** Please describe other consequence in detail	
1.1.4	***If 'Type of complication(s)' is equal to 'Bleeding complication, thrombolysis related' answer this question:*** Treatment of thrombolysis related bleeding complication	☐ No treatment<br>☐ Red blood cell transfusion (over any routine transfusion needs)<br>☐ Intensive care unit admission<br>☐ Other
1.1.4.1	***If 'Treatment of thrombolysis related bleeding complication' is equal to 'Intensive care unit admission' answer this question:*** Number of days admitted to the intensive care unit	Day(s)
1.1.4.2	***If 'Treatment of thrombolysis related bleeding complication' is equal to 'Other' answer this question:*** Please describe other treatment(s) in detail	
1.1.5	***If 'Type of complication(s)' is equal to 'Bleeding complication, not related to thrombolysis' answer this question:*** Please describe non-thrombolysis related bleeding complication in detail	

1.1.6	***If 'Type of complication(s)' is equal to 'Bleeding complication, not related to thrombolysis' answer this question:***<br>Consequence of non-thrombolysis related bleeding complication	☐ No consequence<br>☐ Moderate hemodynamic instability<br>☐ Severe hemodynamic instability<br>☐ End organ faillure<br>☐ Fatal bleeding, patient deceased<br>☐ Other
1.1.6.1	***If 'Consequence of non-thrombolysis related bleeding complication' is equal to 'Other' answer this question:***<br>Please describe other consequence in detail	
1.1.7	***If 'Type of complication(s)' is equal to 'Bleeding complication, not related to thrombolysis' answer this question:***<br>Treatment of non-thrombolysis related bleeding complication	☐ No treatment<br>☐ Red blood cell transfusion (over any routine transfusion needs)<br>☐ Intensive care unit admission<br>☐ Other
1.1.7.1	***If 'Treatment of non-thrombolysis related bleeding complication' is equal to 'Intensive care unit admission' answer this question:***<br>Number of days admitted to the intensive care unit	Day(s)
1.1.7.2	***If 'Treatment of non-thrombolysis related bleeding complication' is equal to 'Other' answer this question:***<br>Please describe other treatment(s) in detail	
1.1.8	***If 'Type of complication(s)' is equal to 'Access site pseudoaneurysm' answer this question:***<br>Please describe pseudoaneurysm in detail, including treatment (and ICU admission if applicable)	
1.1.9	***If 'Type of complication(s)' is equal to 'Angioplasty site dissection' answer this question:***<br>Please describe angioplasty site dissection in detail, including additional treatment (including ICU admission if applicable)	
1.1.10	***If 'Type of complication(s)' is equal to 'Angioplasty site distal embolisation' answer this question:***<br>Please describe distal embolisation complication in detail, including etiology, consequences and treatment (including ICU admission)	

1.1.11	***If 'Type of complication(s)' is equal to 'Wire perforation' answer this question:*** Please describe wire perforation in detail, including additional treatment performed (and ICU admission if apliccable)	
1.1.12	***If 'Type of complication(s)' is equal to 'Cardiovascular complication' answer this question:*** Please describe cardiovascular complication in detail, this includes etiology, consequences and treatment (including ICU admission)	
1.1.13	***If 'Type of complication(s)' is equal to 'Contrast related nephropathy' answer this question:*** Please describe contrast related nephropathy in detail, including treatment, dialysis and ICU admission if applicable	
1.1.14	***If 'Type of complication(s)' is equal to 'Other' answer this question:*** Please describe 'other' complication(s) in detail, including etiology, consequences and treatment (including ICU admission)	
	__When finished with the 'arterial endovascular form', please click 'close report' to return to the eCRF. This form will remain visible and accessible in the left side of the screen.__	

Repeating Data 'Surgical intervention'

Form Surgical intervention

Number	Question	Answers
1	Date of surgical intervention	*(dd-mm-yyyy)*

2	Performed surgical intervention(s)	☐ Anterior first rib resection<br>☐ Posterior first rib resection<br>☐ Total first rib resection<br>☐ Cervical rib resection<br>☐ Second rib resection<br>☐ Partial anterior scalenectomy<br>☐ Total anterior scalenectomy<br>☐ Partial middle scalenectomy<br>☐ Total middle scalenectomy<br>☐ Partial subclavius resection<br>☐ Total subclavius resection<br>☐ Pectoralis minor division<br>☐ Partial resection of pectoralis minor<br>☐ Brachial plexus neurolysis<br>☐ Subclavian vein venolysis<br>☐ Venous reconstruction<br>☐ Embolectomy<br>☐ Arterial repair/replacement<br>☐ Arterial bypass<br>☐ Fasciotomy<br>☐ Amputation<br>☐ Other
3	TOD	
2.1	***If 'Performed surgical intervention(s)' is equal to 'Other' answer this question:***<br>Please describe 'other' surgical intervention(s)	
3.1	***If 'TOD' is equal to '1' answer this question:***<br>Surgical approach	☐ Transaxillary<br>☐ Infraclavicular<br>☐ Paraclavicular<br>☐ Supraclavicular<br>☐ Posterior<br>☐ Claviculectomy<br>☐ Clavicular rotation<br>☐ Transthoracic<br>☐ Other
3.1.1	***If 'Surgical approach' is equal to 'Other' answer this question:***<br>What other surgical approach	

3.2	***If 'TOD' is equal to '1' answer this question:***<br>Video assisted?	○ Yes<br>○ No
3.3	***If 'TOD' is equal to '1' answer this question:***<br>Robot assisted?	○ Yes<br>○ No
2.2	***If 'Performed surgical intervention(s)' is equal to 'Venous reconstruction' answer this question:***<br>Surgical venous reconstruction	☐ Repair and primary closure<br>☐ Repair, vein patch<br>☐ Repair, prosthetic patch<br>☐ Interposition, venous<br>☐ Interposition, prosthetic<br>☐ Jugular turndown<br>☐ Other
2.3	***If 'Performed surgical intervention(s)' is equal to 'Embolectomy' answer this question:***<br>Embolectomy peformed of	☐ Subclavian artery<br>☐ Axillary artery<br>☐ Brachial artery<br>☐ Radial artery<br>☐ Ulnar artery
2.4	***If 'Performed surgical intervention(s)' is equal to 'Arterial repair/replacement' answer this question:***<br>Which artery was repaired/replaced?	☐ Subclavian artery<br>☐ Axillary artery<br>☐ Brachial artery<br>☐ Radial artery<br>☐ Ulnar artery
2.5	***If 'Performed surgical intervention(s)' is equal to 'Arterial repair/replacement' answer this question:***<br>Material used, arterial repair/replacement	☐ Primary closure<br>☐ Saphenous vein<br>☐ PTFE<br>☐ Dacron<br>☐ Other
2.5.1	***If 'Material used, arterial repair/replacement' is equal to 'Other' answer this question:***<br>Other material	
2.6	***If 'Performed surgical intervention(s)' is equal to 'Arterial bypass' answer this question:***<br>Inflow vessel bypass	○ Subclavian artery, ipsilateral<br>○ Axillary artery, ipsilateral<br>○ Brachial artery, ipsilateral<br>○ Subclavian artery, contralateral<br>○ Axillary artery, contralateral<br>○ Carotid artery, ipsilateral<br>○ Other

2.6.1	***If 'Inflow vessel bypass' is equal to 'Other' answer this question:***<br>Which other inflow vessel	
2.7	***If 'Performed surgical intervention(s)' is equal to 'Arterial bypass' answer this question:***<br>Outflow vessel bypass	○ Subclavian artery<br>○ Axillary artery<br>○ Brachial artery<br>○ Radial artery<br>○ Ulnar artery
2.8	***If 'Performed surgical intervention(s)' is equal to 'Arterial bypass' answer this question:***<br>Material used, bypass	○ Venous<br>○ PTFE<br>○ Dacron<br>○ Other
2.8.1	***If 'Material used, bypass' is equal to 'Other' answer this question:***<br>Other material	
2.9	***If 'Performed surgical intervention(s)' is equal to 'Fasciotomy' answer this question:***<br>Fasciotomy performed of	☐ Hand<br>☐ Forearm<br>☐ Upperarm
2.10	***If 'Performed surgical intervention(s)' is equal to 'Amputation' answer this question:***<br>Amputation performed of<br>Please choose most proximal level of amputation	☐ Dig 1<br>☐ Dig 2<br>☐ Dig 3<br>☐ Dig 4<br>☐ Dig 5<br>☐ Hand<br>☐ Forearm<br>☐ Upperarm
3.4	***If 'TOD' is equal to '1' answer this question:***<br>Bands Intraoperative?	☐ No<br>☐ From cervical rib<br>☐ From c7 transverse<br>☐ From first rib<br>☐ Unknown
4	Any intraoperative abnormalities?	

5	Wound drain?	◯ Yes<br>◯ No
3.5	***If 'TOD' is equal to '1' answer this question:***<br>Pleural entry ?	☐ No<br>☐ Minor<br>☐ Major<br>☐ Chesttube
6	Intraoperative complication(s)	◯ Yes<br>◯ No
6.1	***If 'Intraoperative complication(s)' is equal to 'Yes' answer this question:***<br>Requiring additional therapy?	◯ Yes<br>◯ No
6.2	***If 'Intraoperative complication(s)' is equal to 'Yes' answer this question:***<br>Please describe intraoperative complication	
6.1.1	***If 'Requiring additional therapy?' is equal to 'Yes' answer this question:***<br>Please describe additional therapy for intraoperative complication (including transfusion therapy)	
7	Any post-operative complications? (<30 days)	◯ Yes<br>◯ No
7.1	***If 'Any post-operative complications? (<30 days)' is equal to 'Yes' answer this question:***<br>Which complication(s) post-operative?	☐ Pneumothorax<br>☐ Wound infection<br>☐ Hemothorax<br>☐ Phrenic nerve injury<br>☐ Long thoracic nerve injury<br>☐ Brachial plexus injury<br>☐ Chyle leak<br>☐ Other
7.1.1	***If 'Which complication(s) post-operative?' is equal to 'Other' answer this question:***<br>Please describe 'other' complication(s).	
7.2	***If 'Any post-operative complications? (<30 days)' is equal to 'Yes' answer this question:***<br>Did the complication(s) require any additional treatment?	◯ Yes<br>◯ No

7.3	***If 'Any post-operative complications? (<30 days)' is equal to 'Yes' answer this question:***<br>Complication required admission to intensive care unit?	○ Yes<br>○ No
7.1.2	***If 'Which complication(s) post-operative?' is equal to 'Pneumothorax' answer this question:***<br>Pneumothorax	☐ Small<br>☐ Medium<br>☐ Large<br>☐ Symptomatic<br>☐ Requiring chest tube
7.1.3	***If 'Which complication(s) post-operative?' is equal to 'Hemothorax' answer this question:***<br>Hemothorax	☐ Small<br>☐ Medium<br>☐ Large<br>☐ Symptomatic<br>☐ Requiring chest tube
7.1.4	***If 'Which complication(s) post-operative?' is equal to 'Wound infection' answer this question:***<br>Wound infection	☐ Minor<br>☐ Major<br>☐ Reopened/explored at bedside<br>☐ Reopened/explored under general anethesia
7.2.1	***If 'Did the complication(s) require any additional treatment?' is equal to 'Yes' answer this question:***<br>Please describe additional treatment for complication(s) in detail	
8	Hospital stay after surgery<br>Day of surgery is day 0	Days
7.4	***If 'Any post-operative complications? (<30 days)' is equal to 'Yes' answer this question:***<br>Complication required readmission to hospital? (After patient was discharged)	○ Yes<br>○ No
7.4.1	***If 'Complication required readmission to hospital? (After patient was discharged)' is equal to 'Yes' answer this question:***<br>Readmission after<br>day of surgery is day 0	Days
7.4.2	***If 'Complication required readmission to hospital? (After patient was discharged)' is equal to 'Yes' answer this question:***<br>Hospital stay after readmission<br>Day of readmission is day 0	Days

7.4.3	***If 'Complication required readmission to hospital? (After patient was discharged)' is equal to 'Yes' answer this question:***<br>Please describe reason for readmission	
7.3.1	***If 'Complication required admission to intensive care unit?' is equal to 'Yes' answer this question:***<br>Days on intesive care unit<br>Day of admission on ICU is day 1	days
7.3.2	***If 'Complication required admission to intensive care unit?' is equal to 'Yes' answer this question:***<br>Please describe reason for ICU admission	
	__When finished with the 'surgical intervention form', please click 'close report' to return to the eCRF. Choose add another if you want to add another surgical intervention form.__	

Repeating Data 'Follow-up contact or event'

Form General

Number	Question	Answers
1	Date follow-up contact or event	*(dd-mm-yyyy)*
2	Type of follow-up contact or event	☐ Schedueled outpatient visit or telephone contact<br>☐ Unschedueled outpatient visit or telephone contact<br>☐ Hosptial admission<br>☐ Other
2.1	***If 'Type of follow-up contact or event' is equal to 'Other' answer this question:***<br>Describe other contact/event	

3	Please check matters that apply to this contact or event	☐ An anamnesis/medical history was taken<br>☐ A physical exam was performed<br>☐ The upper extremity post thrombotic syndrome score was used to assess the patient<br>☐ Blood testing performed<br>☐ Diagnostic modialities performed<br>☐ Change of treatment strategy/additional treatment performed? This includes discontinuation of medication.<br>☐ Changes in health status (excluding events of the affected arm related to TOS/idiopatic UEDVT or contralteral TOS/idiopatic UEDVT), such as cardiac disease, hypercoagulable state, malignancies, surgery etc.<br>☐ Patient assesed/treated in another hospital for TOS/UEDVT<br>☐ New diagnosis of contralateral TOS or idiopatic upper extremity deep venous thrombosis<br>☐ Patient deceased<br>☐ Other
3.1	***If 'Please check matters that apply to this contact or event' is equal to 'Other' answer this question:***<br>Please describe other contact or event in detail	
	__Changes in health status__	
3.3	***If 'Please check matters that apply to this contact or event' is equal to 'Changes in health status (excluding events of the affected arm related to TOS/idiopatic UEDVT or contralteral TOS/idiopatic UEDVT), such as cardiac disease, hypercoagulable state, malignancies, surgery etc.' answer this question:***<br>What disease, condition and/or intervention?	☐ Cardiovascular disease<br>☐ Malignancy<br>☐ Thrombosis<br>☐ Surgery<br>☐ Diagnosis of hypercoagulable state<br>☐ Other
3.3.1	***If 'What disease, condition and/or intervention?' is equal to 'Cardiovascular disease' answer this question:***<br>What cardiovascular disease?	☐ Coronary artery disease<br>☐ Heart faillure<br>☐ Cardiomyopathy<br>☐ Cardiac dysrhythmias<br>☐ Atrial fibrilation/flutter<br>☐ Pacemaker<br>☐ Defibrilator (ICD)<br>☐ Cerebrovascular accident<br>☐ Peripheral vascular disease<br>☐ Aortic aneurysm<br>☐ Other

3.3.1.1	***If 'What cardiovascular disease?' is equal to 'Other' answer this question:***<br>What other cardiovascular disease?	
3.3.2	***If 'What disease, condition and/or intervention?' is equal to 'Malignancy' answer this question:***<br>What type of malignancy?	☐ Breast cancer<br>☐ Colorectal cancer<br>☐ Prostate cancer<br>☐ Lung cancer<br>☐ Melanoma<br>☐ Basal cell carcinoma<br>☐ Uterine cancer<br>☐ Bladder cancer<br>☐ Thyroid cancer<br>☐ Lymfoma<br>☐ Other
3.3.2.1	***If 'What type of malignancy?' is equal to 'Other' answer this question:***<br>What other malignancy?	
3.3.3	***If 'What disease, condition and/or intervention?' is equal to 'Thrombosis' answer this question:***<br>What type of thrombosis?	☐ Deep venous thrombosis lower extremity<br>☐ Pulmonary embolus<br>☐ Other
3.3.4	***If 'What disease, condition and/or intervention?' is equal to 'Surgery' answer this question:***<br>What type of surgery?	
3.3.3.1	***If 'What type of thrombosis?' is equal to 'Other' answer this question:***<br>What other type of thrombosis?	

3.3.5	***If 'What disease, condition and/or intervention?' is equal to 'Diagnosis of hypercoagulable state' answer this question:***<br>What hypercoagulable state?	☐ Factor V Leiden<br>☐ Prothrombin G20210A<br>☐ High factor VIII<br>☐ Factor VII mutation<br>☐ Factor II mutation<br>☐ Protein C deficiency<br>☐ Protein S deficiency<br>☐ Antithrombin deficiency<br>☐ Antiphospholipid antibodies<br>☐ Lupus anticoagulant<br>☐ Anticardiolipin IgG<br>☐ Anticardiolipin IgM<br>☐ Anti-beta-2 glycoproteine IgG<br>☐ Anti-beta-2 glycoproteine IgM<br>☐ Plasminogeen activator inhibitor-1<br>☐ Other
3.3.5.1	***If 'What hypercoagulable state?' is equal to 'Other' answer this question:***<br>What other hypercoagulable state?	
3.3.6	***If 'What disease, condition and/or intervention?' is equal to 'Other' answer this question:***<br>What other disease, condition and/or intervention?	
3.4	***If 'Please check matters that apply to this contact or event' is equal to 'Patient assesed/treated in another hospital for TOS/UEDVT' answer this question:***<br>Please state the reason for assesment and/or treatment in other hospital	
	__Please first complete this follow-up form with the information provided by the other hospital, such as physical exam, diagnostics or treatment. If patient also visited your hospital, please provide this information in another follow-up form.__	
	__In order to collect specific data per extremity, a patient with bilateral TOS/ idiopatic UEDVT is assigned two studynumbers, one for each extremity. If this is the first eCRF for this patient, please open a new eCRF for the other extremity..__	
3.7	***If 'Please check matters that apply to this contact or event' is equal to 'New diagnosis of contralateral TOS or idiopatic upper extremity deep venous thrombosis' answer this question:***<br>Please provide the corresponding study number of the contralateral arm	

3.8	***If 'Please check matters that apply to this contact or event' is equal to 'Patient deceased' answer this question:***<br>Please describe cause of death	
	__ATTENTION: please don't press 'Close report', press 'next' instead to go to the next page and complete the follow-up form before returning to the eCRF__	

Form Anamnesis

Number	Question	Answers
1	calc anamnesis	
	No anamnesis taken, please continue.	
1.2	***If 'calc anamnesis' is equal to '1' answer this question:***<br>Compared to last contact, patient has	○ No complaints or symptoms<br>○ Reduced symptoms<br>○ Persisting symptoms<br>○ New symptoms<br>○ Worsened symptoms
1.2.1	***If 'Compared to last contact, patient has' is equal to 'New symptoms' answer this question:***<br>What are the new symptoms?	☐ Pain<br>☐ Swelling<br>☐ Tingling<br>☐ Numbness<br>☐ Heavyness<br>☐ Loss of function<br>☐ Loss of strength/weakness<br>☐ Discoloration-white<br>☐ Discoloration-red<br>☐ Discoloration-blue<br>☐ Coldness<br>☐ Temperature sensitivity<br>☐ Vein collaterals<br>☐ Ulceration, gangrene or tissue loss<br>☐ Microemboli<br>☐ Unknown<br>☐ Other
1.2.1.1	***If 'What are the new symptoms?' is equal to 'Other' answer this question:***<br>Specify other symptoms	

1.2.1.2	***If 'What are the new symptoms?' is equal to 'Pain' answer this question:*** Location pain	☐ Neck<br>☐ Chest<br>☐ Shoulder<br>☐ Whole arm<br>☐ Upper arm<br>☐ Forearm<br>☐ Hand, without all digiti<br>☐ Hand, including all digiti<br>☐ Specific dig(iti)<br>☐ Unclear
1.2.1.2.1	***If 'Location pain' is equal to 'Specific dig(iti)' answer this question:*** Pain in which specific dig(iti)?	☐ Dig 1<br>☐ Dig 2<br>☐ Dig 3<br>☐ Dig 4<br>☐ Dig 5
1.2.1.3	***If 'What are the new symptoms?' is equal to 'Numbness' answer this question:*** Location numbness	☐ Neck<br>☐ Chest<br>☐ Shoulder<br>☐ Upper arm<br>☐ Forearm<br>☐ Hand, dig 1-3<br>☐ Hand, dig 4-5<br>☐ Unclear
1.2.1.4	***If 'What are the new symptoms?' is equal to 'Tingling' answer this question:*** Location tingling	☐ Neck<br>☐ Chest<br>☐ Shoulder<br>☐ Upper arm<br>☐ Forearm<br>☐ Hand, dig 1-3<br>☐ Hand, dig 4-5<br>☐ Unclear
1.2.1.5	***If 'What are the new symptoms?' is equal to 'Loss of strength/weakness' answer this question:*** Location weakness	☐ Neck<br>☐ Chest<br>☐ Shoulder<br>☐ Upper arm<br>☐ Forearm<br>☐ Hand, dig 1-3<br>☐ Hand, dig 4-5<br>☐ Unclear

1.2.1.6	***If 'What are the new symptoms?' is equal to 'Microemboli' answer this question:***<br>Please describe microemboli	
1.2.2	***If 'Compared to last contact, patient has' is not equal to 'No complaints or symptoms' answer this question:***<br>Nature of complaints	☐ Continuously<br>☐ Provoked by excercise<br>☐ Provoked by work<br>☐ Posture dependant<br>☐ Nocturnal<br>☐ Unclear
1.2.2.1	***If 'Nature of complaints' is equal to 'Provoked by excercise' answer this question:***<br>Specify excercise	
1.2.2.2	***If 'Nature of complaints' is equal to 'Provoked by work' answer this question:***<br>Able to work?	○ Yes<br>○ No<br>○ Partially<br>○ Unknown
1.2.2.3	***If 'Nature of complaints' is equal to 'Posture dependant' answer this question:***<br>Specify posture	
1.2.3	***If 'Compared to last contact, patient has' is equal to 'New symptoms' answer this question:***<br>Date onset of new symptoms<br>If precise date is unknown:<br>5-2020 -> 01-05-2020<br>2020 -> 01-01-2020	__ __ __ *(dd-mm-yyyy)*
1.2.4	***If 'Compared to last contact, patient has' is equal to 'New symptoms' answer this question:***<br>Acute or gradual onset of new symptoms?	○ Acute<br>○ Gradual<br>○ Gradual with acute worsening<br>○ Unclear
2	Calc, acute onset	
2.1	***If 'Calc, acute onset' is equal to '1' answer this question:***<br>Any (unusual) activities leading to start of the new symptoms?	

1.2.5	***If 'Compared to last contact, patient has' is equal to 'New symptoms' answer this question:*** Any (unusual) activities in the days or weeks before start of new symptoms potentially connected?	
	__ATTENTION: please don't press 'Close report', press 'next' instead to go to the next page and complete the follow-up form before returning to the eCRF__	

Form Physical exam

Number	Question	Answers
1	calc FU physical	
	No physical exam performed, please continue.	
1.2	***If 'calc FU physical' is equal to '1' answer this question:*** What was tested/examined?	☐ Inspection affected upper extremity<br>☐ Posture<br>☐ Arm use<br>☐ nTOS testing (e.g. palpation supraclavicular/pec minor space)<br>☐ Neurological exam<br>☐ Vascular exam (pulsations, blood pressure, etc)<br>☐ Specific TOS tests (e.g. Roos test, military brace)<br>☐ Contralateral upper extremity<br>☐ Other tests/examinations or additional remarks
1.2.1	***If 'What was tested/examined?' is equal to 'Inspection affected upper extremity' answer this question:*** Inspection affected upper extremity	☐ No abnormalities<br>☐ Swelling<br>☐ Athrophy<br>☐ Visible chest wall collaterals<br>☐ Discoloration white<br>☐ Discoloration red<br>☐ Discoloration blue<br>☐ Decreased temperature<br>☐ Microembolisation<br>☐ Ulcerations, gangrene or tissue loss<br>☐ Other
1.2.1.1	***If 'Inspection affected upper extremity' is equal to 'Other' answer this question:*** Inspection, other	

2	Calc, discoloration	
2.1	***If 'Calc, discoloration' is equal to '1' answer this question:***<br>Discoloration of	☐ Neck<br>☐ Chest<br>☐ Shoulder<br>☐ Whole arm<br>☐ Upper arm<br>☐ Forearm<br>☐ Hand, without all digiti<br>☐ Hand, including all digiti<br>☐ Specific dig(iti)<br>☐ Unclear
2.1.1	***If 'Discoloration of' is equal to 'Specific dig(iti)' answer this question:***<br>Which dig(iti)	☐ Dig 1<br>☐ Dig 2<br>☐ Dig 3<br>☐ Dig 4<br>☐ Dig 5
1.2.1.2	***If 'Inspection affected upper extremity' is equal to 'Athrophy' answer this question:***<br>Atrophy	☐ Shoulder<br>☐ Neck<br>☐ Chest<br>☐ Upper arm<br>☐ Forearm<br>☐ Hand, dig 1-3<br>☐ Hand, dig 4-5
1.2.1.3	***If 'Inspection affected upper extremity' is equal to 'Microembolisation' answer this question:***<br>Please describe microemboli, including location	
1.2.1.4	***If 'Inspection affected upper extremity' is equal to 'Ulcerations, gangrene or tissue loss' answer this question:***<br>Ulcerations, gangrene or tissue loss of	☐ Dig 1<br>☐ Dig 2<br>☐ Dig 3<br>☐ Dig 4<br>☐ Dig 5<br>☐ Hand<br>☐ Forearm<br>☐ Upperarm

1.2.2	***If 'What was tested/examined?' is equal to 'nTOS testing (e.g. palpation supraclavicular/pec minor space)' answer this question:***<br>Neck exam	☐ Normal<br>☐ Swelling<br>☐ Tightness<br>☐ Asymetry<br>☐ Possible cervical rib<br>☐ Not performed
1.2.3	***If 'What was tested/examined?' is equal to 'nTOS testing (e.g. palpation supraclavicular/pec minor space)' answer this question:***<br>Supraclavicular tenderness with palpation	☐ Nontender<br>☐ Mild<br>☐ Moderate<br>☐ Severe<br>☐ Reproduces distal symptoms<br>☐ Not performed
1.2.4	***If 'What was tested/examined?' is equal to 'nTOS testing (e.g. palpation supraclavicular/pec minor space)' answer this question:***<br>Pectoralis minor space tenderness with palpation	☐ Nontender<br>☐ Mild<br>☐ Moderate<br>☐ Severe<br>☐ Reproduces distal symptoms<br>☐ Not performed
1.2.5	***If 'What was tested/examined?' is equal to 'nTOS testing (e.g. palpation supraclavicular/pec minor space)' answer this question:***<br>Axillary tenderness with palpation	☐ Nontender<br>☐ Mild<br>☐ Moderate<br>☐ Severe<br>☐ Reproduces distal symptoms<br>☐ Not performed
1.2.6	***If 'What was tested/examined?' is equal to 'Neurological exam' answer this question:***<br>Sensiblity, general	○ Normal<br>○ Decreased sensibility<br>○ Absent sensibility<br>○ Not tested
1.2.7	***If 'What was tested/examined?' is equal to 'Neurological exam' answer this question:***<br>Muscle strength, general	○ Normal<br>○ Decreased strength, mild<br>○ Decreased strength, severe<br>○ Absent contractions<br>○ Not tested
1.2.8	***If 'What was tested/examined?' is equal to 'Neurological exam' answer this question:***<br>Specific nerves tested?	☐ No<br>☐ Median nerve<br>☐ Ulnar nerve<br>☐ radial nerve

1.2.8.1	***If 'Specific nerves tested?' is equal to 'Median nerve' answer this question:***<br>Median nerve	☐ Normal sensibility and strength<br>☐ Normal sensibility<br>☐ Decresed sensibility<br>☐ Absent sensibility<br>☐ Normal strength<br>☐ Descreased strength, mild<br>☐ Descreased strength, severe<br>☐ No muscle contractions
1.2.8.2	***If 'Specific nerves tested?' is equal to 'Ulnar nerve' answer this question:***<br>Ulnar nerve	☐ Normal sensibility and strength<br>☐ Normal sensibility<br>☐ Decresed sensibility<br>☐ Absent sensibility<br>☐ Normal strength<br>☐ Descreased strength, mild<br>☐ Descreased strength, severe<br>☐ No muscle contractions
1.2.8.3	***If 'Specific nerves tested?' is equal to 'radial nerve' answer this question:***<br>Radial nerve	☐ Normal sensibility and strength<br>☐ Normal sensibility<br>☐ Decresed sensibility<br>☐ Absent sensibility<br>☐ Normal strength<br>☐ Descreased strength, mild<br>☐ Descreased strength, severe<br>☐ No muscle contractions
1.2.9	***If 'What was tested/examined?' is equal to 'Neurological exam' answer this question:***<br>Tinel's sign present?	○ Yes<br>○ No<br>○ Not tested
1.2.9.1	***If 'Tinel's sign present?' is equal to 'Yes' answer this question:***<br>Tinel's sign positive at	☐ Wrist<br>☐ Cubital<br>☐ Plexus
1.2.10	***If 'What was tested/examined?' is equal to 'Vascular exam (pulsations, blood pressure, etc)' answer this question:***<br>Vascular exam in rest performed? Including pulsations, blood pressure measurements and capillary refill.	○ Yes<br>○ No

1.2.11	***If 'What was tested/examined?' is equal to 'Vascular exam (pulsations, blood pressure, etc)' answer this question:***<br>Which arteries were tested?	☐ Subclavian artery<br>☐ Axillary artery<br>☐ Brachial artery<br>☐ Radial artery<br>☐ Ulnar artery<br>☐ None
1.2.11.1	***If 'Which arteries were tested?' is equal to 'Subclavian artery' answer this question:***<br>Subclavian artery	☐ Normal pulsations<br>☐ Weak pulsations<br>☐ Dopler signals only<br>☐ Absent pulsations<br>☐ Palpabel pulsating mass present (aneurysm?)<br>☐ Bruit/thrill present
1.2.11.2	***If 'Which arteries were tested?' is equal to 'Axillary artery' answer this question:***<br>Axillary artery	☐ Normal pulsations<br>☐ Weak pulsations<br>☐ Dopler signals only<br>☐ Absent pulsations<br>☐ Palpabel pulsating mass present (aneurysm?)<br>☐ Bruit/thrill present
1.2.11.3	***If 'Which arteries were tested?' is equal to 'Brachial artery' answer this question:***<br>Brachial artery	☐ Normal pulsations<br>☐ Weak pulsations<br>☐ Dopler signals only<br>☐ Absent pulsations<br>☐ Palpabel pulsating mass present (aneurysm?)<br>☐ Bruit/thrill present
1.2.11.4	***If 'Which arteries were tested?' is equal to 'Radial artery' answer this question:***<br>Radial artery	☐ Normal pulsations<br>☐ Weak pulsations<br>☐ Dopler signals only<br>☐ Absent pulsations<br>☐ Palpabel pulsating mass present (aneurysm?)<br>☐ Bruit/thrill present
1.2.11.5	***If 'Which arteries were tested?' is equal to 'Ulnar artery' answer this question:***<br>Ulnar artery	☐ Normal pulsations<br>☐ Weak pulsations<br>☐ Dopler signals only<br>☐ Absent pulsations<br>☐ Palpabel pulsating mass present (aneurysm?)<br>☐ Bruit/thrill present

1.2.12	***If 'What was tested/examined?' is equal to 'Vascular exam (pulsations, blood pressure, etc)' answer this question:***<br>Blood pressure difference present between both upper extremities	◯ Yes, BP of affected arm is lower<br>◯ Yes, BP of affected arm is higher<br>◯ No<br>◯ Not tested
1.2.12.1	***If 'Blood pressure difference present between both upper extremities' is equal to 'Yes, BP of affected arm is lower' answer this question:***<br>Systolic blood pressure difference	[ ] mmHG
1.2.13	***If 'What was tested/examined?' is equal to 'Vascular exam (pulsations, blood pressure, etc)' answer this question:***<br>Capillary refill	◯ Not tested<br>◯ Normal<br>◯ Prolonged, <5sec<br>◯ Prolonged >5sec
1.2.14	***If 'What was tested/examined?' is equal to 'Specific TOS tests (e.g. Roos test, military brace)' answer this question:***<br>Which TOS tests?	☐ Wright's test<br>☐ Adson's test<br>☐ Costoclavicular / Military brace / Eden's test<br>☐ Upper Limb Tension Tests (ULTT's) / Elvey test<br>☐ Roos test / Elevated arm stress test
1.2.14.1	***If 'Which TOS tests?' is equal to 'Wright's test' answer this question:***<br>Wright's test	◯ Negative<br>◯ Positive, loss of radial pulse<br>◯ Positive, reproduction of patients symptoms<br>◯ Positive, loss of radial pulse AND reproduction of patients symptoms
1.2.14.2	***If 'Which TOS tests?' is equal to 'Adson's test' answer this question:***<br>Adson's test	◯ Negative<br>◯ Positive, marked decrease of radial pulse<br>◯ Positive, loss of radial pulse
1.2.14.3	***If 'Which TOS tests?' is equal to 'Costoclavicular / Military brace / Eden's test' answer this question:***<br>Costoclavicular / Military Brace / Eden’s Test	◯ Negative<br>◯ Positive, marked decrease of radial pulse<br>◯ Positive, loss of radial pulse<br>◯ Positive, reproduction of patients symptoms<br>◯ Positive, decrease/loss of radial pulse AND reproduction of patients symptoms
1.2.14.4	***If 'Which TOS tests?' is equal to 'Upper Limb Tension Tests (ULTT's) / Elvey test' answer this question:***<br>Upper Limb Tension Tests (ULTT's) / Elvey test	◯ Negative<br>◯ Positive, reproduction of patients symptoms
1.2.14.5	***If 'Which TOS tests?' is equal to 'Roos test / Elevated arm stress test' answer this question:***<br>Roos test / elevated arm stress test	◯ Negative<br>◯ Positive, reproduction of patients symptoms<br>◯ Positive, not able to complete test

3	calc roos positive	
3.1	***If 'calc roos positive' is equal to '1' answer this question:*** First symptoms after	[ ] seconds
1.2.14.5.1	***If 'Roos test / elevated arm stress test' is equal to 'Positive, not able to complete test' answer this question:*** Cannot continue after	[ ] seconds
1.2.15	***If 'What was tested/examined?' is equal to 'Other tests/examinations or additional remarks' answer this question:*** Please describe any other tests/examinations or any remarks on the physical exam in detail	[ ]
	__ATTENTION: please don't press 'Close report', press 'next' instead to go to the next page and complete the follow-up form before returning to the eCRF__	

Form Upper extremity PTS score

Number	Question	Answers
1	Calc PTS UE score	
	The upper extremity PTS score was not used, please continue	
	__Symptoms of post thrombotic syndrome__	
1.3	***If 'Calc PTS UE score' is equal to '1' answer this question:*** Edema/swelling of the arm	○ Absent (0 points)<br>○ Mild (1 point)<br>○ Moderate (2 points)<br>○ Severe (3 points)
1.4	***If 'Calc PTS UE score' is equal to '1' answer this question:*** Heavy feeling of the arm	○ Absent (0 points)<br>○ Mild (1 point)<br>○ Moderate (2 points)<br>○ Severe (3 points)
1.5	***If 'Calc PTS UE score' is equal to '1' answer this question:*** Fatigue on using arm	○ Absent (0 points)<br>○ Mild (1 point)<br>○ Moderate (2 points)<br>○ Severe (3 points)
1.6	***If 'Calc PTS UE score' is equal to '1' answer this question:*** Pain (chronic or during specific excercise)	○ Absent (0 points)<br>○ Mild (1 point)<br>○ Moderate (2 points)<br>○ Severe (3 points)

1.7	***If 'Calc PTS UE score' is equal to '1' answer this question:***<br>Functional limitations arm	◯ Absent (0 points)<br>◯ Mild (1 point)<br>◯ Moderate (2 points)<br>◯ Severe (3 points)
	__Clinical signs of post thrombotic syndrome__	
1.9	***If 'Calc PTS UE score' is equal to '1' answer this question:***<br>Swelling arm measured by circumference of upper and lower arm versus contralateral side	◯ Absent (0 points)<br>◯ Mild (1 point)<br>◯ Moderate (2 points)<br>◯ Severe (3 points)
1.10	***If 'Calc PTS UE score' is equal to '1' answer this question:***<br>Discoloration of arm/hand/fingers (red/white/cyanotic) in rest	◯ Absent (0 points)<br>◯ Mild (1 point)<br>◯ Moderate (2 points)<br>◯ Severe (3 points)
1.11	***If 'Calc PTS UE score' is equal to '1' answer this question:***<br>Collateralization or collateral veins around shoulder/torso/breast	◯ Absent (0 points)<br>◯ Mild (1 point)<br>◯ Moderate (2 points)<br>◯ Severe (3 points)
1.12	***If 'Calc PTS UE score' is equal to '1' answer this question:***<br>Total signs and symptoms score (max 24)	
	__ATTENTION: please don't press 'Close report', press 'next' instead to go to the next page and complete the follow-up form before returning to the eCRF__	

Form Laboratory

Number	Question	Answers
1	Calc FU laboratory	
	No blood tests performed, please continue	
1.2	***If 'Calc FU laboratory' is equal to '1' answer this question:***<br>Which laboratory test(s)	☐ D-dimer<br>☐ Creatinine<br>☐ Hypercoagulability testing<br>☐ VerifyNow assay<br>☐ CYP2C19 genetic testing<br>☐ Other

1.2.1	***If 'Which laboratory test(s)' is equal to 'D-dimer' answer this question:***<br>Date D-dimer tested *Warning shown if field's value is larger than NOW: 'Date cannot be in the future'*	☐ ☐ ☐ *(dd-mm-yyyy)*
1.2.2	***If 'Which laboratory test(s)' is equal to 'D-dimer' answer this question:***<br>D-dimer level (in mg/L)	☐ mg/L
1.2.3	***If 'Which laboratory test(s)' is equal to 'Creatinine' answer this question:***<br>Creatinine level, reported in µmol/l<br>To convert mg/dl to µmol/l, multiply by 88,4 . Please round up the outcome to the nearest whole number.<br>Example: Creatinine level 0.90 mg/dl<br>0.90 x 88,4 = 79,56<br>Creatinine level 80 mg/dl	☐ µmol/l
1.2.4	***If 'Which laboratory test(s)' is equal to 'Hypercoagulability testing' answer this question:***<br>Which hypercoagulability tests performed?	☐ Prothrombin time (PT)<br>☐ Activated partial thromboplastin time (aPTT)<br>☐ Fibronogen level<br>☐ Anticardiolipin IgG<br>☐ Anticardiolipin IgM<br>☐ Beta-2 glycoprotein I IgG<br>☐ Beta-2 glycoprotein I IgM<br>☐ Lupus anticoagulans<br>☐ Functional protein C assay<br>☐ Antigenic protein C assay<br>☐ Functional protein S assay<br>☐ Antigenic protein S assay<br>☐ Functional antithrombin assay<br>☐ Antigenic antithrombin assay<br>☐ Activated protein C resistance testing<br>☐ Factor V Leiden mutation<br>☐ Prothrombin G20210A gene mutation<br>☐ Other test(s)
1.2.4.1	***If 'Which hypercoagulability tests performed?' is equal to 'Prothrombin time (PT)' answer this question:***<br>Prothrombin time (PT)<br>If PT was not tested, choose 0	☐ seconds
1.2.4.2	***If 'Which hypercoagulability tests performed?' is equal to 'Activated partial thromboplastin time (aPTT)' answer this question:***<br>Activated partial thromboplastin time (aPTT)<br>If aPPT was not tested, choose 0	☐ seconds

1.2.4.3	***If 'Which hypercoagulability tests performed?' is equal to 'Fibronogen level' answer this question:***<br>Fibronogen level<br>If fibrinogen level was not tested, choose 0	[ ] g/L
1.2.4.4	***If 'Which hypercoagulability tests performed?' is equal to 'Anticardiolipin IgG' answer this question:***<br>Anticardiolipin IgG	○ Positive<br>○ Negative
1.2.4.5	***If 'Which hypercoagulability tests performed?' is equal to 'Anticardiolipin IgM' answer this question:***<br>Anticardiolipin IgM	○ Positive<br>○ Negative
1.2.4.6	***If 'Which hypercoagulability tests performed?' is equal to 'Beta-2 glycoprotein I IgG' answer this question:***<br>Beta-2 glycoprotein I IgG	○ Positive<br>○ Negative
1.2.4.7	***If 'Which hypercoagulability tests performed?' is equal to 'Anticardiolipin IgM' answer this question:***<br>Beta-2 glycoprotein I IgM	○ Positive<br>○ Negative
1.2.4.8	***If 'Which hypercoagulability tests performed?' is equal to 'Lupus anticoagulans' answer this question:***<br>Lupus anticoagulans	○ Positive<br>○ Negative
1.2.4.9	***If 'Which hypercoagulability tests performed?' is equal to 'Functional protein C assay' answer this question:***<br>Functional protein C assay	○ Normal<br>○ Decreased
1.2.4.10	***If 'Which hypercoagulability tests performed?' is equal to 'Antigenic protein C assay' answer this question:***<br>Antigenic protein C assay	○ Normal<br>○ Protein defect type I<br>○ Protein defect type II
1.2.4.11	***If 'Which hypercoagulability tests performed?' is equal to 'Functional protein S assay' answer this question:***<br>Functional protein S assay	○ Normal<br>○ Decreased
1.2.4.12	***If 'Which hypercoagulability tests performed?' is equal to 'Antigenic protein S assay' answer this question:***<br>Antigenic protein S assay	○ Normal<br>○ Protein defect type I<br>○ Protein defect type II<br>○ Protein defect type III
1.2.4.13	***If 'Which hypercoagulability tests performed?' is equal to 'Functional antithrombin assay' answer this question:***<br>Functional antithrombin assay	○ Normal<br>○ Decreased
1.2.4.14	***If 'Which hypercoagulability tests performed?' is equal to 'Antigenic antithrombin assay' answer this question:***<br>Antigenic antithrombin assay	○ Normal<br>○ Antithrombin deficiency type I<br>○ Antithrombin deficiency type II

1.2.4.15	***If 'Which hypercoagulability tests performed?' is equal to 'Activated protein C resistance testing' answer this question:*** Activated protein C resistance testing	◯ Normal<br>◯ Protein C resistance
1.2.4.16	***If 'Which hypercoagulability tests performed?' is equal to 'Factor V Leiden mutation' answer this question:*** Factor V Leiden mutation	◯ Normal<br>◯ Factor V Leiden mutation present
1.2.4.17	***If 'Which hypercoagulability tests performed?' is equal to 'Prothrombin G20210A gene mutation' answer this question:*** Prothrombin G20210A gene mutation	◯ Normal<br>◯ Prothrombin G20210A gene mutation present
1.2.4.18	***If 'Which hypercoagulability tests performed?' is equal to 'Other test(s)' answer this question:*** Please describe other tests in detail	
1.2.5	***If 'Which laboratory test(s)' is equal to 'VerifyNow assay' answer this question:*** Which VerifyNow test?	☐ VerifyNow P2Y12 assay<br>☐ VerifyNow Aspirin assay
1.2.5.1	***If 'Which VerifyNow test?' is equal to 'VerifyNow P2Y12 assay' answer this question:*** VerifyNow P2Y12 assay baseline	PRU
1.2.5.2	***If 'Which VerifyNow test?' is equal to 'VerifyNow P2Y12 assay' answer this question:*** VerifyNow P2Y12 assay Post-Treatment	PRU
1.2.5.3	***If 'Which VerifyNow test?' is equal to 'VerifyNow Aspirin assay' answer this question:*** VerifyNow Aspirin assay baseline	ARU
1.2.5.4	***If 'Which VerifyNow test?' is equal to 'VerifyNow Aspirin assay' answer this question:*** VerifyNow Aspirin assay Post-Treatment	ARU
1.2.6	***If 'Which laboratory test(s)' is equal to 'CYP2C19 genetic testing' answer this question:*** Outcome CYP2C19 testing	☐ Normal<br>☐ Decreased CYP2C19 activity<br>☐ Increased CYP2C19 activity
1.2.7	***If 'Which laboratory test(s)' is equal to 'Other' answer this question:*** Describe other laboratory test(s) in detail	
	__ATTENTION: please don't press 'Close report', press 'next' instead to go to the next page and complete the follow-up form before returning to the eCRF__	

Form Diagnostic modalities I

Number	Question	Answers
1	Calc diagnostics	
	No diagnostic modalities used, please continue	
1.2	***If 'Calc diagnostics' is equal to '1' answer this question:*** Performed diagnostic modalities	☐ Electromyography<br>☐ Finger photoplethysmography (PPG) or dedicated TOS PPG<br>☐ Local injection<br>☐ Ultrasound<br>☐ Duplex ultrasound<br>☐ Chest radiograph or cervical spine series<br>☐ CT<br>☐ MRI<br>☐ Venography (with or without IVUS)<br>☐ Arteriography (with or without IVUS)<br>☐ Other
1.2.1	***If 'Performed diagnostic modalities' is equal to 'Electromyography' answer this question:*** Date EMG	*(dd-mm-yyyy)*
1.2.2	***If 'Performed diagnostic modalities' is equal to 'Electromyography' answer this question:*** Please describe rationale to perform EMG	
1.2.3	***If 'Performed diagnostic modalities' is equal to 'Electromyography' answer this question:*** C8 nerve root stimulation velocity<br>If this was not investigated, please type '0'	m/sec
1.2.4	***If 'Performed diagnostic modalities' is equal to 'Electromyography' answer this question:*** Medial antebrachial cutaneous nerve sensory neural action potential (SNAP) amplitude<br>If this was nog investigated, please type '0'	microvolts
1.2.5	***If 'Performed diagnostic modalities' is equal to 'Electromyography' answer this question:*** Medial antebrachial cutaneous nerve sensory neural action potential (SNAP) latency<br>If this was not investigated, please type '0'	miliseconds

1.2.6	***If 'Performed diagnostic modalities' is equal to 'Electromyography' answer this question:***<br>Any other relevant EMG findings?	
1.2.7	***If 'Performed diagnostic modalities' is equal to 'Finger photoplethysmography (PPG) or dedicated TOS PPG' answer this question:***<br>Date finger photoplethysmography (PPG)	*(dd-mm-yyyy)*
1.2.8	***If 'Performed diagnostic modalities' is equal to 'Finger photoplethysmography (PPG) or dedicated TOS PPG' answer this question:***<br>Finger PPG was performed	☐ At rest<br>☐ Dedicated TOS PPG, right side<br>☐ Dedicated TOS PPG, left side
1.2.8.1	***If 'Finger PPG was performed' is equal to 'At rest' answer this question:***<br>finger PPG at rest	**Signal**<br>Dig 1 ---<br>Dig 2 ---<br>Dig 3 ---<br>Dig 4 ---<br>dig 5 ---
1.2.8.2	***If 'Finger PPG was performed' is equal to 'Dedicated TOS PPG, right side' answer this question:***<br>Dedicated TOS PPG, right side	**Signal**<br>In rest ---<br>Costoclavicular maneuvre ---<br>Arms 90° abducted in coronal plain ---<br>Arms 180° abducted /Wrights position ---<br>Elevated arms stress test/Roos test ---<br>Allen test ---<br>Adson test ---<br>Arms 90° abducted in sagital plain ---<br>Symptomatic position ---<br>Other, please describe position and signal
1.2.8.3	***If 'Finger PPG was performed' is equal to 'Dedicated TOS PPG, left side' answer this question:***<br>Dedicated TOS PPG, left side	**Signal**<br>In rest ---<br>Costoclavicular maneuvre ---<br>Arms 90° abducted in coronal plain ---<br>Arms 180° abducted /Wrights position ---<br>Elevated arms stress test/Roos test ---<br>Allen test ---<br>Adson test ---<br>Arms 90° abducted in sagital plain ---<br>Symptomatic position ---<br>Other, please describe position and signal

1.2.9	***If 'Performed diagnostic modalities' is equal to 'Local injection' answer this question:***<br>Date local injection	___ ___ ___ *(dd-mm-yyyy)*
1.2.10	***If 'Performed diagnostic modalities' is equal to 'Local injection' answer this question:***<br>Which agent was injected	☐ Botulinium toxin<br>☐ Steroids<br>☐ Local anestheticum<br>☐ Other<br>☐ Unknown
1.2.10.1	***If 'Which agent was injected' is equal to 'Other' answer this question:***<br>Which other agent	
1.2.11	***If 'Performed diagnostic modalities' is equal to 'Local injection' answer this question:***<br>Dosage of agent	
1.2.12	***If 'Performed diagnostic modalities' is equal to 'Local injection' answer this question:***<br>Which structure was injected?	☐ Anterior scalene muscle<br>☐ Middle scalene muscle<br>☐ Subclavius muscle<br>☐ Pectoralis minor<br>☐ Other<br>☐ Unknown
1.2.12.1	***If 'Which structure was injected?' is equal to 'Other' answer this question:***<br>Which other structure	
1.2.13	***If 'Performed diagnostic modalities' is equal to 'Local injection' answer this question:***<br>Which technique was used?	☐ Landmarks<br>☐ EMG guidance<br>☐ Fluoroscopic guidande<br>☐ Ultrasound<br>☐ CT<br>☐ MRI<br>☐ Other<br>☐ Unknown
1.2.13.1	***If 'Which technique was used?' is equal to 'Other' answer this question:***<br>Which other technique	

1.2.14	***If 'Performed diagnostic modalities' is equal to 'Local injection' answer this question:***<br>Symptom relief after injection?	◯ Yes<br>◯ Partial<br>◯ No
1.2.14.1	***If 'Symptom relief after injection?' is not equal to 'No' answer this question:***<br>Duration of symptom relief	
1.2.15	***If 'Performed diagnostic modalities' is equal to 'Ultrasound' answer this question:***<br>Date ultrasound	*(dd-mm-yyyy)*
1.2.16	***If 'Performed diagnostic modalities' is equal to 'Ultrasound' answer this question:***<br>Please describe rationale for performing ultrasound	
1.2.17	***If 'Performed diagnostic modalities' is equal to 'Ultrasound' answer this question:***<br>Ultrasound normal or abnormal	◯ Normal<br>◯ Abnormal
1.2.17.1	***If 'Ultrasound normal or abnormal' is equal to 'Abnormal' answer this question:***<br>Describe ultrasound findings	
1.2.18	***If 'Performed diagnostic modalities' is equal to 'Duplex ultrasound' answer this question:***<br>Date duplex ultrasound	*(dd-mm-yyyy)*
1.2.19	***If 'Performed diagnostic modalities' is equal to 'Duplex ultrasound' answer this question:***<br>Was duplex performed with provocative maneuvres?	◯ Yes<br>◯ No
1.2.20	***If 'Performed diagnostic modalities' is equal to 'Duplex ultrasound' answer this question:***<br>Duplex performed	◯ Arterial<br>◯ Venous<br>◯ Arterial & venous
1.2.20.1	***If 'Duplex performed' is not equal to 'Arterial' answer this question:***<br>Vein patency, in rest	☐ Patent<br>☐ Stenotic<br>☐ Occluded

1.2.20.1.1	***If 'Vein patency, in rest' is equal to 'Stenotic' answer this question:***<br>Location stenosis	☐ Subclavian vein<br>☐ Axillary vein<br>☐ Brachial vein<br>☐ Cephalic vein<br>☐ Basilic vein<br>☐ Brachiocephalic vein
1.2.20.1.2	***If 'Vein patency, in rest' is equal to 'Occluded' answer this question:***<br>Location occlusion	☐ Subclavian vein<br>☐ Axillary vein<br>☐ Brachial vein<br>☐ Cephalic vein<br>☐ Basilic vein<br>☐ Brachiocephalic vein
2	duplex provocation & venous	
2.1	***If 'duplex provocation & venous' is equal to '1' answer this question:***<br>Vein compression with provocative maneuvres	○ Conform assesment in rest<br>○ Compression, 1-50% lumen reduction<br>○ Compression, 51-99% lumen reduction<br>○ Compression, not patent<br>○ Not judgeable
3	Vein compression duplex	
3.1	***If 'Vein compression duplex' is equal to '1' answer this question:***<br>Location compression	○ Costoclavicular junction<br>○ Pectoralis minor space<br>○ Both costoclavicular junction and pectoralis minor space
1.2.20.2	***If 'Duplex performed' is not equal to 'Venous' answer this question:***<br>Arterial patency, in rest	☐ Patent<br>☐ Stenotic<br>☐ Occluded
1.2.20.2.1	***If 'Arterial patency, in rest' is equal to 'Stenotic' answer this question:***<br>Stenosis of	☐ Subclavian artery<br>☐ Axillary artery<br>☐ Brachial artery<br>☐ Radial artery<br>☐ Ulnar artery
1.2.20.2.2	***If 'Arterial patency, in rest' is equal to 'Occluded' answer this question:***<br>Occlusion of	☐ Subclavian artery<br>☐ Axillary artery<br>☐ Brachial artery<br>☐ Radial artery<br>☐ Ulnar artery

1.2.20.3	***If 'Duplex performed' is not equal to 'Venous' answer this question:***<br>Aneurysm present	○ Yes<br>○ No
1.2.20.3.1	***If 'Aneurysm present' is equal to 'Yes' answer this question:***<br>Location aneurysm?	☐ Subclavian artery<br>☐ Axillary artery<br>☐ Brachial artery<br>☐ Radial artery<br>☐ Ulnar artery
1.2.20.3.2	***If 'Aneurysm present' is equal to 'Yes' answer this question:***<br>Thrombus present in aneurysm?	○ Yes<br>○ No<br>○ Unknown
1.2.20.3.3	***If 'Aneurysm present' is equal to 'Yes' answer this question:***<br>Maximum diameter of aneurysm	[ ] millimeter
4	Duplex provocation & arterial	
4.1	***If 'Duplex provocation & arterial' is equal to '1' answer this question:***<br>Arterial compression with provocative maneuvres	○ Conform assesment in rest<br>○ Compression, 1-50% lumen reduction<br>○ Compression, 51-99% lumen reduction<br>○ Compression, not patent<br>○ Not judgeable
5	arterial compression duplex	
5.1	***If 'arterial compression duplex' is equal to '1' answer this question:***<br>Location compression	○ Costoclavicular junction<br>○ Pectoralis minor space<br>○ Both costoclavicular junction and pectoralis minor space
1.2.20.4	***If 'Duplex performed' is not equal to 'Venous' answer this question:***<br>Distal arterial doppler signals, in rest	○ Normal (bi- or trifasic)<br>○ Abnormal<br>○ No flow<br>○ Not tested
4.2	***If 'Duplex provocation & arterial' is equal to '1' answer this question:***<br>Distal arterial doppler signals with provocative testing	○ Conform assesment in rest<br>○ Decreased signals<br>○ No flow
	__ATTENTION: please don't press 'Close report', press 'next' instead to go to the next page and complete the follow-up form before returning to the eCRF__	

Form Diagnostic modalities II

Number	Question	Answers
1	Calc diagnostics	
2	Calc CT	
3	Calc MRI	
	No diagnostic modalities used, please continue	
2.1	***If 'Calc CT' is equal to '1' answer this question:***<br>Date CT *Warning shown if field's value is larger than NOW: 'Date cannot be in the future'*	[ ] [ ] [ ] *(dd-mm-yyyy)*
2.2	***If 'Calc CT' is equal to '1' answer this question:***<br>Was CT performed with provocative maneuvres?	○ Yes<br>○ No
2.3	***If 'Calc CT' is equal to '1' answer this question:***<br>Arterial and/or venous CT?	○ Arterial<br>○ Venous<br>○ Arterial & venous
2.3.1	***If 'Arterial and/or venous CT?' is not equal to 'Arterial' answer this question:***<br>Ipsilateral vein patency with arm adducted	☐ Patent<br>☐ Stenotic<br>☐ Occluded
2.3.1.1	***If 'Ipsilateral vein patency with arm adducted' is equal to 'Stenotic' answer this question:***<br>Location venous stenosis	☐ Subclavian vein<br>☐ Axillary vein<br>☐ Brachial vein<br>☐ Cephalic vein<br>☐ Basilic vein<br>☐ Brachiocephalic vein
2.3.1.2	***If 'Ipsilateral vein patency with arm adducted' is equal to 'Occluded' answer this question:***<br>Location venous occlusion	☐ Subclavian vein<br>☐ Axillary vein<br>☐ Brachial vein<br>☐ Cephalic vein<br>☐ Basilic vein<br>☐ Brachiocephalic vein
2.2.1	***If 'Was CT performed with provocative maneuvres?' is equal to 'Yes' answer this question:***<br>Ipsilateral subclavian vein compression with arm abducted	○ Not judgeable/not scanned<br>○ Conform assesment in rest<br>○ Compression, 1-50% diameter reduction<br>○ Compression, 51-99% diameter reduction<br>○ Compression, 100% diameter reduction

2.2.2	***If 'Was CT performed with provocative maneuvres?' is equal to 'Yes' answer this question:***<br>Contralateral subclavian vein compression with arm abducted	◯ Not judgeable/not scanned<br>◯ Conform assesment in rest<br>◯ Compression, 1-50% diameter reduction<br>◯ Compression, 51-99% diameter reduction<br>◯ Compression, 100% diameter reduction
4	calc ipsilateral compression CT	
5	Calc contralteral compression CT	
4.1	***If 'calc ipsilateral compression CT' is equal to '1' answer this question:***<br>Location ipsilateral venous compression	◯ Costoclavicular junction<br>◯ Pectoralis minor space<br>◯ Both costoclavicular junction and pectoralis minor space
5.1	***If 'Calc contralteral compression CT' is equal to '1' answer this question:***<br>Location contralteral venous compression	◯ Costoclavicular junction<br>◯ Pectoralis minor space<br>◯ Both costoclavicular junction and pectoralis minor space
2.3.2	***If 'Arterial and/or venous CT?' is not equal to 'Venous' answer this question:***<br>Which ipsilateral arteries were assesed?	☐ Subclavian artery<br>☐ Axillary artery<br>☐ Brachial artery<br>☐ Ulnar artery<br>☐ Radial artery<br>☐ Hand arch<br>☐ Digital arteries
2.3.2.1	***If 'Which ipsilateral arteries were assesed?' is equal to 'Subclavian artery' answer this question:***<br>ipsilateral Subclavian artery with arm adducted	☐ Normal<br>☐ Thrombus present<br>☐ Stenotic<br>☐ Occluded<br>☐ Aneurysm present
2.3.2.1.1	***If 'ipsilateral Subclavian artery with arm adducted' is equal to 'Aneurysm present' answer this question:***<br>Thrombus present in aneurysm	☐ Yes<br>☐ No<br>☐ Unknown
2.3.2.1.2	***If 'ipsilateral Subclavian artery with arm adducted' is equal to 'Aneurysm present' answer this question:***<br>Maximum diameter of aneurysm	[ ] milimeter

2.2.3	***If 'Was CT performed with provocative maneuvres?' is equal to 'Yes' answer this question:***<br>Ipsilateral subclavian artery compression with arm abducted	○ Not judgeable/not scanned<br>○ Conform assesment in rest<br>○ Compression, 1-50% diameter reduction<br>○ Compression, 51-99% diameter reduction<br>○ Compression, 100% diameter reduction
2.2.4	***If 'Was CT performed with provocative maneuvres?' is equal to 'Yes' answer this question:***<br>Contralateral subclavian artery compression with arm abducted	○ Not judgeable/not scanned<br>○ Conform assesment in rest<br>○ Compression, 1-50% diameter reduction<br>○ Compression, 51-99% diameter reduction<br>○ Compression, 100% diameter reduction
2.3.2.2	***If 'Which ipsilateral arteries were assesed?' is equal to 'Axillary artery' answer this question:***<br>Ipsilateral axillary artery	☐ Normal<br>☐ Thrombus present<br>☐ Stenotic<br>☐ Occluded
2.3.2.3	***If 'Which ipsilateral arteries were assesed?' is equal to 'Brachial artery' answer this question:***<br>Ipsilateral brachial artery	☐ Normal<br>☐ Thrombus present<br>☐ Stenotic<br>☐ Occluded
2.3.2.4	***If 'Which ipsilateral arteries were assesed?' is equal to 'Ulnar artery' answer this question:***<br>Ipsilateral ulnar artery	☐ Normal<br>☐ Thrombus present<br>☐ Stenotic<br>☐ Occluded
2.3.2.5	***If 'Which ipsilateral arteries were assesed?' is equal to 'Radial artery' answer this question:***<br>Ipsilateral radial artery	☐ Normal<br>☐ Thrombus present<br>☐ Stenotic<br>☐ Occluded
2.3.2.6	***If 'Which ipsilateral arteries were assesed?' is equal to 'Hand arch' answer this question:***<br>Ipsilateral hand arch	☐ Normal<br>☐ Thrombus present<br>☐ Stenotic<br>☐ Occluded
2.3.2.7	***If 'Which ipsilateral arteries were assesed?' is equal to 'Digital arteries' answer this question:***<br>Ipsilateral digital arteries	☐ Normal<br>☐ Thrombus present<br>☐ Stenotic<br>☐ Occluded

2.3.2.7.1	***If 'Ipsilateral digital arteries' is not equal to 'Normal' answer this question:***<br>Which digiti are affected?	☐ Dig 1<br>☐ Dig 2<br>☐ Dig 3<br>☐ Dig 4<br>☐ Dig 5
2.4	***If 'Calc CT' is equal to '1' answer this question:***<br>Other relevant CT findings?	
3.1	***If 'Calc MRI' is equal to '1' answer this question:***<br>date MRI	__ __ __ *(dd-mm-yyyy)*
3.2	***If 'Calc MRI' is equal to '1' answer this question:***<br>Was MRI performed with provocative testing	○ Yes<br>○ No
3.3	***If 'Calc MRI' is equal to '1' answer this question:***<br>Arterial and/or venous?	○ Arterial<br>○ Venous<br>○ Arterial & venous
3.3.1	***If 'Arterial and/or venous?' is not equal to 'Arterial' answer this question:***<br>Ipsilateral vein patency with arm adducted	☐ Patent<br>☐ Stenotic<br>☐ Occluded
3.3.1.1	***If 'Ipsilateral vein patency with arm adducted' is equal to 'Stenotic' answer this question:***<br>Location venous stenosis	☐ Subclavian vein<br>☐ Axillary vein<br>☐ Brachial vein<br>☐ Cephalic vein<br>☐ Basilic vein<br>☐ Brachiocephalic vein
3.3.1.2	***If 'Ipsilateral vein patency with arm adducted' is equal to 'Occluded' answer this question:***<br>Location venous occlusion	☐ Subclavian vein<br>☐ Axillary vein<br>☐ Brachial vein<br>☐ Cephalic vein<br>☐ Basilic vein<br>☐ Brachiocephalic vein
3.2.1	***If 'Was MRI performed with provocative testing' is equal to 'Yes' answer this question:***<br>Ipsilateral subclavian vein compression with arm abducted	○ Not judgeable/not scanned<br>○ Conform assesment in rest<br>○ Compression, 1-50% diameter reduction<br>○ Compression, 51-99% diameter reduction<br>○ Compression, 100% diameter reduction

3.2.2	***If 'Was MRI performed with provocative testing' is equal to 'Yes' answer this question:***<br>Contralateral subclavian vein compression with arm abducted	○ Not judgeable/not scanned<br>○ Conform assesment in rest<br>○ Compression, 1-50% diameter reduction<br>○ Compression, 51-99% diameter reduction<br>○ Compression, 100% diameter reduction
6	calc ipsilateral compression MRI	
7	calc contralteral compression MRI	
6.1	***If 'calc ipsilateral compression MRI' is equal to '1' answer this question:***<br>Location ipsilateral venous compression	○ Costoclavicular junction<br>○ Pectoralis minor space<br>○ Both costoclavicular junction and pectoralis minor space
7.1	***If 'calc contralteral compression MRI' is equal to '1' answer this question:***<br>Location contralateral venous compression	○ Costoclavicular junction<br>○ Pectoralis minor space<br>○ Both costoclavicular junction and pectoralis minor space
3.3.2	***If 'Arterial and/or venous?' is not equal to 'Venous' answer this question:***<br>Which ipsilateral arteries were assesed?	☐ Subclavian artery<br>☐ Axillary artery<br>☐ Brachial artery<br>☐ Ulnar artery<br>☐ Radial artery<br>☐ Hand arch<br>☐ Digital arteries
3.3.2.1	***If 'Which ipsilateral arteries were assesed?' is equal to 'Subclavian artery' answer this question:***<br>Ipsilateral subclavian artery with arm adducted	☐ Normal<br>☐ Thrombus present<br>☐ Stenotic<br>☐ Occluded<br>☐ Aneurysm present
3.3.2.1.1	***If 'Ipsilateral subclavian artery with arm adducted' is equal to 'Aneurysm present' answer this question:***<br>Thrombus present in aneurysm?	○ Yes<br>○ No<br>○ Unknown
3.3.2.1.2	***If 'Ipsilateral subclavian artery with arm adducted' is equal to 'Aneurysm present' answer this question:***<br>Maximum diameter of aneurysm	[ ] millimeter

3.2.3	***If 'Was MRI performed with provocative testing' is equal to 'Yes' answer this question:***<br>Ipsilateral subclavian artery compression with arm abducted	○ Not judgeable/not scanned<br>○ Conform assesment in rest<br>○ Compression, 1-50% diameter reduction<br>○ Compression, 51-99% diameter reduction<br>○ Compression, 100% diameter reduction
3.2.4	***If 'Was MRI performed with provocative testing' is equal to 'Yes' answer this question:***<br>Contralateral subclavian artery compression with arm abducted	○ Not judgeable/not scanned<br>○ Conform assesment in rest<br>○ Compression, 1-50% diameter reduction<br>○ Compression, 51-99% diameter reduction<br>○ Compression, 100% diameter reduction
3.3.2.2	***If 'Which ipsilateral arteries were assesed?' is equal to 'Axillary artery' answer this question:***<br>Ipsilateral axillary artery	☐ Normal<br>☐ Thrombus present<br>☐ Stenotic<br>☐ Occluded
3.3.2.3	***If 'Which ipsilateral arteries were assesed?' is equal to 'Brachial artery' answer this question:***<br>Ipsilateral brachial artery	☐ Normal<br>☐ Thrombus present<br>☐ Stenotic<br>☐ Occluded
3.3.2.4	***If 'Which ipsilateral arteries were assesed?' is equal to 'Ulnar artery' answer this question:***<br>Ipsilateral ulnar artery	☐ Normal<br>☐ Thrombus present<br>☐ Stenotic<br>☐ Occluded
3.3.2.5	***If 'Which ipsilateral arteries were assesed?' is equal to 'Radial artery' answer this question:***<br>Ipsilateral radial artery	☐ Normal<br>☐ Thrombus present<br>☐ Stenotic<br>☐ Occluded
3.3.2.6	***If 'Which ipsilateral arteries were assesed?' is equal to 'Hand arch' answer this question:***<br>Ipsilateral hand arch	☐ Normal<br>☐ Thrombus present<br>☐ Stenotic<br>☐ Occluded
3.3.2.7	***If 'Which ipsilateral arteries were assesed?' is equal to 'Digital arteries' answer this question:***<br>Ipsilateral digital arteries	☐ Normal<br>☐ Thrombus present<br>☐ Stenotic<br>☐ Occluded

3.3.2.7.1	***If 'Ipsilateral digital arteries' is not equal to 'Normal' answer this question:***<br>Which digiti are affected?	☐ Dig 1<br>☐ Dig 2<br>☐ Dig 3<br>☐ Dig 4<br>☐ Dig 5
3.4	***If 'Calc MRI' is equal to '1' answer this question:***<br>Other relevant MR findings?	
	__ATTENTION: please don't press 'Close report', press 'next' instead to go to the next page and complete the follow-up form before returning to the eCRF__	

Form Diagnostic modalities III

Number	Question	Answers
1	Calc diagnostics	
2	Calc venography	
3	Calc arteriography	
4	Calc other diagnostic	
	No diagnostic modalities used, please continue	
2.1	***If 'Calc venography' is equal to '1' answer this question:***<br>Date venography	☐ ☐ ☐ *(dd-mm-yyyy)*
2.2	***If 'Calc venography' is equal to '1' answer this question:***<br>Was venography performed with provocative testing	○ Yes<br>○ No
2.2.1	***If 'Was venography performed with provocative testing' is equal to 'Yes' answer this question:***<br>Contralateral vein assessed with provocative testing?	○ Yes<br>○ No
2.3	***If 'Calc venography' is equal to '1' answer this question:***<br>Combined with intravascular ultrasound (IVUS)?	○ Yes<br>○ No
2.4	***If 'Calc venography' is equal to '1' answer this question:***<br>Vein patency, in rest	☐ Patent<br>☐ Stenotic<br>☐ Occluded

2.4.1	***If 'Vein patency, in rest' is equal to 'Stenotic' answer this question:***<br>Location stenosis	☐ Subclavian vein<br>☐ Axillary vein<br>☐ Brachial vein<br>☐ Cephalic vein<br>☐ Basilic vein<br>☐ Brachiocephalic vein
2.4.2	***If 'Vein patency, in rest' is equal to 'Occluded' answer this question:***<br>Location occlusion	☐ Subclavian vein<br>☐ Axillary vein<br>☐ Brachial vein<br>☐ Cephalic vein<br>☐ Basilic vein<br>☐ Brachiocephalic vein
2.2.2	***If 'Was venography performed with provocative testing' is equal to 'Yes' answer this question:***<br>Ipsilateral subclavian vein compression with provocative testing	○ Conform assesment in rest<br>○ Compression, 1-50% lumen reduction<br>○ Compression, 51-99% lumen reduction<br>○ Compression, not patent<br>○ Not judgeable
2.2.1.1	***If 'Contralateral vein assessed with provocative testing?' is equal to 'Yes' answer this question:***<br>Contralateral subclavian vein compression with provocative testing	○ Conform assesment in rest<br>○ Compression, 1-50% lumen reduction<br>○ Compression, 51-99% lumen reduction<br>○ Compression, not patent<br>○ Not judgeable
2.2.2.1	***If 'Ipsilateral subclavian vein compression with provocative testing' is not equal to 'Conform assesment in rest' answer this question:***<br>Location compression	○ Costoclavicular junction<br>○ Pectoralis minor space<br>○ Both costoclavicular junction and pectoralis minor space
2.5	***If 'Calc venography' is equal to '1' answer this question:***<br>Other relevant venography findings?	
2.3.1	***If 'Combined with intravascular ultrasound (IVUS)?' is equal to 'Yes' answer this question:***<br>Any relevant IVUS findings?	
3.1	***If 'Calc arteriography' is equal to '1' answer this question:***<br>Date arteriography	___ ___ ___ *(dd-mm-yyyy)*

3.2	***If 'Calc arteriography' is equal to '1' answer this question:*** Which arteries were assessed?	☐ Subclavian artery<br>☐ Axillary artery<br>☐ Brachial artery<br>☐ Ulnar artery<br>☐ Radial artery<br>☐ Hand arch<br>☐ Digital arteries
3.3	***If 'Calc arteriography' is equal to '1' answer this question:*** Was arteriography performed with provocative maneuvres?	○ Yes<br>○ No
3.3.1	***If 'Was arteriography performed with provocative maneuvres?' is equal to 'Yes' answer this question:*** Was contralateral subclavian artery assessed with provocative testing?	○ Yes<br>○ No
3.4	***If 'Calc arteriography' is equal to '1' answer this question:*** Combined with intravascular ultrasound (IVUS)?	○ Yes<br>○ No
3.2.1	***If 'Which arteries were assessed?' is equal to 'Subclavian artery' answer this question:*** Subclavian artery	☐ Not judgeable/not imaged<br>☐ Normal<br>☐ Thrombus present<br>☐ Stenotic<br>☐ Occluded<br>☐ Aneurysm present
3.2.1.1	***If 'Subclavian artery' is equal to 'Aneurysm present' answer this question:*** Thrombus present in aneurysm?	○ Yes<br>○ No<br>○ Unknown
3.2.1.2	***If 'Subclavian artery' is equal to 'Aneurysm present' answer this question:*** Maximum diamter of aneurysm (if nog measured, choose 0)	[ ] Milimeter
3.3.2	***If 'Was arteriography performed with provocative maneuvres?' is equal to 'Yes' answer this question:*** Ipsilateral subclavian artery with provocative maneuvres	○ Conform assesment in rest<br>○ Compression, 1-50% lumen reduction<br>○ Compression, 51-99% lumen reduction<br>○ Compression, not patent<br>○ Not judgeable
3.3.1.1	***If 'Was contralateral subclavian artery assessed with provocative testing?' is equal to 'Yes' answer this question:*** Contralateral subclavian artery with provocative maneuvres	○ Conform assesment in rest<br>○ Compression, 1-50% lumen reduction<br>○ Compression, 51-99% lumen reduction<br>○ Compression, not patent<br>○ Not judgeable

3.2.2	***If 'Which arteries were assessed?' is equal to 'Axillary artery' answer this question:***<br>Axillary artery	☐ Not judgeable/not imaged<br>☐ Normal<br>☐ Thrombus present<br>☐ Stenotic<br>☐ Occluded
3.2.3	***If 'Which arteries were assessed?' is equal to 'Brachial artery' answer this question:***<br>Brachial artery	☐ Not judgeable/not imaged<br>☐ Normal<br>☐ Thrombus present<br>☐ Stenotic<br>☐ Occluded
3.2.4	***If 'Which arteries were assessed?' is equal to 'Ulnar artery' answer this question:***<br>Ulnar artery	☐ Not judgeable/not imaged<br>☐ Normal<br>☐ Thrombus present<br>☐ Stenotic<br>☐ Occluded
3.2.5	***If 'Which arteries were assessed?' is equal to 'Radial artery' answer this question:***<br>Radial artery	☐ Not judgeable/not imaged<br>☐ Normal<br>☐ Thrombus present<br>☐ Stenotic<br>☐ Occluded
3.2.6	***If 'Which arteries were assessed?' is equal to 'Hand arch' answer this question:***<br>Hand arch	☐ Not judgeable/not imaged<br>☐ Normal<br>☐ Thrombus present<br>☐ Stenotic<br>☐ Occluded
3.2.7	***If 'Which arteries were assessed?' is equal to 'Digital arteries' answer this question:***<br>Digital arteries	☐ Not judgeable/not imaged<br>☐ Normal<br>☐ Thrombus present<br>☐ Stenotic<br>☐ Occluded
5	calc digiti affected	
5.1	***If 'calc digiti affected' is not equal to '1' answer this question:***<br>Which dig(iti) is/are affected?	☐ Dig 1<br>☐ Dig 2<br>☐ Dig 3<br>☐ Dig 4<br>☐ Dig 5

3.5	***If 'Calc arteriography' is equal to '1' answer this question:***<br>Other relevant arteriography findings?	
3.4.1	***If 'Combined with intravascular ultrasound (IVUS)?' is equal to 'Yes' answer this question:***<br>Any relevant IVUS findings?	
4.1	***If 'Calc other diagnostic' is equal to '1' answer this question:***<br>Please describe other diagnostic modality and findings	
1.2	***If 'Calc diagnostics' is equal to '1' answer this question:***<br>Anatomical abnormality present, left side	☐ No<br>☐ Cervical rib<br>☐ Prominent transverse process C7<br>☐ Abnormal clavicle<br>☐ Abnormal first rib<br>☐ Other<br>☐ Unknown
1.2.1	***If 'Anatomical abnormality present, left side' is equal to 'Other' answer this question:***<br>Please describe other anatomical abnormality, left side	
1.3	***If 'Calc diagnostics' is equal to '1' answer this question:***<br>Anatomical abnormality present, right side	☐ No<br>☐ Cervical rib<br>☐ Prominent transverse process C7<br>☐ Abnormal clavicle<br>☐ Abnormal first rib<br>☐ Other<br>☐ Unknown
1.3.1	***If 'Anatomical abnormality present, right side' is equal to 'Other' answer this question:***<br>Please describe other anatomical abnormality, right side	

__ATTENTION: please don't press 'Close report', press 'next' instead to go to the next page and complete the follow-up form before returning to the eCRF__

Form Treatment

Number	Question	Answers
1	calc treatment	
2	Was there a change in antithrombotic therapy since last contact? (if applicable)<br>If antithrombotic was changed for another antithrombotic (for example because of side effects), pleasse check antithrombotic stopped AND antithrombotic started.	☐ Yes, antithrombotic stopped<br>☐ Yes, antithrombotic started<br>☐ No<br>☐ Not applicable
2.1	***If 'Was there a change in antithrombotic therapy since last contact? (if applicable)' is equal to 'Yes, antithrombotic stopped' answer this question:***<br>Which antithrombotic(s) was stopped?	☐ Acetylsalicylic acid<br>☐ ADP inhibitor (e.g. clopidogrel)<br>☐ Vitamin K antagonist (e.g. acenocoumarol)<br>☐ Directly acting oral anticoagulants (DOACs)<br>☐ Low molecular weight heparin<br>☐ Other
2.1.1	***If 'Which antithrombotic(s) was stopped?' is equal to 'Other' answer this question:***<br>Which other antithrombotic	
2.1.2	***If 'Which antithrombotic(s) was stopped?' is equal to 'Acetylsalicylic acid' answer this question:***<br>Date acetylsalicylic acid stopped	*(dd-mm-yyyy)*
2.1.3	***If 'Which antithrombotic(s) was stopped?' is equal to 'ADP inhibitor (e.g. clopidogrel)' answer this question:***<br>Date ADP inhibitor stopped<br>If precise date is unknown:<br>5-2020 -> 01-05-2020<br>2020 -> 01-01-2020	*(dd-mm-yyyy)*
2.1.4	***If 'Which antithrombotic(s) was stopped?' is equal to 'Vitamin K antagonist (e.g. acenocoumarol)' answer this question:***<br>Date vitamin K antagonist stopped	*(dd-mm-yyyy)*
2.1.5	***If 'Which antithrombotic(s) was stopped?' is equal to 'Directly acting oral anticoagulants (DOACs)' answer this question:***<br>Date DOAC stopped<br>If precise date is unknown:<br>5-2020 -> 01-05-2020<br>2020 -> 01-01-2020	*(dd-mm-yyyy)*

2.1.6	***If 'Which antithrombotic(s) was stopped?' is equal to 'Low molecular weight heparin' answer this question:***<br>Date low molecular weight heparin stopped<br>If precise date is unknown:<br>5-2020 -> 01-05-2020<br>2020 -> 01-01-2020	☐ ☐ ☐ *(dd-mm-yyyy)*
2.1.7	***If 'Which antithrombotic(s) was stopped?' is equal to 'Other' answer this question:***<br>Date 'other antithrombotic' stopped<br>If precise date is unknown:<br>5-2020 -> 01-05-2020<br>2020 -> 01-01-2020	☐ ☐ ☐ *(dd-mm-yyyy)*
2.2	***If 'Was there a change in antithrombotic therapy since last contact? (if applicable)' is equal to 'Yes, antithrombotic started' answer this question:***<br>Which antithrombotic(s) was started?	☐ Acetylsalicylic acid<br>☐ ADP inhibitor (e.g. clopidogrel)<br>☐ Vitamin K antagonist (e.g. acenocoumarol)<br>☐ Directly acting oral anticoagulants (DOACs)<br>☐ Low molecular weight heparin<br>☐ Other
2.2.1	***If 'Which antithrombotic(s) was started?' is equal to 'Acetylsalicylic acid' answer this question:***<br>Which acetylsalicylica acid was started, including dosage	○ Acetylsalicylic acid 30mg (neuro)<br>○ Acetylsalicylic acid 80mg (cardio)<br>○ Acetylsalicylic acid 100mg (aspirin protect)<br>○ Carbasalate calcium 38mg (ascal 38)<br>○ Carbasalate calcium 100mg (ascal cardio)<br>○ Other
2.2.1.1	***If 'Which acetylsalicylica acid was started, including dosage' is equal to 'Other' answer this question:***<br>Which other acetylsalicylic acid, including dosage	☐
2.2.2	***If 'Which antithrombotic(s) was started?' is equal to 'Acetylsalicylic acid' answer this question:***<br>Date acetylsalicylic acid started<br>If precise date is unknown:<br>5-2020 -> 01-05-2020<br>2020 -> 01-01-2020	☐ ☐ ☐ *(dd-mm-yyyy)*
2.2.3	***If 'Which antithrombotic(s) was started?' is equal to 'ADP inhibitor (e.g. clopidogrel)' answer this question:***<br>Which ADP inhibitor was started, including dosage	○ Clopidogrel 75mg<br>○ Ticagrelor 60mg<br>○ Ticagrelor 90mg<br>○ Prasugrel 5mg<br>○ Prasugrel 10mg<br>○ Other

2.2.3.1	***If 'Which ADP inhibitor was started, including dosage' is equal to 'Other' answer this question:***<br>Which other ADP inhibitor, including dosage	
2.2.4	***If 'Which antithrombotic(s) was started?' is equal to 'ADP inhibitor (e.g. clopidogrel)' answer this question:***<br>Date ADP inhibitor started<br>If precise date is unknown:<br>5-2020 -> 01-05-2020<br>2020 -> 01-01-2020	*(dd-mm-yyyy)*
2.2.5	***If 'Which antithrombotic(s) was started?' is equal to 'Vitamin K antagonist (e.g. acenocoumarol)' answer this question:***<br>Which vitamin K antagonist was started?	◯ Acenocoumarol<br>◯ Phenprocoumon<br>◯ Warfarin<br>◯ Other
2.2.5.1	***If 'Which vitamin K antagonist was started?' is equal to 'Other' answer this question:***<br>Which other vitamin K antagonist?	
2.2.6	***If 'Which antithrombotic(s) was started?' is equal to 'Vitamin K antagonist (e.g. acenocoumarol)' answer this question:***<br>Date vitamin K antagonist started<br>If precise date is unknown:<br>5-2020 -> 01-05-2020<br>2020 -> 01-01-2020	*(dd-mm-yyyy)*
2.2.7	***If 'Which antithrombotic(s) was started?' is equal to 'Directly acting oral anticoagulants (DOACs)' answer this question:***<br>Which DOAC was started, including dosage	◯ Rivaroxaban, 15mg twice daily for 21 days followed by 20mg daily<br>◯ Rivaroxaban 20mg daily<br>◯ Rivaroxaban 10mg daily<br>◯ Rivaroxaban 2.5mg twice daily<br>◯ Apixaban 10mg twice daily for 7 days followed by 5mg twice daily<br>◯ Apixaban 5mg twice daily<br>◯ Apixaban 2.5mg twice daily<br>◯ Edoxaban 60mg daily<br>◯ Dabigatran 150mg twice daily<br>◯ Other

2.2.7.1	***If 'Which DOAC was started, including dosage' is equal to 'Other' answer this question:***<br>Which other DOAC, including dosage	
2.2.8	***If 'Which antithrombotic(s) was started?' is equal to 'Directly acting oral anticoagulants (DOACs)' answer this question:***<br>Date DOAC started<br>If precise date is unknown:<br>5-2020 -> 01-05-2020<br>2020 -> 01-01-2020	*(dd-mm-yyyy)*
2.2.9	***If 'Which antithrombotic(s) was started?' is equal to 'Low molecular weight heparin' answer this question:***<br>Which low molecular weight heparin was started, including dosage	◯ Dalteparin (Fragmin) 200 IU/kg once daily<br>◯ Nadroparin (Fraxiparine) 86 UI/kg twice daily<br>◯ Enoxaparin (Clexane) 150UI/kg once daily<br>◯ Tinzaparin (Innohep) 175 UI/kg once daily<br>◯ Other
2.2.9.1	***If 'Which low molecular weight heparin was started, including dosage' is equal to 'Other' answer this question:***<br>Which other low molecular weight heparin, including dosage	
2.2.10	***If 'Which antithrombotic(s) was started?' is equal to 'Low molecular weight heparin' answer this question:***<br>Date low molecular weight heparin started<br>If precise date is unknown:<br>5-2020 -> 01-05-2020<br>2020 -> 01-01-2020	*(dd-mm-yyyy)*
2.2.11	***If 'Which antithrombotic(s) was started?' is equal to 'Other' answer this question:***<br>Which other antithrombotic was started, including dosage	
2.2.12	***If 'Which antithrombotic(s) was started?' is equal to 'Other' answer this question:***<br>Date 'other antithrombotic' started<br>If precise date is unknown:<br>5-2020 -> 01-05-2020<br>2020 -> 01-01-2020	*(dd-mm-yyyy)*

1.1	***If 'calc treatment' is equal to '1' answer this question:***<br>What treatment(s) was/were started or performed?	☐ Physical therapy<br>☐ Compression therapy<br>☐ Mensendieck therapy<br>☐ Medication besides antithrombotics<br>☐ Change in antithrombotic treatment (above)<br>☐ Local injection with botox/anesthetic<br>☐ Endovascular intervention(s)<br>☐ Surgical intervention(s)<br>☐ Other
	__Medication started__	
1.1.2	***If 'What treatment(s) was/were started or performed?' is equal to 'Medication besides antithrombotics' answer this question:***<br>Medication started (besides antithrombotics)	
	__Local injection__	
1.1.4	***If 'What treatment(s) was/were started or performed?' is equal to 'Local injection with botox/anesthetic' answer this question:***<br>Date local injection	*(dd-mm-yyyy)*
1.1.5	***If 'What treatment(s) was/were started or performed?' is equal to 'Local injection with botox/anesthetic' answer this question:***<br>Which agent was injected	☐ Botulinium toxin<br>☐ Steroids<br>☐ Local anesthesticum<br>☐ Other<br>☐ Unknown
1.1.5.1	***If 'Which agent was injected' is equal to 'Other' answer this question:***<br>Which other agent	
1.1.6	***If 'What treatment(s) was/were started or performed?' is equal to 'Local injection with botox/anesthetic' answer this question:***<br>Dosage of agent	
1.1.7	***If 'What treatment(s) was/were started or performed?' is equal to 'Local injection with botox/anesthetic' answer this question:***<br>Which structure was injected?	☐ Anterior scalene muscle<br>☐ Middle scalene muscle<br>☐ Subclavius muscle<br>☐ Pectoralis minor<br>☐ Other<br>☐ Unknown

1.1.7.1	***If 'Which structure was injected?' is equal to 'Other' answer this question:***<br>Which other structure	
1.1.8	***If 'What treatment(s) was/were started or performed?' is equal to 'Local injection with botox/anesthetic' answer this question:***<br>Which technique was used?	☐ Landmarks<br>☐ EMG guidance<br>☐ Fluoroscopic guidande<br>☐ Ultrasound<br>☐ CT<br>☐ MRI<br>☐ Other<br>☐ Unknown
1.1.8.1	***If 'Which technique was used?' is equal to 'Other' answer this question:***<br>Which other technique	
	__Endovascular intervention(s)__	
	__If endovascular procedure was performed simultaneously with surgery, this can be describe in the endovascular and surgical intervention form.__	
1.1.11	***If 'What treatment(s) was/were started or performed?' is equal to 'Endovascular intervention(s)' answer this question:***<br>Venous or arterial endovascular intervention?	☐ Venous<br>☐ Arterial
1.1.12	***If 'What treatment(s) was/were started or performed?' is equal to 'Endovascular intervention(s)' answer this question:***<br>Explanation	
1.1.11.1	***If 'Venous or arterial endovascular intervention?' is equal to 'Venous' answer this question:***<br>Venous endovascular intervention form	
1.1.11.2	***If 'Venous or arterial endovascular intervention?' is equal to 'Arterial' answer this question:***<br>Arterial endovascular intervention form	
	__Surgical intervention(s)__	
1.1.14	***If 'What treatment(s) was/were started or performed?' is equal to 'Surgical intervention(s)' answer this question:***<br>Explanation	
1.1.15	***If 'What treatment(s) was/were started or performed?' is equal to 'Surgical intervention(s)' answer this question:***<br>Surgical intervention form	
	__Other therapy or therapies__	

1.1.17	***If 'What treatment(s) was/were started or performed?' is equal to 'Other' answer this question:***<br>Please describe 'other' therapies in detail	

__When finished with the follow-up form, please click 'close report' to return to the eCRF. This form will remain visible and accessible in the left side of the screen.__
